# A Model of the Mechanisms Underpinning Unconventional Aqueous Humor Outflow

**DOI:** 10.1167/iovs.66.4.75

**Published:** 2025-04-28

**Authors:** Jennifer H. Tweedy, Mariia Dvoriashyna, Jessica R. Crawshaw, Darryl R. Overby, Rodolfo Repetto, Paul A. Roberts, Tamsin A. Spelman, Peter S. Stewart, Alexander J. E. Foss

**Affiliations:** 1 Department of Mathematical Sciences, University of Bath, Claverton Down, Bath, United Kingdom; 2 School of Mathematics and Maxwell Institute for Mathematical Sciences, University of Edinburgh, Edinburgh, United Kingdom; 3 Wolfson Centre for Mathematical Biology, Mathematical Institute, University of Oxford, Oxford, United Kingdom; 4 School of Mathematical Sciences, Queensland University of Technology, Brisbane, Australia; 5 Department of Bioengineering, Imperial College London, London, United Kingdom; 6 Department of Civil, Chemical and Environmental Engineering, University of Genoa, Genoa, Italy; 7 Department of Optometry and Visual Sciences, City St. George's, University of London, London, United Kingdom; 8 Sainsbury Laboratory, University of Cambridge, Cambridge, United Kingdom; 9 School of Mathematics & Statistics, University Place, University of Glasgow, Glasgow, United Kingdom; 10 Department of Ophthalmology, Nottingham University Hospitals NHS Trust, Nottingham, United Kingdom

**Keywords:** mathematical modeling, aqueous humor, unconventional outflow, glaucoma medications, prostaglandins

## Abstract

**Purpose:**

To develop a mathematical model of the unconventional outflow pathway.

**Methods:**

The unconventional pathway is modeled as having two key components: the uveo-vortex and the trans-scleral pathways. The uveo-vortex pathway is modeled using Starling’s law and the trans-scleral flow using predominately hydrostatic forces. We include transcytosis from the choriocapillaris (CC) and collapsibility of the suprachoroidal space (SCS) as particular features. There is considerable uncertainty in a number of model parameter values, and we identify the most significant ones using sensitivity analysis.

**Results:**

The model successfully generates a fluid flow from anterior to posterior in the choroidal tissue and the SCS, which also demonstrates many of the known physiological features, including the insensitivity of the unconventional flow to fluctuations in the IOP, albumin removal by the trans-scleral flow, and the CC as a net absorber of fluid from, and supplier of albumin to, the choroidal tissue. The model supports the two previously proposed mechanisms of the action of prostaglandin F_2α_ analogues.

**Conclusions:**

We have developed a theoretical model of the unconventional aqueous outflow pathway that successfully captures its physiological features and elucidates the actions of prostaglandin F_2α_ analogues and other drugs.

It has long been well-known that most of the aqueous humor drains from the eye via the trabecular meshwork and the canal of Schlemm, the so-called conventional pathway. The presence of a second outflow pathway, termed the unconventional flow (also known as the uveo-scleral flow), was first reported in 1903 by Leber; he observed that tracers introduced into the anterior chamber of the eye could traverse into the suprachoroidal space (SCS) between the choroid and sclera.^1^ However, it was not until the late 1960s that a clear description of the unconventional outflow pathway emerged, following a series of pioneering experiments by Bill et al.^2^^–^^7^ In particular, it was noted that tracer particles travel from the anterior chamber to the SCS at a rate 200-fold greater than the reverse movement in the cynomolgus monkey,^8^ and the rate is independent of particle size,^4^ indicating a bulk flow of fluid.

A schematic diagram of aqueous flow is shown in Figure 1. The ciliary epithelium, which overlies the ciliary processes, produces aqueous, from where it flows through the posterior chamber, anteriorly through the pupil (cyan arrows) into the anterior chamber and exits the eye via two distinct pathways: the conventional (green arrow) and unconventional routes (black arrows). The conventional flow traverses the trabecular meshwork, enters Schlemm's canal, and exits via the episcleral veins. The unconventional flow, which accounts for a sizable fraction (5% to 40%) of the total outflow,^9^ continues posteriorly through the choroidal tissue and leaves the eye via either the trans-scleral or uveo-vortex routes. As a point of terminology, we avoid the term uveo-scleral flow, because this has variably been used to describe either the unconventional pathway as a whole or just the trans-scleral pathway.

**Figure 1.**
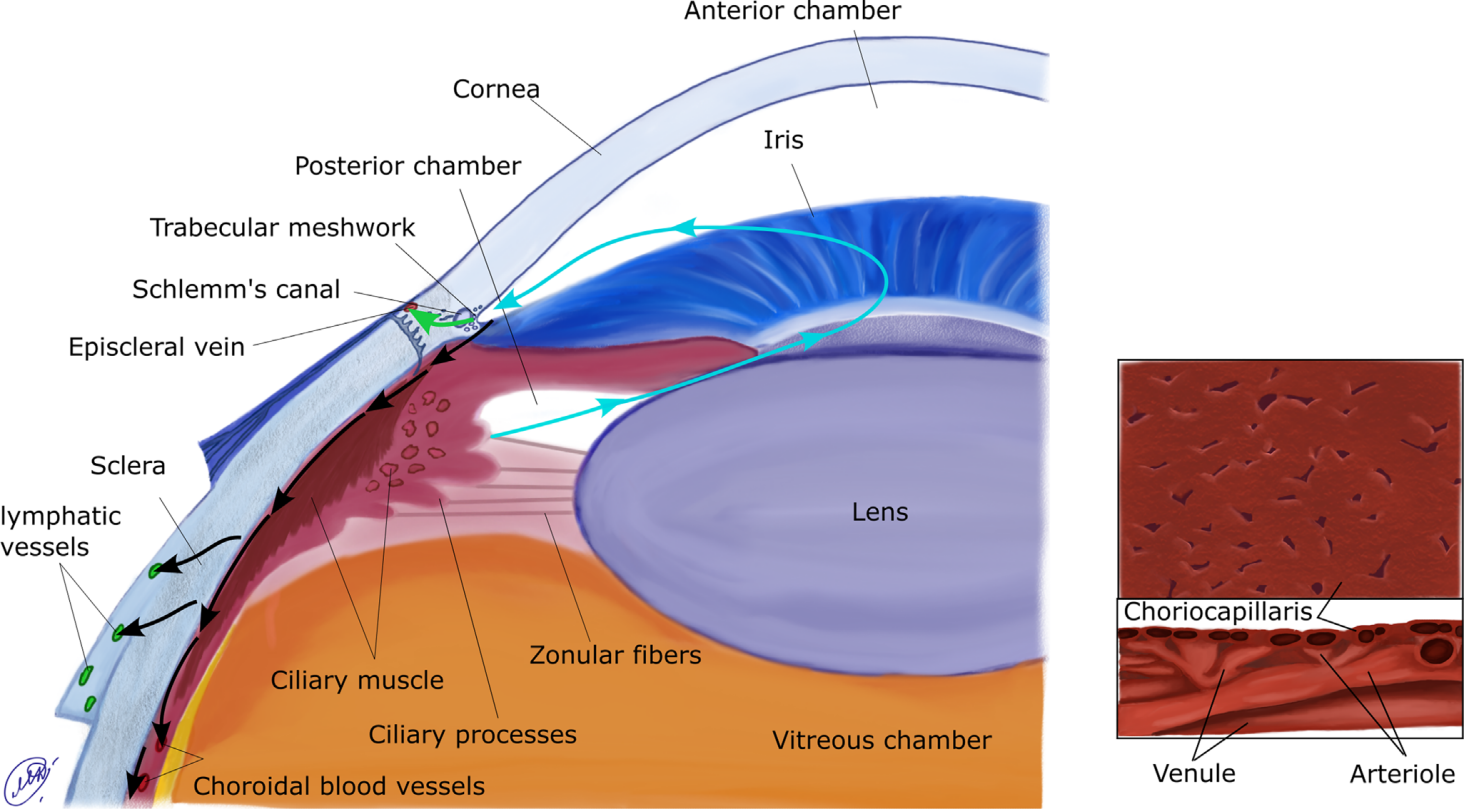
(*Left*) Schematic of the anterior segment of the eye. Aqueous humor flow in the posterior and anterior chambers is shown with cyan arrows. The conventional outflow is indicated by the green arrow, and the unconventional pathway is marked by black arrows. (*Right*) Zoom of the choroid (based on Fig. 4 by Wajer et al.^88^). (*Bottom*) Cross-section, showing the layers of different types of vessels; top: view of apical surface of the CC.

The trans-scleral flow is important as it removes choroidal interstitial albumin. In other tissues, typically, the lymphatic systems clears interstitial albumin, but this is absent in the choroid (although the presence of lymphatics in the ciliary body has been reported^10^; however, their existence remains controversial^11^). The concentration of albumin in the aqueous within the anterior chamber is estimated at around only 1% of the plasma level,^12^^,^^13^ but, on its passage through the tissue of the iris root, the interstitial fluid (IF) accumulates albumin from the fenestrated capillaries of the ciliary body stroma,^14^^,^^15^ meaning that, when it reaches the ciliary body, the level of albumin is much higher, with levels as high as 74% of that in plasma being reported.^16^^,^^17^ In other experiments, researchers have measured the albumin concentration in the uveal IF as around 10% of the plasma level in monkeys, 30% in rabbits and 19% to 35% in humans.^18^^,^^19^ Albumin from the plasma in the vessels of the choriocapillaris (CC) also accumulates in the IF. This is predominantly via transcytosis^20^ (see also the large pore system^21^), although transport through fenestrae and active transport play a minor role.^21^ The trans-scleral flow is dominated by direct (pressure-driven) flow across the sclera, although there is also a small amount of drainage through the perivascular spaces (by mechanisms still not well-understood).^22^^–^^24^ Indeed, the hydraulic conductivity of sclera is quite sufficient to account for a trans-scleral flow without invoking special routes.^25^

Starling forces arise owing to a combination of local hydrostatic and osmotic pressure digerences between blood in the capillaries and the surrounding IF. In most capillaries, the balance of these forces favors net exudation from the vessels,^26^ owing to the low interstitial hydrostatic pressure. In the CC, however, the balance favors net absorption owing to the high IOP. This uveo-vortex flow into the CC accounts for a large fraction of the total unconventional flow.^27^^,^^28^

Interest in the unconventional outflow increased following the discovery that prostaglandin F_2α_ (PGF_2α_) analogues, such as latanoprost, act by enhancing this flow, thus reducing the IOP. This class of drugs is the most frequent treatment for glaucoma, and there are two hypothesized mechanisms of their action. The first postulates a reduced pathway resistance through the ciliary muscle owing to either or both of relaxing it^29^ and opening intramuscular spaces.^30^ The opening of the spaces by PGF_2α_ analogues is thought to be due to release of matrix metalloproteases (MMPs) from the ciliary muscle, resulting in a degree of autodigestion of the ciliary body extracellular matrix.^31^ This process results in the appearance of extracellular spaces in the ciliary muscular tissue, which have been observed in histological samples taken within a few days of commencing treatment.^30^ The second mechanism also involves increased MMP expression, this time acting on the sclera to increase its hydraulic conductivity, which also increases the flow.^32^^,^^33^

However, we note that there are other potential mechanisms that could play a contributory role. For example, changes are reported in the vasculature when using PGF_2α_ analogues. These changes include acting as a vasoconstrictor or a vasodilator, but, for the eye, one of the commonest side effects is hyperemia, which is a vasodilatory response.^34^ In addition, there is increased hydraulic conductance of the capillary walls, and increases of up to three-fold have been reported;^35^^,^^36^ we also note that MMPs are involved in the regulation of the endothelial hydraulic conductance.^37^ Furthermore, latanoprost causes choroidal thickening, but the reason for this is not clear.^38^

Other drugs also have an effect on the unconventional pathway: atropine,^39^^,^^40^ tropicamide^41^ (both antagonists to the acetylcholine receptor) and epinephrine^42^ all enhance the flow, whereas pilocarpine^43^^,^^40^ (an agonist to the acetylcholine receptor) reduces the flow. It has been proposed that atropine and pilocarpine act by affecting the resistance to flow through the ciliary muscle. However, these drugs can also act on the vasculature: pilocarpine increases blood vessel hydraulic conductance,^44^ tropicamide reduces vessel albumin conductance, and epinephrine decreases blood vessel hydraulic conductance. In this article, we explore how changes in the vasculature affect unconventional flow.

A large increase in unconventional flow is required to reduce the IOP significantly, for the following reasons. To reduce the IOP by *r*%, we need to reduce the hydrostatic pressure drop across the trabecular meshwork and Schlemm’s canal, $\mathrm{IOP}-p_{E}$ (where $p_{E}$ is episcleral venous pressure) by a larger proportion *r*_*d*_%, given by $r_{d}=\mathrm{IOP}\cdot r/\left(\mathrm{IOP}-p_{E}\right)$. The conventional outflow is proportional to this pressure drop and thus also reduces by *r*_*d*_%.^45^ The remainder of the aqueous flow must be taken up by the unconventional pathway, meaning that its flow must grow by *Q*_*c*_*r*_*d*_/*Q*_*u*_%, where *Q*_*c*_ and *Q*_*u*_ are the flows (before IOP reduction) in the conventional and unconventional pathways, respectively. Thus, to achieve a moderate (say *r* = 20%) reduction in the IOP requires a percentage increase in the unconventional flow that is many times greater, both as *Q*_*c*_/*Q*_*u*_ is many times greater than unity and also as *r*_*d*_ > *r*. A further aim of this work is, therefore, to explore in general the mechanisms by which unconventional flow could be enhanced.

The unconventional drainage pathway has the counter-intuitive property of being relatively unaffected by the IOP.^3^^,^^5^^,^^46^ This is in contrast with the conventional flow, which increases in proportion to the difference between the IOP and the episcleral venous pressure. Bill,^5^ working on living cynomolgus monkeys, found that at a physiological IOP (11 mm Hg) the conventional and unconventional flow rates were 0.80 ± 0.11 µL/min and 0.44 ± 0.06 µL/min, respectively, but when the IOP was increased artificially to 22 mm Hg, these rates increased to 4.18 ± 0.12 µL/min and 0.63 ± 0.08 µL/min, respectively. This change represents a fivefold increase in the conventional flow and less than 50% increase in the unconventional flow (Fig. 5A).

**Figure 2.**
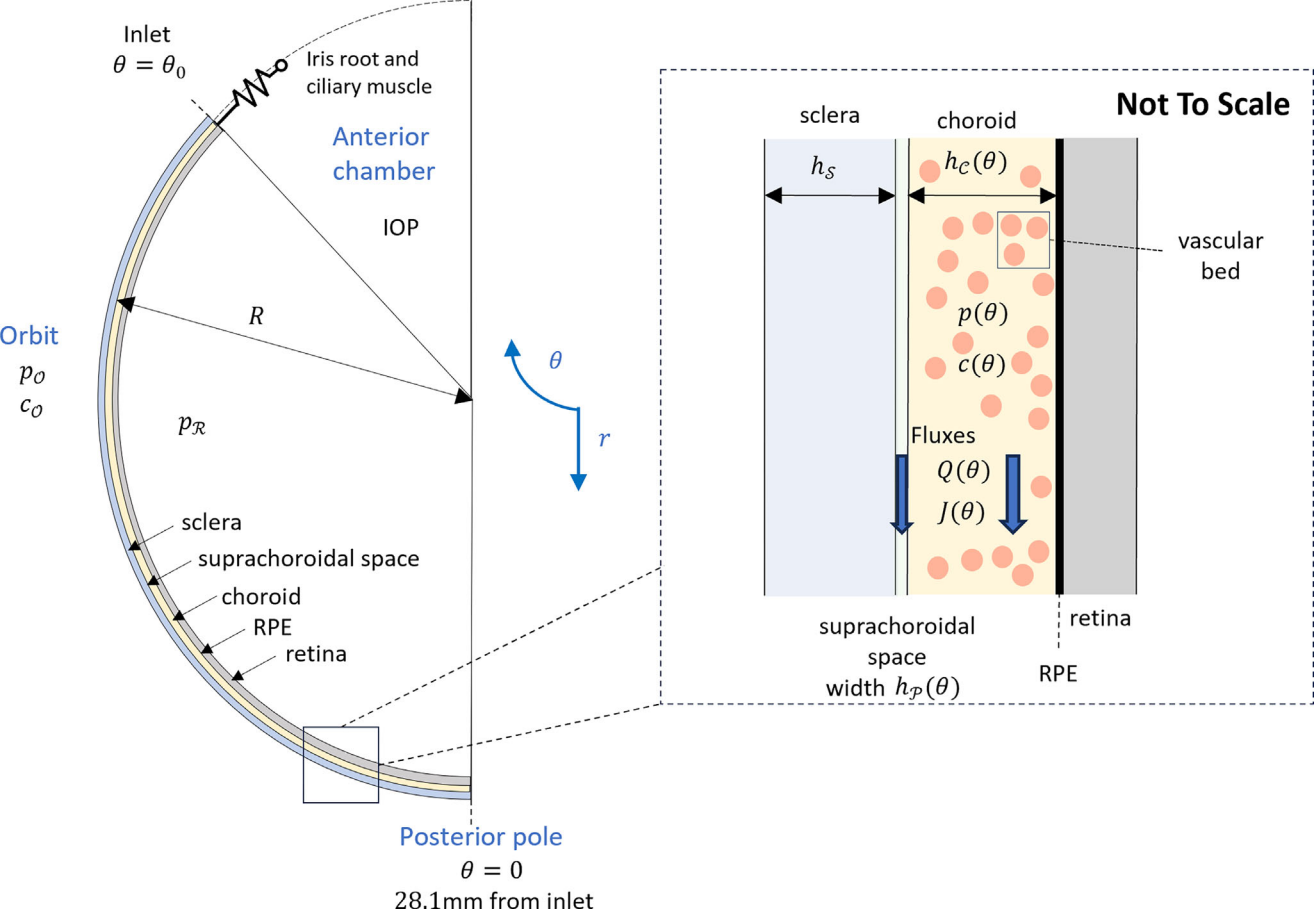
Schematic diagram of the eye highlighting the setup and parameters used in the mathematical model. The anterior of the eye is at the top of the diagram and the model has rotational symmetry about the vertical axis θ = 0. The inset zooms into the region we focus on in the model, which is the outer part of the posterior eye.

**Figure 3.**
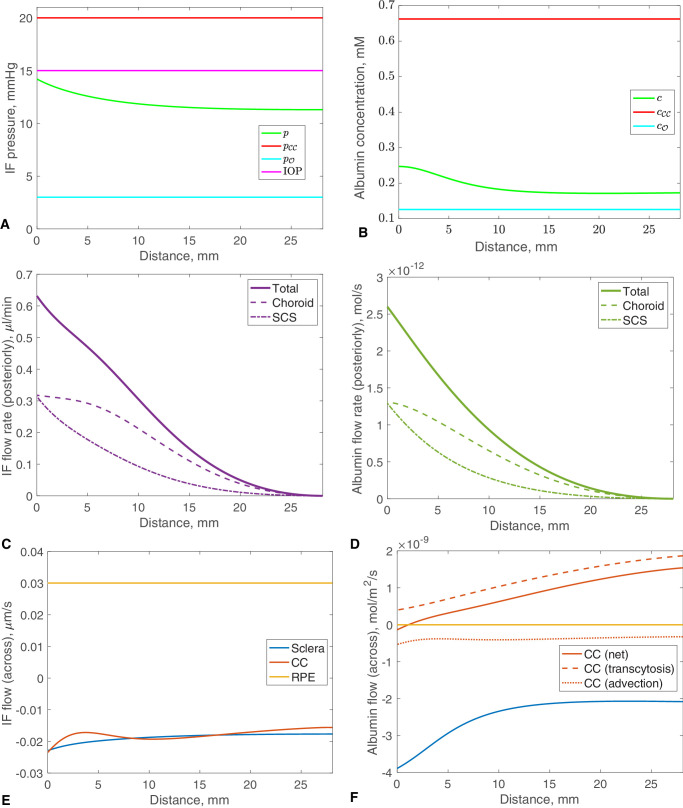
Model results in the reference physiological case, showing the IF flow and albumin concentration plotted against distance from the inlet. (**A**) Pressure in the choroid–SCS, *p*, CC, $p_{\mathrm{CC}}$, orbit, $p_{O}$, and anterior chamber, IOP. (**B**) Albumin concentration in the choroid–SCS, *c*, CC, $c_{\mathrm{CC}}$, and orbit, $c_{O}$. (**C**) IF flow rate posteriorly in the choroid–SCS, showing also the components of this flow in the choroidal tissue and SCS. (**D**) As (**C**), but for the albumin flow rate. (**E**) IF flow rate per unit area of surface into (positive) and out of (negative) the choroid–SCS. (**F**) As (**E**), but for albumin (with the same colors). The flow of albumin out of the CC is the sum of the contributions due to transcytosis (out of the CC, positive) and to advection by the crystalloid fluid (into the CC, negative).

**Figure 4.**
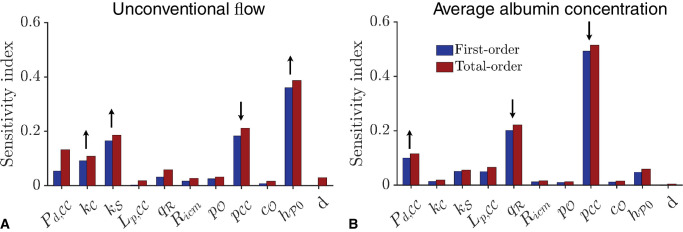
Results of sensitivity analysis showing the first-order (*blue*) and total-order (*red*) sensitivities for (**A**) the unconventional flow rate, and (**B**) the average albumin concentration across the choroid–SCS. The directions of influence for the most sensitive parameters are indicated by the *black arrows* above the bars, that is, an *upward (downward) arrow* indicates that the flow rate/albumin concentration increases (decreases) as the given parameter is increased. The label d is the dummy variable, which is a parameter that does not appear in the model and hence represents a ‘negative control’ for the sensitivity analysis. In the simulations, $k_{S}$ is varied independently of $k_{C}$.

**Figure 5.**
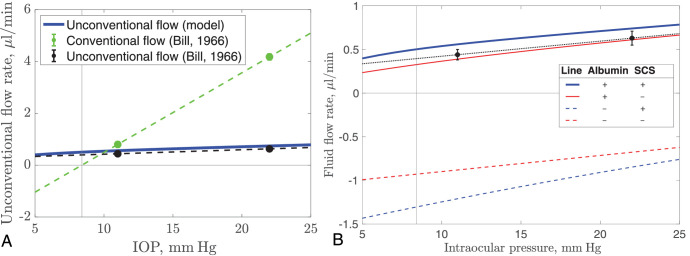
Dependence of the unconventional flow rate on the IOP. (**A**) Comparison of conventional and unconventional flow rates. *Solid dots*: Measurements of the conventional (*green*) and unconventional (*black*) flow rates by Bill on living monkeys with best fit *dashed lines* added.^5^ *Blue curve*: Predictions of the model. (**B**) Detailed graph of predictions of the model accounting for various mechanisms. In the table in the figure inset, the column Albumin indicates the presence or absence of albumin exchange with the vessels: + indicates that there is exchange; − that there is not (we set $\sigma_{\mathrm{CC}}=\sigma_{S}=0$ in the model). The column SCS refers to the second mechanism: The symbols + and − indicate the presence or absence of a collapsible SCS, respectively. The *black dashed line* and *blue solid curve* are the same as those appearing in (**A**). In both figures, the *black vertical line* corresponds with the episcleral venous pressure of 8.4 mm Hg and the *black horizontal line* marks zero flux. Note that, as the IOP is varied, we also vary the capillary pressure $p_{\mathrm{CC}}=\mathrm{IOP}+5$ mm Hg and apical RPE pressure $p_{R}=\mathrm{IOP}$.

Various explanations have been proposed to explain the insensitivity of the flow to the IOP,^46^ which involve both the trans-scleral and uveo-vortex flows. One possibility is the collapsibility of the fluid domain: the collapse of the SCS and/or ciliary muscle will increase the hydraulic resistance, thereby impacting the change in flow rate,^46^ which would affect the trans-scleral component of the unconventional flow. Another plausible mechanism is that the pressure in the CC mirrors the IOP,^47^ meaning that, as the IOP varies, there is little change in the difference in hydrostatic plus oncotic pressure between the IF and CC, and, because this process drives the uveo-vortex flow, this flow also does not increase.

There are numerous theoretical models in the literature that have been developed successfully to describe aqueous production,^48^^,^^49^ flow,^50^^–^^53^ and drainage,^54^ and fluid flows more generally in the eye.^55^^,^^56^ However, the mechanics of the unconventional outflow pathway represents an under-studied aspect of ocular physiology, and there remains a notable absence of mathematical models in this area. In this article, we develop a novel mathematical model of this pathway, which allows us to test rigorously the mechanisms described above and theoretically assess the effectiveness of potential pharmacological therapies.

## Mathematical Model

In this section, we give an overview of the mathematical model for a general audience. Full details of the mathematical model and its derivation can be found in the Supplementary Material, and the section Details of Mathematical Model in the Appendix contains a shorter summary of the same information, consisting of statements of the key modeling assumptions and the equations that are used in the model development, as well as the full governing equations that are used to obtain the results presented in this article.

### General Description of the Model

We model IF flow and transport of albumin in the choroidal tissue and the SCS, a potential space between the choroid and sclera, which is illustrated in Figure 2. IF, modeled as a Newtonian incompressible fluid with dynamic viscosity µ and uniform absolute temperature *T*, flows posteriorly from the anterior chamber, where it has hydrostatic pressure IOP, through the iris root and ciliary muscle and into the choroidal tissue and SCS. From there, it flows out of the eye through the sclera or via the CC. Albumin flows into the choroidal tissue and SCS through the inlet at the anterior choroid and leaves via advection across the sclera. There is also exchange with the CC via both advection and diffusion.

We treat the choroidal tissue as a perfused porous layer of tissue, permeable to IF, with (Darcy) permeability $k_{C}$ (proportional to the hydraulic conductivity). Albumin diffuses in this tissue with the diffusion coefficient *D*, and is advected with the IF flow. The choroid has a prescribed thickness, $h_{C}$, which varies from anterior to posterior, and $h_{C0}$ is the average thickness.

The SCS is a potential space that exists between the choroid and sclera, which, although narrow in comparison with both of these tissues, represents a relatively low resistance pathway for IF flow.^57^^–^^59^ We assume (for simplicity and modeling expedience) that this space maintains a uniform prescribed thickness, $h_{P0}$, under physiological conditions. It is expected that the hydrostatic pressure in the SCS will change from its physiological value during nonphysiological conditions, such as changes in the IOP or after pharmacological treatment. It is straightforward to separate the sclera and the choroid during surgery, and, furthermore, the observations of Croft et al.^60^ show choroidal movement of between 0.1 and 1.0 mm parallel to the sclera during accommodation, both of which suggest there is little or no adhesion between the choroid and sclera. For simplicity in this work, we assume there is no adhesion between these surfaces. Hence, any variation in IF pressure in the SCS or choroidal tissue could cause the choroidal tissue to deform (the scleral tissue is much stiffer), altering the thickness of the SCS.

**Figure A1.**
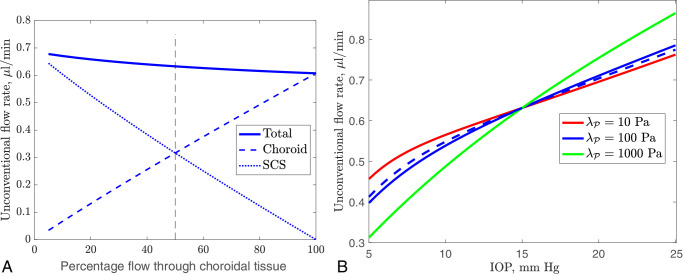
(**A**) Flow rate through the inlet as the proportion of flow through the choroidal tissue is varied. Instead of requiring that the flow rates through the choroidal tissue and SCS are equal at the inlet, we demand that the flow rate through the choroidal tissue is a fixed percentage of the total unconventional flow rate. For each value of this percentage, we calculate values of *R*_*icm*_, *k*_C_ and $h_{P0}$ that also fix the correct pressures at the inlet and posterior pole (more details in text). We show the flow rates through the choroidal tissue and SCS as well as the total unconventional flow rate. (**B**) Dependence of the unconventional flow rate on the IOP for three different values of $\lambda_{P}$ (legend, with $\lambda_{P}=100$ Pa (blue curve) being the baseline physiological value). The pressures $p_{\mathrm{CC}}=\mathrm{IOP}+5$ mm Hg and $p_{R}=\mathrm{IOP}$ vary with the IOP. The *blue dashed line* shows the case $p_{R}=\mathrm{IOP}-0.5$ mm Hg (with $p_{\mathrm{CC}}=\mathrm{IOP}+5$ mm Hg, $\lambda_{P}=100$ Pa).

As such, we assume that the actual thickness of the SCS in nonphysiological conditions, $h_{P}$, is dependent on the departure from the physiological value of the difference between the pressure in the SCS itself and that on the apical RPE, $p_{R}$ (see Equation (3) in the Appendix for a precise definition), and we assume $p_{R}$ equals the IOP (but interrogate the effect of lower values in Fig. A1B in the Appendix). If this pressure difference is positive and large compared with $\lambda_{P}$ (a parameter characterizing the elastic stiffness of the SCS), such as might occur during conditions of raised IOP, we model the thickness of the SCS as approximately linearly dependent on this pressure difference. To account for small and/or negative pressure differences (as is expected in normal physiological conditions), we modify the dependence to ensure that the SCS never fully closes. With this approach the SCS can, in principle, become arbitrarily narrow, and one could apply a suitable threshold to demarcate regions in which the SCS can be considered to be collapsed (an extension of this approach could be used to facilitate understanding of experimental observations^61^). Further details on our choice of constitutive law for the mechanical response of the SCS can be found in the Supplementary Material.

**Figure 6.**
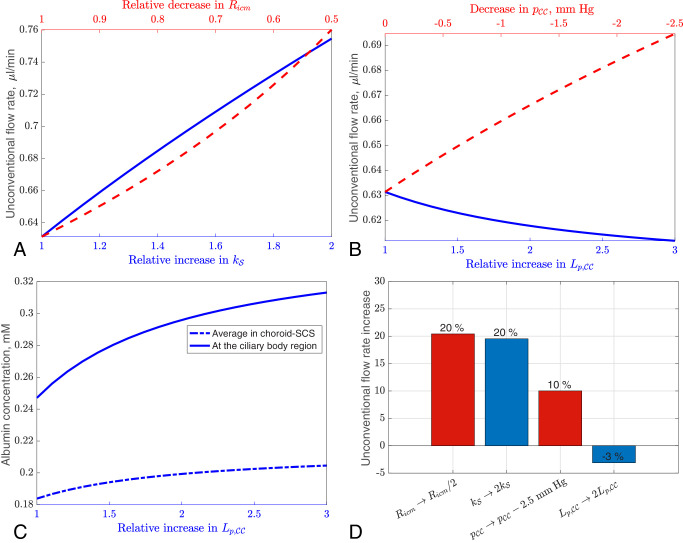
Parts (**A**) and (**B**) investigate four possible mechanisms of unconventional flow increase with the use of PGF_2α_ analogues; (**C**) shows albumin concentration in one of the cases (increasing $L_{p,\mathrm{CC}}$); (**D**) shows a summary of the four mechanisms. In all figures, we consider a departure from the reference physiological case (which is shown on the left sides of **A**–**C**), that is, the SCS height is allowed to deform. (**A**) Top axis (*red dashed*): Increase in unconventional flow rate with decreasing iris root resistance, *R*_*icm*_. The *x*-axis shows the factor by which *R*_*icm*_ is reduced, with 1 being the reference physiological case and 0.5 corresponding with the reduction of *R*_*icm*_ by a factor of 2. Bottom axis (*blue solid*): Unconventional flow rate increase with increasing scleral hydraulic conductivity, $k_{S}$. The *x*-axis is the factor by which $k_{S}$ is increased. (**B**) Top axis (*red dashed*): Unconventional flow rate increase as pressure in the capillaries, $p_{\mathrm{CC}}$, is reduced (extreme left is the reference physiological case, $p_{\mathrm{CC}}=\mathrm{IOP}+5$ mm Hg; extreme right is $p_{\mathrm{CC}}=\mathrm{IOP}+2.5$ mm Hg). Bottom axis (*blue solid*): Unconventional flow rate increase with increasing hydraulic conductance of the vessel walls, $L_{p,\mathrm{CC}}$. The *x*-axis is the factor by which we increase $L_{p,\mathrm{CC}}$ from the reference physiological case. (**C**) Albumin concentration with increasing hydraulic conductance (*x*-axis is the same as the bottom one in **B**). The *solid line* is the albumin concentration at the inlet and the punctured line is the spatially averaged albumin concentration in the choroid–SCS. (**D**) Summary of all four mechanisms of relative increase of the flow rate: decreasing *R*_*icm*_ by a factor of 2, doubling $k_{S}$, reducing $p_{\mathrm{CC}}$ by 2.5 mm Hg and doubling $L_{p,\mathrm{CC}}$. The colors of the bars correspond with the colors of the lines in **A** and **B**.

The scleral surface of the SCS exhibits both cellular components and extracellular matrix.^62^ However, it is not clear to what extent this structure inhibits the IF flow along this space, and so, for this reason, we adopt two approaches: in the main text we assume that IF can flow freely within the SCS, whereas in the Supplementary Material we also consider the case of the IF flowing in the gaps between material present in the space (flow through a porous medium). We find that the predictions of the model are qualitatively insensitive to this choice. We assume that the IF does not slip at the scleral and choroidal bounding surfaces of the SCS; the use of a more general condition is discussed in the Supplementary Material, where we show that the corresponding correction to our results would be very small.

The inlet of the model incorporates the posterior end of the ciliary body. We model the short region (approximately 3 mm) of tissue from the anterior chamber to the inlet, which comprises the iris root and ciliary body, as flow through a resistor with fixed resistance, *R*_*icm*_, driven by a pressure difference. Thus, the hydrostatic pressure difference between the IOP in the anterior chamber and that at the inlet of the model (lower) equals *R*_*icm*_ multiplied by the volume flow rate. Albumin in the ciliary body region is assumed to be well-mixed and we prescribe zero diffusive flux at the inlet.

The inner surface of the choroid is bounded by the RPE, across which the IF is pumped at a prescribed rate, $q_{R}$. We assume there is no transport of albumin across the RPE, owing to the presence of tight junctions connecting its cells.

We model the sclera as a rigid layer with thickness, $h_{S}$. The inner surface of the sclera is assumed to be spherical with radius *R*_0_, and the region under consideration in the mathematical model spans up to an angle θ_0_ from the posterior, corresponding to a distance of approximately 28.1 mm. On its outer surface, we assume a uniform prescribed orbital pressure, $p_{O}$, and albumin concentration, $c_{O}$. IF transport across the sclera is modeled using Starling’s law on the assumption that albumin is the only solute contributing to the local osmotic pressure difference between the IF in the choroidal tissue and that in the orbit. The hydraulic conductance is $k_{S}/\left(h_{S}\mu \right)$, where $k_{S}$ is the (Darcy) permeability of the scleral tissue, osmotic reflection coefficient is $\sigma_{S}$, and ideal gas constant is *R*_*g*_. Albumin transport across the sclera is modeled by the Patlak equation^63^ with permeability coefficient $\beta_{S}$.

Within the choroidal tissue, IF and albumin pass to and from the fenestrated CC. We describe the CC as uniformly distributed within the choroidal tissue, with surface area per unit volume of tissue $S_{\mathrm{CC}}$, uniform hydrostatic pressure $p_{\mathrm{CC}}$ and albumin concentration $c_{\mathrm{CC}}$. The hydrostatic pressure in the CC is assumed to be uniformly $p_{\mathrm{CC}}=\mathrm{IOP}+5$ mm Hg (and accordingly varies with the IOP, see also the section ‘Pressures’ in the Appendix).^47^ IF transport across the CC is modeled by Starling’s equation, again on the assumption that the local osmotic pressure difference only depends on differences in albumin concentration between plasma and IF. In this case, the hydrostatic pressure difference tends to force fluid out of the CC, while the osmotic pressure difference favours absorption into the CC. The hydraulic conductance is $L_{p,\mathrm{CC}}$ and the reflection coefficient is $\sigma_{\mathrm{CC}}$. Albumin is primarily transported by transcytosis,^20^ which for simplicity we model as a diffusion-like term with albumin conductance, $P_{d,\mathrm{CC}}$. The reflection coefficient $\sigma_{\mathrm{CC}}$ is near unity^64^; therefore, there is also little advective transport of albumin into the CC.

The unconventional flow rate predicted by the model is defined as the total rate of IF flow through the inlet of the model.

### Simplification of the Model

We focus on the transport along a fixed anterior–posterior slice bisecting the eye, and we assume all variables are independent of their circumferential position around the eye (Fig. 2). Choroidal thickness is approximately 2% of its anterior–posterior length, with the thickness of the SCS being much smaller. As such, it is appropriate to assume variations in all variables across the thickness of the SCS are much smaller than those along the length (∼28.1 mm) of the SCS. For a given distance posteriorly, it emerges that the IF pressures in the choroidal tissue and SCS vary negligibly in the direction perpendicular to the sclera, and so, by continuity, these are equal, meaning that we can work in terms of a single pressure variable, *p*, in both spaces, which depends only on the distance from the inlet. Similarly, a single albumin concentration, *c*, may be defined in both spaces. After simplification, the resulting system of equations, consisting of four coupled ordinary differential equations, governs the IF pressure, *p*, IF flow rate, *Q* (µL/min), albumin concentration, *c*, and albumin flow rate, *J* (mol/s). These are stated in Equations (19)–(22) in the Appendix, with boundary conditions below. We solve them using MATLAB R2024a (Mathworks), using the bvp4c solver, and present the results herein.

### Parameter Values

The model developed here is dependent on 28 parameters, whose typical physiological values are listed in Table 1.

**Table 1.** Baseline Physiological Values of the Model Parameters

Parameter Value	Description
IF	
μ = 0.7 · 10^−3^ Pa s	Dynamic viscosity of IF (equal to that of water at 37°C)
*T* = 37 + 273 K	Absolute temperature
IOP = 15 mm Hg	Physiological intraocular pressure
Choroidal tissue	
$k_{C}=2000k_{S}{\;}^{**}$	Darcy permeability of choroidal tissue, see section Fluid
*D* = 61 · 10^−12^ m^2^s^−1^	Albumin diffusion coefficient^67^^,^^68^
$h_{C}=\left(7.77\theta^{3}-45.4\theta^{2}-57.4\theta +397\right)$ µm	Thickness profile (θ is the angle in radians subtended at the center of the eye between the point and the posterior pole),^69^ see section Geometry
$h_{C0}=266$ µm	Average choroidal thickness
SCS	
$h_{P0}=2.4$ µm **	Thickness of SCS in physiological conditions, see section Fluid
$p_{R}=\text{IOP}$ *	Pressure on the apical RPE, see section Pressures
$\lambda_{P}=100$ Pa **	Elasticity parameter, see section Pressures
Inlet	
*R*_*icm*_ = 1.27 mm Hg/(µL/min) **	Resistance of iris root and ciliary muscle, see section Fluid
RPE	
$q_{R}=3\cdot 10^{-8}$ m/s *	Flow across the RPE,^70^^,^^71^ see section Fluid
Sclera	
$h_{S}=0.5$ mm *	Thickness of the sclera,^72^ see section Geometry
*R*_0_ = 1.15 · 10^−2^ m	Radius of the inner scleral surface,^73^ see section Geometry
θ_0_ = 140°	Angle subtended at the center of the eye between the anterior limit of choroid and the posterior pole of eye,^69^ see Figure 2 and section Geometry
$p_{O}=3$ mm Hg *	Orbital pressure,^74^ see section Pressures
$c_{O}=0.19c_{\mathrm{CC}}$ *	Albumin concentration in the orbit,^19^ see section Albumin
$k_{S}=5.85\cdot 10^{-18}$ m^2^	Darcy permeability of the scleral tissue,^75^ see section Fluid
$\sigma_{S}=0.38$	Albumin reflection coefficient of the sclera,^75^ see section Albumin
*R*_*g*_ = 8.314 J/(mol °K)	Ideal gas constant
$\beta_{S}=8.3\cdot 10^{-9}$ m/s	Albumin conductance of the sclera,^75^ see section Albumin
CC	
$S_{\mathrm{CC}}=12,000$ m^−1^ *	Surface area of the CC per unit volume of choroid, see section Geometry
$p_{\mathrm{CC}}=\text{IOP}+5$ mm Hg *	Hydrostatic pressure in the CC,^47^ see section Pressures
$c_{\mathrm{CC}}=0.66$ mM	Albumin concentration in the blood vessels,^76^ see section Albumin
$L_{p,\mathrm{CC}}=8\cdot 10^{-11}$ m/s/Pa **	Hydraulic conductance of the CC,^64^ see section Fluid
$\sigma_{\mathrm{CC}}=0.95$ *	Albumin reflection coefficient of the CC,^64^ see section Albumin
$P_{d,\mathrm{CC}}=8\cdot 10^{-10}$ m/s **	Albumin conductance of the CC,^77^ see section Albumin

Parameters whose values have a significant degree of uncertainty are denoted with *, whereas those for which we have no, or very little, information with **. For more details on the choices of parameter values, please see the section ‘Estimation of Parameter Values’ in the Appendix (titles of subsections are given).

Three parameter values for which we have no information are the hydraulic resistance of the iris root and ciliary muscle, *R*_*icm*_, the (Darcy) permeability of the choroidal tissue, $k_{C}$, and the physiological thickness of the SCS, $h_{P0}$. To set these three, we assume that the pressures at the inlet and posterior pole match those in the literature,^58^ and that the flow rates of IF through the choroidal tissue and the SCS are equal at the inlet (see also the section ‘Fluid’ in the Appendix). The latter choice is explored in Figure A1A.

Furthermore, we could not find any value for the elasticity parameter quantifying changes in the thickness of the SCS in nonphysiological conditions, $\lambda_{P}$. We choose a value for this such that there is significant expansion or collapse of the SCS over the range of IOP considered in this article (5–25 mm Hg), which in turn allows us to assess whether collapsibility of the SCS is a possible mechanism explaining the observed pressure insensitivity of the unconventional flow to the IOP.

Moreover, there are several other values listed in Table 1 that were either obtained indirectly or for which values reported in the literature vary significantly. Details of all of these choices are given in the section ‘Estimation of Parameter Values’ in the Appendix (see references in Table 1), and we have also indicated those parameter values whose values have a degree of uncertainty (*) or are very uncertain (**).

### Sensitivity Analysis

To quantify the effect of the uncertainty in the parameter values on the model output, we perform a global sensitivity analysis using the extended Fourier amplitude sensitivity test, eFAST.^65^ eFAST is a variance-based approach that dissects output variance in the predictions using a spectral analysis to calculate sensitivity indices (SI) for each parameter under consideration. These indices measure to what degree each parameter value affects the model output. The first-order SI describes the isolated effect of each parameter, because it quantifies the reduction in variance of the model output if that parameter were kept fixed. The total SI captures both this first-order effect and the interaction of the given parameter with the other parameters, because it measures the expected variance that would remain if all but the given parameter were fixed. The sensitivity analysis was carried out using the MATLAB codes developed by the group of Dr Kirschner.^66^ More details on the implementation are reported in the Appendix.

## Results

We start by presenting the model results with the baseline parameter set listed in Table 1, which we refer to as the reference physiological case; we follow this with the results of the sensitivity analysis of this solution. We will then discuss nonphysiological cases, to examine the response of the unconventional flow to changing conditions: first, we impose a change in the IOP to determine how this influences the unconventional flow; second, we change the model parameters to investigate the postulated action of PGF_2α_ analogues, as well as comparing alternative explanations of the action of these drugs.

### Reference Physiological Case

We use the parameter values listed in Table 1 and solve the governing equations to obtain the IF pressure and flow and the albumin concentration, with results shown in Figure 3.

The green curve in Figure 3A shows the pressure distribution in the IF, *p*, along the tissue of the choroid and the SCS, which we hereinafter refer to as the choroid–SCS, and, for comparison, the uniform pressures in the CC, $p_{\mathrm{CC}}$ (red), the orbit, $p_{O}$ (cyan), and the IOP (magenta) are also shown. As prescribed by our choice of model parameters (*R*_*icm*_, $k_{C}$ and $h_{P0}$, see p. 33), the pressure is 14.2 mm Hg at the inlet (posterior ciliary body/anterior choroid, slightly below the IOP of 15 mm Hg), and 11.3 mm Hg at the posterior pole. Both values are well above the orbital pressure of 3 mm Hg owing to the high hydraulic resistance of the sclera. Our results show that the pressure rapidly decreases over the peripheral choroid–SCS near to the inlet, and has a near uniform pressure distribution in the posterior part of the choroid–SCS.

Figure 3B shows albumin concentrations, with the green curve showing the concentration in the choroid–SCS, *c*, with the uniform albumin concentrations imposed in the CC, $c_{\mathrm{CC}}$ (red), and orbit, $c_{O}$ (cyan), plotted for comparison. The concentration in the choroid–SCS is everywhere higher than the orbital concentration and lower than that in the CC, and it exhibits only a slight decrease over the peripheral choroid–SCS, with a near uniform concentration in the posterior. The overall mean concentration is 28.8% of that in plasma (0.66 mM), (Table 1), which is within the range of 10% to 35% reported in the literature.^18^^,^^19^

In Figures 3C and 3D, we show the IF and albumin flow rates, respectively, along the choroid–SCS. The total IF flow rate (purple solid line), which is the sum of flows through the choroidal tissue (dashed line) and the SCS (dotted line), decreases toward the posterior pole. At the inlet, the overall IF flow rate in the choroidal tissue and that in the SCS are equal by our choice of parameters, and the unconventional flow rate equals the sum of these two rates. The value predicted by the model has the right order of magnitude: assuming an aqueous production rate of 2.5 µL/min, the model predicts that the unconventional pathway accounts for approximately 25% of the total outflow.^9^ The albumin flow rate (Fig. 3D) follows the same pattern as that of the IF, decreasing as it progresses through the domain.

Figure 3E details the IF exchange between the choroid–SCS and the surrounding compartments, specifically the CC (red), sclera (blue), and RPE (yellow). Positive values indicate flows into the choroid–SCS, whereas negative values indicate flows out. Table 2 gives the corresponding flow rates of IF between the compartments. IF enters the choroid–SCS from both the inlet (0.63 µL/min, 19%) and the RPE (2.64 µL/min, 81%), and leaves through the sclera (1.67 µL/min, 51%); in addition, there is a net flow into the CC of 1.67 µL/min (49%).

**Table 2.** IF and Albumin Flow Rates Predicted by the Model Between the Choroid–SCS and Other Compartments (Negative Values Indicate a Flow Out of the Choroid–SCS)

Compartment	IF Flow Rate (µL/min)	Albumin Flow Rate (10^−12^ mol/s)
From inlet	0.63	2.60
Into/from CC	−1.61	1.06
		(−0.57 enters CC by advection
		1.63 leaves CC by transcytosis)
Through the sclera	−1.66	−3.66
From the RPE	2.64	0

Figure 3F and Table 2 show the corresponding exchanges for albumin (there is no albumin flow across the RPE, because it is assumed to be impermeable to albumin). Thus, albumin enters the choroid–SCS by a combination of flow through the inlet (61%) and transcytosis from the CC (39%), and it leaves the choroid–SCS via advection with the IF flow into the CC (14%) and through the sclera (86%). We note that the rate of albumin loss from the CC is 10.6 · 10^−13^ mol/s per eye, in good agreement with measurements of Bill in a rabbit eye.^78^ Also, note that the crystalloid component of IF flows into the CC, but the net flow of albumin is out of the CC.

### Sensitivity Analysis

As mentioned, there is a degree of uncertainty in some of the parameter values, and to understand the potential effect of this on the model results, we perform a global sensitivity analysis, whose output is two SI. These are plotted in Figure 4, which shows the effect of the choice of parameter values upon the resulting unconventional flow rate (A) and the average albumin concentration (B). The heights of the bars indicate the first-order (blue) and total-order (red) SI for each model parameter. In each case, the values of the first and total SI have similar magnitudes, suggesting that there is little interaction between the model parameters. For those parameter values with the highest SIs (for which we assume that a change in their value has the most significant effect on the output), the small black arrows show the direction of influence; an upward-pointing arrow indicates that increasing the parameter value increases the corresponding model output.

In Figure 4A, we see that the IF flow rate is most sensitive to the thickness of the SCS, $h_{P0}$. This is because the resistance of the SCS decreases as 1/$h_{P0}^{3}$ when this thickness is increased, resulting in higher flow rates. Scleral (Darcy) permeability, $k_{S}$, is another important parameter: increasing $k_{S}$ results in a greater trans-scleral outflow, and thus in a larger unconventional flow rate. Pressure in the CC, $p_{\mathrm{CC}}$, also plays an important role; increasing $p_{\mathrm{CC}}$ reduces the uveo-vortex flow into the CC, and thus the unconventional flow rate.

Figure 4B shows that the average albumin concentration in the choroidal tissue is most sensitive to the pressure in the CC, $p_{\mathrm{CC}}$; increasing this value results in decreasing albumin concentration. Other important parameters are the albumin conductance of the CC, $P_{d,\mathrm{CC}}$, and the flow across the RPE, $q_{R}$. Increasing $q_{R}$ results in a decrease of albumin in the tissue, as the fluid that comes from the RPE is free of albumin. Increasing $P_{d,\mathrm{CC}}$ increases the release of albumin from the CC and thus its concentration in the choroid–SCS. In comparison with the key parameters mentioned here, the remaining parameters have little influence on IF flow or albumin concentration.

### Dependence of the Unconventional Flow on the IOP

In Figure 5A, we report with dots the experimental measurements by Bill^5^ on living monkeys for the conventional (green) and unconventional (black) routes (with dashed straight lines drawn through these points for clarity). Our predictions of unconventional outflow in the baseline case are also reported (thick blue curve). The model predicts that unconventional flow rate increases in a nearly linear fashion with the IOP, and that the relationship is slightly nonlinear at low values of the IOP, with a steeper gradient at low IOP.

Our model includes various mechanisms that control the response of the model to a change of IOP. In particular, (1) osmotic pressure differences owing to local differences in albumin concentration between the plasma in the CC and the IF in the choroidal tissue, which affects fluid exchange across vessel walls, and osmotic pressure differences across the sclera, which affects the trans-scleral flow, and (2) the existence of the collapsible SCS. A strength of mathematical modeling is that these effects can be switched on or off individually, and this is done in Figure 5B, as indicated in the table in the legend.

If we remove the effect of differences in albumin concentration, both across the vessel walls of the CC and across the sclera, on the IF flow (which we do by setting the reflection coefficients for the CC and sclera ($\sigma_{\mathrm{CC}}$ and $\sigma_{S}$, respectively) to zero (see dashed lines in Fig. 5B), the direction of the flow is toward the iris root, opposite to what is observed physiologically. This is because there is no uveo-vortex flow in this case, because the hydrostatic pressure is higher in the CC than it is in the choroidal interstitial tissue. Because the resistance to outflow through the sclera is much higher than the resistance to flow of the choroid–SCS, the IF partly escapes through the inlet toward the ciliary body. We note that this direction of the unconventional flow is due to the choice of parameters in Table 1, and it can be modified by lowering the resistance of the sclera, for instance. If the SCS and oncotic pressure are both absent (red dashed line), the model predicts a perfectly linear dependence of the IF flow rate on the IOP. With the SCS present (blue dashed line), the magnitude of the flow is greater, because the combined resistance of the choroid–SCS is lower than that of the choroidal tissue alone. The scenario changes significantly when the effect of osmotic pressure differences are included (solid curves) and the IF flows posteriorly through the inlet in the correct direction.

We note that the predicted values of the unconventional flow rates with no SCS (red curve, errors of 12% and 3% at 11 and 22 mm Hg, respectively) fit the absolute values given by Bill’s data slightly better than those with the SCS present (blue curve, errors of 27% and 18%). However, the slope of our predicted values with increasing IOP is noticeably closer to that of Bill’s data in the case with the SCS present (between 11 and 22 mm Hg the slope of Bill’s data is 0.0173 µL/min/mm Hg, and the corresponding slopes of the blue (with SCS) and red (without SCS) curves are 0.0165 and 0.202 µL/min/mm Hg, respectively). Given that Bill’s data were measured in monkeys (which have a very different baseline IOP, 11 vs. 15 mm Hg in humans), we feel that this slope is a more robust comparator between the approaches, because it quantifies the unconventional flow rate in terms of an IOP increase from a baseline value (which could vary from individual to individual within a species).

### The Use of PGF_2α_ Analogues

We tested various possible mechanisms of unconventional flow increase when PGF_2α_ analogues are used. The mechanisms we will consider include: lowering the resistance of the iris root and ciliary muscle, *R*_*icm*_, increasing the scleral hydraulic conductivity, $k_{S}$, lowering the CC pressure, $p_{\mathrm{CC}}$, and increasing the capillary hydraulic conductance, $L_{p,\mathrm{CC}}$. In all cases, we consider the departure from the reference physiological case, while keeping the IOP fixed at 15 mm Hg.

The standard explanation for the increase in the unconventional flow rate when using PGF_2α_ analogues is that there is a decrease in the resistance to the flow across the ciliary muscular tissue, corresponding with a drop in the parameter *R*_*icm*_ in our model.^29^^,^^30^ We show this effect in Figure 6A (red curve and top axis). As *R*_*icm*_ decreases, the flow rate increases, by up to 20% when *R*_*icm*_ is decreased by a factor of 2. However, given the large increase in unconventional flow that is required for a significant decrease in the IOP, this increase is unlikely to be sufficient to explain the action of the drug. Accordingly, we seek other contributory factors to explain the action of PGF_2α_ analogues.

A second possible explanation is that there is an increase in scleral hydraulic conductivity, $k_{S}$. The dependence of the unconventional flow rate on $k_{S}$ is shown in Figure 6A (blue curve and bottom axis), indicating a 20% increase in the flow rate if the hydraulic conductivity is doubled.

Next, we explore the effects of venodilation, which is an alternative explanation for the increase in unconventional flow, in Figure 6B. We show the increase of the unconventional outflow rate when CC hydrostatic pressure is reduced (red dashed and top axis). The pressure is reduced from its physiological value of IOP + 5 mm Hg (left) to IOP + 2.5 mm Hg (right), which increases the unconventional flow by only about 10%. We also show that the flow rate decreases with increasing hydraulic conductance of the CC, $L_{p,\mathrm{CC}}$ (blue curve and bottom axis). Increasing $L_{p,\mathrm{CC}}$ twofold decreases the flow rate by about 2%.

A possible explanation for the reported appearance of fluid spaces in the ciliary muscular tissue is that an increase of albumin in the uveal interstitial tissue results in tissue edema. In Figure 6C, we show the corresponding concentration of albumin at both the inlet and the average over the choroid–SCS domain as hydraulic conductance of the CC, $L_{p,\mathrm{CC}}$, increases. The concentration increases with $L_{p,\mathrm{CC}}$, because the exchange of albumin between the IF and blood is enhanced, and this effect is more pronounced at the inlet (20% for a two-fold increase and 27% for a three-fold increase) than the average over the choroid–SCS domain (9% and 11%, respectively). This is because the trans-scleral flow removes the excess albumin along the length of the choroid–SCS, but this effect is cumulative from the inlet.

This pronounced increase in albumin concentration explains the small decrease in unconventional flow that is observed when increasing the hydraulic conductance, $L_{p,\mathrm{CC}}$ (blue curve in Fig. 6B), because the increased albumin concentration increases the osmotic pressure in the choroidal tissue, reducing the flow from the choroidal tissue into the CC. This is despite the fact that resistance to flow of the uveo-vortex pathway is reduced.

Figure 6D summarizes the mechanisms discussed in this Section by showing the relative increase of the unconventional flow due to the four different effects considered in Figures 6A, 6B.

## Discussion and Conclusions

In this article, we have presented a new mechanistic mathematical model of the unconventional flow pathway in the human eye. This model encodes the known anatomical features of unconventional flow, as described in the Section Mathematical Model, and it is encouraging that it succeeds in capturing some of the key physiological features.

Like many mathematical models of biological systems, our model depends on several parameters, some of which are uncertain. To understand the impact of our choice of parameters on the model outputs, we conducted a global sensitivity analysis. We found that, although the quantitative predictions are influenced by the specific parameter set used, the qualitative behaviour remains consistent. Importantly, the model offers a plausible explanation for the physics underlying the unconventional flow pathway in terms of the mechanisms of insensitivity of the flow rate to the IOP and the mechanisms of the action of PGF_2α_ analogues.

### Pressure Insensitivity

A key feature of the unconventional flow is that it is relatively insensitive to the IOP,^79^ and our model similarly predicts only a weak dependency on the IOP, as well as predicting a flow of the correct magnitude and direction. Inspection of Figure 5A indicates that the relationship between the unconventional flow rate and the IOP is fairly close to linear (though the graph does show a slight curvature); the conventional flow has a linear dependence. However, the unconventional flow is only weakly dependent on the IOP, in the sense that increasing the IOP from 11 to 22 mm Hg induces a relatively small percentage increase on the unconventional flow (∼32%). This is not the case for the conventional flow, which increases in proportion to the difference from episcleral venous pressure (corresponding to a 450% increase over the same range). This large difference is a consequence of the fact that the IOP for which the unconventional flow rate is zero is less than 5 mm Hg, whereas the corresponding pressure where the conventional flow rate is zero is the episcleral venous pressure of 8.4 mm Hg. This significant difference in the pressures where the two flow rates are zero, rather than any nonlinearity in the flow rate–IOP relationship, is the primary reason why the observed change in the unconventional flow rate when the IOP is changed is so small compared with the corresponding change in the conventional flow rate. This agrees with and explains the experimental observations of Bill.^5^

To explain the pressure insensitivity mechanically, we note that there are two features of the model that, in combination, decrease the dependence of the flow rate on the IOP, as follows. First, the uveo-vortex flow (the flow into the venous capillaries) is affected by the albumin concentration in the IF, which is much lower than that in plasma, meaning the resulting osmotic pressure difference acts to absorb IF into the venous capillaries. Because the CC pressure is set at 5 mm Hg higher than the IOP, the hydrostatic pressure difference between the IF and blood does not change with the IOP, because the IOP is varied, and thus the difference in both the hydrostatic and osmotic pressures between IF and blood do not depend strongly on the IOP, with the result that the uveo-vortex flow does not depend strongly on the IOP also.^80^

Second, the SCS is compressible, resulting in it having a variable hydraulic resistance. When the pressure in the SCS is low, the SCS compresses, increasing its resistance to IF flow and tending to decrease the flow. Conversely, the large hydrostatic pressure drop between the IOP and the relatively low SCS pressure drives increased IF flow. When the pressure in the SCS is high, the SCS expands, tending to increase flow, but there is a small pressure drop, which tends to decrease the flow. The result of these opposing mechanisms is that the flow rate in this space is relatively weakly dependent on the IOP, although our results suggest that this effect is relatively small.

In contrast, the other components governing the unconventional flow in the model, including the flow through the inlet resistor, choroidal tissue and the trans-scleral flow,^81^ are all dependent on the IOP. However, the relative insensitivity of the uveo-vortex flow on the IOP and the compressibility of the SCS in combination with the other components results in a reduced overall sensitivity of the unconventional flow rate to the IOP compared with that of the conventional flow rate, as observed in experiments and predicted by the model.

### IOP-Lowering Action of PGF_2α_ Analogues

This model sheds light on the mechanisms of action of PGF_2α_ analogues used to treat glaucoma, which work by increasing the unconventional outflow. Our model shows that decreasing the resistance of the iris root and ciliary muscle does have some effect on the unconventional flow rate, but this is not enough to explain the expected pressure drop fully, suggesting that additional mechanisms are required. These drugs are also reported to increase scleral hydraulic conductivity, and our model predicts a modest increase in unconventional flow under this condition.

We also tested two further possible mechanisms. If the choroidal vessels dilate, the CC pressure will be reduced, and, in these conditions, our results indicate a relatively small increase in the unconventional flow. Furthermore, increasing the hydraulic conductivity of the CC has little effect on the flow (it decreases very slightly).

It is likely that the observed IOP-lowering action of PGF_2α_ analogues is due to a combination of the first three mechanisms. We do note, however, that the current investigation is preliminary. In particular, we assumed a normal IOP of 15 mm Hg, but in reality the drug is usually given to patients with POAG, who have a much higher IOP. For these patients, the unconventional outflow would be a much higher proportion of total outflow (as the conventional outflow is impaired). Further research is required to understand fully the role of PGF_2α_ analogues in pathological conditions.

### Other Actions of PGF_2α_ Analogues

The observation that latanoprost causes choroidal thickening mentioned in the Introduction could be explained by a direct action of the drug, but our model gives a possible alternative explanation for this, as follows. Because MMPs are involved in the regulation of endothelial hydraulic conductance, it could be that they increase the hydraulic conductance of the CC walls sufficiently to raise the interstitial albumin level, particularly near the inlet (Fig. 6C), and such an increase is known to cause tissue edema. The observed appearance of fluid-filled spaces in the ciliary muscle following the use of PGF_2α_ analogues could be due to a similar mechanism.

It is also known that latanoprost stimulates the accumulation of tracer from the anterior chamber in the cervical lymph nodes,^82^ and that the pressure-lowering effects of latanoprost are compromised in patients who have had their cervical lymph nodes removed.^83^ We anticipate that the use of the drug would increase the trans-scleral albumin flux. For example, the percentage increases in the trans-scleral albumin flux corresponding with the conditions of the four bars in Figure 6D are, respectively, 17%, 40%, 34%, and 10%; these are greater increases even than the increases in IF flow, suggesting that the albumin flux could increase significantly when the drug is used. This process would result in increased orbital albumin, which enters the orbital lymphatics en route to the destination in the cervical lymph nodes. Failure to clear this washed-out albumin in patients with no cervical lymph nodes could result in increased albumin concentration in the choroid–SCS. High albumin concentrations in the choroid–SCS would reduce the uveo-vortex flow owing to the reduced oncotic pressure, and this could explain the poor results of latanoprost treatment in these patients.

### The Role of Lymphatics

Although not the original goal of the model, our model does provide some insight into the role of lymphatics. Our model shows that the IOP-dependent trans-scleral component of the unconventional flow effectively removes albumin from the choroid–SCS. In this way, it takes on the role that is undertaken by lymphatics in most other tissues (although there have been reports of lymphatics present in the ciliary body,^10^ but this has not been confirmed by others^84^). None have been reported in the choroid, and, although tracers from the anterior chamber have been recovered from cervical lymph nodes,^82^ this does not mean that the site of entry into the lymphatic system had to be in the eye. The data are consistent with the tracers entering the lymphatic system in the orbit or in the conjunctiva after exiting the eye.^46^ The fact that our model produces accurate predictions without incorporating lymphatics is in line with the consensus view that the choroid lacks a lymphatic supply.

### Future Potential

There are several effects that could be worth further investigation, which we mention in this section. First, as mentioned in the Introduction, a number of other drugs can affect the unconventional pathway, and extensions of this work could have implications for our understanding of their actions. Second, increasing vascular hydraulic conductance or decreasing venous pressure is expected to enhance the unconventional flow. There are drugs that can do this, and it would be worth exploring their use a potential alternative glaucoma treatments.

Third, the unconventional flow has been shown to carry particulate tracers from the SCS to the posterior retina,^85^ raising the possibility of a noninvasive route for drug delivery to the back of the eye from the anterior chamber. Any agent that can enter the anterior chamber can potentially be delivered to the posterior regions of the retina or the choroid by the unconventional flow. Although this is currently considered unlikely,^86^ it is worth further consideration, and using drugs such as those mentioned in this article to enhance the unconventional flow would also enhance the drug delivery along this route. A model based on the one presented herein could be used as a starting point to understand the distribution of the agent.

Fourth, the complex physiology represented by the resistor at the inlet of the model is highly simplified, and could be explored in an extension of the model. A fraction of the unconventional flow will leave via the vessels in the ciliary body, as well as across the sclera in this region, and we do not account for this.

Fifth, the pressure at the inlet might not be uniform as there could be hotspots of outflow in the anterior chamber around the collector channels, which might perturb the pressure field there.

Sixth, in reality, the CC has a spatial distribution of pressures, with higher pressures near the arterioles supplying blood to the CC and lower pressures near the venules that drain it, which are spaced on a typical lengthscale of 400 to 800 µm, with pressure variation between these points.^87^ Given that the vessels of the CC have large diameters of about 20 µm compared with typical capillary vessels, and that the pressure drop needed to drive channel flow scales as the third power of the channel width, we would expect the pressure variation within the CC to be small in comparison with that in other capillary beds.

Seventh, saccades of the eye would affect the fluid pressures therein due to the acceleration, and we have not accounted for these in our model.

## Supplementary Material

Supplement 1

## Appendix

### Details of Mathematical Model

#### Governing Equations for the IF Flow

We consider an axisymmetric model of the choroidal tissue, SCS and sclera. We assume the inner surface of the sclera is perfectly spherical with radius *R*_0_, and work in spherical coordinates (*r*, θ, ϕ) centered on the sphere with θ = 0 in the direction of the posterior pole of the eye. The thickness of the SCS and choroid are given by $h_{P}\left(\theta \right)$ and $h_{C}\left(\theta \right)$, respectively, with 0 ⩽ θ ⩽ θ_0_, where θ = θ_0_ gives the anterior limit of the choroid at the ciliary body region. As such, assuming axisymmetry, the SCS is defined over $R_{0}-h_{P}\left(\theta \right)<r<R_{0}$ and the choroid over $R_{0}-h_{P}\left(\theta \right)-h_{C}\left(\theta \right)<r<R_{0}-h_{P}\left(\theta \right)$. We assume that $\varepsilon_{C}=h_{C0}/R_{0}\ll 1$, $\varepsilon_{P}=h_{P0}/R_{0}\ll 1$, where $h_{C0}$ and $h_{P0}$ are typical values of $h_{C}$ and $h_{P}$, respectively. We model the flow of IF and albumin transport in both the choroidal tissue and in the SCS, assuming that the IF is incompressible and Newtonian. The trans-scleral flow is driven by the difference between the IOP at the iris root and the orbital pressure (outside the sclera). In addition, within the choroid, there is IF flow and mass transport between the CC and the tissue. For simplicity, we describe the CC as uniformly distributed, with uniform properties throughout the layer.

In the tissue of the choroid, we assume incompressible Darcy flow, with $u_{C}=\left(u_{Cr},u_{C\theta },0\right)$ being the Darcy velocity (flow per unit area):

(1) $$\nabla \cdot u_{C}=q_{C},\;u_{C}=-\frac{k_{C}}{\mu }\nabla p_{C},$$

where $k_{C}$ is the Darcy permeability of the tissue, µ is the dynamic viscosity of the IF, and $p_{C}$ is the IF pressure. Within the choroidal tissue, the volume of IF entering the domain from the blood vessels per unit time per unit volume of tissue is given by

(2) $$q_{C}=L_{\mathrm{CC}}\left(p_{\mathrm{CC}}-p_{C}-\sigma_{\mathrm{CC}}R_{g}T\left(c_{\mathrm{CC}}-c_{C}\right)\right).$$

Here, $L_{\mathrm{CC}}$ represents the conductance of the CC, given by $L_{\mathrm{CC}}=S_{\mathrm{CC}}L_{p,\mathrm{CC}}$, and $p_{\mathrm{CC}}$ denotes the prescribed local blood pressure within these vessels. The albumin concentrations in the choroidal tissue and CC are given by $c_{C}$ and $c_{\mathrm{CC}}$, respectively, with $c_{\mathrm{CC}}$ fixed. The symbol *R*_*g*_ denotes the universal gas constant, *T* denotes the absolute temperature and σ_*CC*_ is the osmotic reflection coefficient.

We assume the sclera is rigid, and the thickness of the SCS, $h_{P}$, is determined by the hydrostatic pressure difference between the pressure there, $p_{P}$, and the pressure on the apical RPE, $p_{R}$. We assume the constitutive law

(3) $$p_{P}=p_{R}+\lambda_{P}\left(\frac{h_{P}}{h_{P,\mathrm{nat}}}-{\left(\frac{h_{P}}{h_{P,\mathrm{nat}}}\right)}^{-n}\right),$$

in which we set the exponent *n* equal to unity, where $\lambda_{P}$ is the prescribed elastic stiffness. The variable $h_{P,\mathrm{nat}}$ is the thickness that the SCS would adopt if there were no difference between the IF pressure in the SCS and the pressure in the retina; note that this scenario is not physiological, and that we will calculate values of $h_{P,\mathrm{nat}}$ from the model. This law is similar to tube laws used to model arteries and veins ^89^^–^^92^ and the response is linearly elastic if $\left(p_{P}-p_{R}\right)/\lambda_{P}$ is large, but the space strongly resists collapse if $p_{P}$ is small, preventing complete closure.

In the SCS, flow is governed by the Stokes equations:

(4) $$\nabla \cdot u_{P}=0,\;\nabla p_{P}=\mu \nabla^{2}u_{P},$$

where $u_{P}=\left(u_{Pr},u_{P\theta },0\right)$ is IF velocity.

Treating the tissue of the sclera as a membrane, we assume that IF crosses owing to the hydrostatic and osmotic pressure differences across it:

(5) $$\begin{array}{ccc} & & \;{\hat{n}}_{P,S}\cdot u_{P}{|}_{r=R_{0}}=-u_{Pr}{|}_{r=R_{0}}=-q_{S}=-\frac{k_{S}}{\mu h_{S}}\\ & & \;\;\times \left(\left(p_{P}{|}_{r=R_{0}}-p_{O}\right)-\sigma_{S}R_{g}T\left(c_{P}{|}_{r=R_{0}}-c_{O}\right)\right), \end{array}$$

where ${\hat{n}}_{P,S}=-{\hat{e}}_{r}$ is the inward pointing unit normal vector, $q_{S}$ is the volume of IF crossing the sclera per unit surface area per unit time, $k_{S}$ is the Darcy permeability of the sclera, $h_{S}$ is the thickness of the sclera, $p_{O}$ and $c_{O}$ are the orbital pressure and albumin concentration, respectively, and $\sigma_{S}$ is the reflection coefficient. We assume that IF enters the choroidal tissue from across the RPE at a prescribed rate:

(6) $${\left.{\hat{n}}_{R,C}\cdot u_{C}\right|}_{r=R_{0}-h_{P}-h_{C}}=-q_{R},$$

where $q_{R}$ is the flow per unit surface area and ${\hat{n}}_{R,C}$ is the inward pointing unit normal vector at the RPE–choroid interface $r=R_{0}-h_{P}-h_{C}$. Note that the flow rate $q_{R}$ is assumed to be uniform and constant, because it is mainly driven by active ion transport^56^ and only weakly depends on the hydrostatic and oncotic pressure differences between the choroidal tissue and the subretinal space.^93^

At the interface between the choroid and SCS, $r=R_{0}-h_{P}$, we have continuity of normal stress. We assume the normal stresses of both the IF in the SCS and the fluid–porous medium complex are dominated by the IF hydrostatic pressures (this assumption will be checked a posteriori), giving the balance

(7) $${\left.p_{C}\right|}_{r=R_{0}-h_{P}}={\left.p_{P}\right|}_{r=R_{0}-h_{P}}\;.$$

Furthermore, mass conservation requires continuity of normal flux:

(8) $${\left.{\hat{n}}_{C,P}\cdot u_{C}\right|}_{r=R_{0}-h_{P}}={\left.{\hat{n}}_{C,P}\cdot u_{P}\right|}_{r=R_{0}-h_{P}}\;,$$

where ${\hat{n}}_{C,P}$ is the inward pointing unit normal vector at the choroid–SCS interface.

In the SCS, we need additional boundary conditions to model the physics of the tangential component of the flow, which are provided by the no-slip boundary condition:

(9) $${\left.{\hat{t}}_{P,S}\cdot u_{P}\right|}_{r=R_{0}}=0,\;{\left.{\hat{t}}_{C,P}\cdot u\right|}_{r=R_{0}-h_{P}}=0,$$

where ${\hat{t}}_{P,S}$ and ${\hat{t}}_{C,P}$ are the corresponding unit tangential vectors.

Finally, we assume that the tissue between the anterior chamber and anterior choroid (comprising the tissue of the iris root and ciliary muscle) has resistance to fluid flow *R*_*icm*_, which we assume is a constant parameter and independent of the pressure. This leads to the boundary condition

(10) $$\mathrm{IOP}-p=R_{icm}Q,$$

at θ = θ_0_, where *p* is the mean pressure in the choroid–SCS and *Q* is the volume flow through the tissue. Symmetry at the posterior pole θ = 0 requires

(11) $$u_{C}\cdot {\hat{e}}_{\theta }=u_{P}\cdot {\hat{e}}_{\theta }=0.$$

#### Governing Equations for Albumin Transport

We assume a dilute solution of albumin and use the steady diffusion–convection equation to model its transport:

(12) $$\nabla \cdot j_{C}=s_{C},\;j_{C}=u_{C}c_{C}-D_{C}\nabla c_{C}\;\text{in}\;\text{choroidal}\;\text{tissue},$$

(13) $$\nabla \cdot j_{P}=0,\;j_{P}=u_{P}c_{P}-D_{P}\nabla c_{P}\;\text{in}\;\text{SCS}.$$

In these equations, $j_{C}$ and $j_{P}$ are the fluxes of albumin in the choroidal tissue and SCS, respectively, $c_{C}$ and $c_{P}$ are concentrations in the choroidal tissue and SCS, respectively. $D_{C}$ and $D_{P}$ are the diffusion coefficients of albumin in the choroidal tissue and SCS, respectively; we assume $D_{C}=D_{P}=D$.

In the choroidal tissue, to model exchange of albumin with the vessels of the CC, we adapt equations based on those proposed by Kedem and Katchalsky.^94^ The net rate of albumin entering the choroidal tissue per unit volume from the blood vessels locally reads:

(14) $$\begin{array}{c} s_{C}=\beta_{\mathrm{CC}}\left(c_{\mathrm{CC}}-c_{C}\right)+\frac{c_{C}+c_{\mathrm{CC}}}{2}\left(1-\sigma_{\mathrm{CC}}\right)q_{C},\; \end{array}$$

where $\beta_{\mathrm{CC}}$ is the permeation coefficient of the CC per unit volume multiplied by *R*_*g*_*T*, which is given by $\beta_{\mathrm{CC}}=S_{\mathrm{CC}}P_{d,\mathrm{CC}}$, where $P_{d,\mathrm{CC}}$ is the albumin conductance. The first term is a simplified description of transcytosis plus any other diffusive processes present, while the second term accounts for small advective backflow of albumin into the CC.

We assume that a flux $s_{S}$ of albumin passes through the sclera (which has both advective and diffusive components and thus depends on the local fluid velocity and albumin concentrations) and that albumin is not allowed to pass through the RPE, giving the respective boundary conditions

(15) $$\;{\left.{\hat{n}}_{P,S}\cdot j_{P}\right|}_{r=R_{0}}=s_{S},\;{\left.{\hat{n}}_{R,C}\cdot j_{C}\right|}_{r=R_{0}-h_{P}-h_{C}}=0,$$

with ${\hat{n}}_{P,S}$, ${\hat{n}}_{R,C}$ the outward pointing unit normals. At the choroid–SCS interface, $r=R_{0}-h_{P}$, we have continuity of concentration and normal flux:

(16) $$\begin{array}{ccc} & & {\left.c_{C}\right|}_{r=R_{0}-h_{P}}={\left.c_{P}\right|}_{r=R_{0}-h_{P}}\;,\\ & & {\left.{\hat{n}}_{C,P}\cdot j_{C}\right|}_{r=R_{0}-h_{P}}={\left.{\hat{n}}_{C,P}\cdot j_{P}\right|}_{r=R_{0}-h_{P}}\;. \end{array}$$

To model the transport of albumin across the sclera we use the Patlak equation,^63^ which describes flow and species transport across a membrane, and write

(17) $$\begin{array}{c} s_{S}=q_{S}\left(1-\sigma_{S}\right)\frac{c_{O}-c_{P}{|}_{r=R_{0}}e^{Pe_{S}}}{1-e^{Pe_{S}}},\; \end{array}$$

where the Péclet number $Pe_{S}=q_{S}\left(1-\sigma_{S}\right)/\beta_{S}$. Here, $\beta_{S}$ is the permeation coefficient per unit area of the scleral tissue multiplied by *R*_*g*_*T*, which we term the albumin conductance, $\sigma_{S}$ is the reflection coefficient of the scleral tissue, and $c_{O}$ is the orbital concentration, and $q_{S}$ is the IF Darcy velocity across the sclera, given by Equation (5).

Finally, we use the boundary conditions

(18) $${\left.\frac{\partial c_{C}}{\partial \theta }\right|}_{\theta =\theta_{0}}={\left.\frac{\partial c_{P}}{\partial \theta }\right|}_{\theta =\theta_{0}}=0,\;\;\;{\left.\frac{\partial c_{C}}{\partial \theta }\right|}_{\theta =0}={\left.\frac{\partial c_{P}}{\partial \theta }\right|}_{\theta =0}=0,$$

where (18a) follows from the assumption that the IF in the iris root and ciliary muscle is well mixed and (18b) is required for continuity of the diffusive flux of albumin.

#### Simplification of Equations

We take advantage of the fact that the lengthscales of the fluid domain in the radial and azimuthal directions are very different, with the aspect ratio being $\varepsilon_{C}=h_{C0}/R_{0}\ll 1$. We thus scale the equations using appropriate different lengthscales in the two coordinate directions. In the choroidal tissue the radial lengthscale is $h_{C0}$ and we scale the θ-velocity on $U_{C}=Q_{0}/\left(10\times 2\pi R_{0}h_{C0}\right)$, where *Q*_0_ is the aqueous humour production rate and the factor 10 is included so that the typical unconventional flow is approximately 10% of the total aqueous flow. The corresponding pressure scale is $P_{0}=\mu R_{0}U_{C}/k_{C}$ and radial velocity scale is $\varepsilon_{C}U_{C}$, and we scale the other variables accordingly. The radial component of Equation (1b) shows that the pressure is independent of the radial coordinate to leading order in $\varepsilon_{C}$.

In the SCS, we note that the pressure drop over a nominal length *R*_0_ of choroidal tissue is $\mu R_{0}U_{C}/k_{C}$. Using this as the scale for the pressure and with the corresponding θ-velocity scale $h_{P0}^{2}U_{C}/k_{C}$, the pressure $p_{P}$ is again independent of the radial coordinate to leading order. We solve for the radial dependence of the components of $u_{C}$ in the choroidal tissue in terms of $p_{C}$ and its derivatives and in the SCS we solve for $u_{P}$ in terms of $p_{P}$ and its derivatives, in each case finding explicit expressions for the radial dependence. Matching the pressures in the choroidal tissue and SCS yields $p_{C}=p_{R}=p\left(\theta \right)$, along with first-order ordinary differential equations governing the IF pressure, *p*, in and flow rate, *Q*, along the choroid–SCS.

We scale the albumin concentration on *c*_0_, the albumin concentration in the CC ($c_{0}=c_{\mathrm{CC}}$), and scale the other variables correspondingly. We find that $c_{C}$ and $c_{P}$ are independent of the radial coordinate to leading order, and, after some work, can show that $c_{C}=c_{P}=c\left(\theta \right)$, and find first-order ordinary differential equations governing *c* and *J*, where *J* is the albumin flow rate along the choroidal tissue.

The resulting governing equations and boundary conditions for the four variables *p*, *Q*, *c*, *J* represent a great simplification over the full model (1)–(18), and they can be written:

(19) $$\frac{dp}{d\theta }=\frac{\mu Q}{2\pi \sin\theta (k_{C}h_{C}+h_{P}^{3}/12)},$$

(20) $$\frac{dQ}{d\theta }=-2\pi R_{0}^{2}\sin\theta \left(h_{C}q_{C}+q_{R}-q_{S}\right),$$

(21) $$\frac{dc}{d\theta }=\frac{J-cQ}{2\pi \sin\theta (D_{C}h_{C}+D_{P}h_{P})},$$

(22) $$\frac{dJ}{d\theta }=-2\pi R_{0}^{2}\sin\theta \left(h_{C}s_{C}-s_{S}\right).$$

Equations (20) and (22) express conservation of fluid and albumin, respectively. Equation (19) states that the pressure gradient is proportional to volumetric flow rate times the resistance to the flow, and is derived from integrating the θ-velocity from Darcy’s law in the choroidal tissue Equation (1) and Stokes flow in the SCS Equation (4) over the thicknesses of these layers (multiplied by 2π*R*_0_sin θ) to obtain an expression for *Q* that can be rearranged. Equation (21) gives an expression for the concentration gradient, and is derived from integrating Fick’s law Equations (12b) and (13b) over the thickness of the choroid–SCS (also multiplied by 2π*R*_0_sin θ) to obtain an expression for *J* that can be rearranged. The boundary conditions are *Q* = *J* = 0 at θ = 0 and IOP − *p* = *R*_*icm*_*Q*, *J* = *cQ* at θ = θ_0_, and references for definitions of the symbols appearing in Equations (19)–(22) are provided in Table A1.

**Table A1.** Table Giving References for Expressions Appearing in Equations (19)–(22)

Symbol	Description	Reference Equation
$h_{P}$	Thickness of SCS	(3)
$q_{C}$	Volumetric flow rate out of vessels	(2)
$q_{R}$	Volumetric flow rate across RPE	Prescribed
$q_{S}$	Volumetric flow rate across sclera	(5)
$s_{C}$	Species flow rate out of vessels	(14)
$s_{S}$	Species flow rate across sclera	(17)

Regularity at θ = 0 means the functions $h_{C}$, $h_{P}$, *p*, *c*, *Q*, and *J* are even functions of θ, and we avoided numerical singularities at θ = 0 (arising from division by sin θ) by solving on the domain [δθ, θ_0_], where δθ = θ_0_/*n*_pts_ is the grid spacing and *n*_pts_ is the number of grid points in θ. Note that, during the solution process, the solver bvp4c chooses a mesh adaptively, based on the residual of the continuous solution.

This formulation has a major advantage over the equations before simplification in that it may readily be rapidly solved using a simple numerical scheme. When solving these equations with bvp4c, it was our experience that the choice of the initial condition could be important for convergence of the program. Where possible, we changed parameter values in small steps, using the solution from the previous step as the initial condition for the current step.

### Estimation of Parameter Values

The model relies on a number of parameters, some of which are difficult to estimate, owing to the sparse availability of experimental measurements. The model parameters are reported in Table 1, and, for those parameters requiring explanation, we report our reasoning in this Section.

#### Geometry

We model the eye internal to the sclera as a perfect sphere. To find an estimate for the outer scleral radius *R*_0_, we note that typical healthy eye diameters in adult humans are 24.2 mm (transverse) and 23.7 mm (sagittal).^73^ We average these and subtract a typical scleral thickness of $h_{S}=0.5$ mm (measured as between [0.37,0.86] mm).^72^ Our expression for the choroidal thickness profile was derived by fitting a cubic polynomial to measurements in human emmetropes;^69^ details are given in the Supplementary Material.

The thickness of the SCS is small and difficult to estimate experimentally. We choose a value of $h_{P0}$ to provide an equal flow through the choroidal tissue and SCS, so that both flow rates are comparable, and we include the dependency of the flow rate on this value in Figure A1A (we discuss this figure in greater detail elsewhere).

To convert values of hydraulic and albumin conductances of the CC from the values in individual vessels (given in the literature) to a value per unit volume of choroidal tissue (required by the model), we need to multiply by $S_{\mathrm{CC}}$, the surface area of the CC per unit volume of tissue. We examine two ways to estimate $S_{\mathrm{CC}}$. First, modeling the CC as being composed of vessels of circular cross-section, we estimate $S_{\mathrm{CC}}=4\phi /d$, where ϕ is the volume fraction of the CC and *d* are their diameters. Denoting Φ as the area fraction occupied by the septae (the voids between capillaries), we obtain the volume fraction of the CC as $\left(1-\Phi \right)d/h_{C}$, and hence $S_{\mathrm{CC}}=4\left(1-\Phi \right)/h_{C}$. Second, the model of Zouache et al.^87^ (ignoring the surface areas of the feeding arterioles and draining venules) gives a surface area to volume ratio of $S_{\mathrm{CC}}=2\left(1+\left(2d/d_{s}-1\right)\Phi \right)/h_{C}$, where *d*_*s*_ is the septal diameter, and the authors quote *d* = 20 µm, *d*_*s*_ = 3 to 24 µm and Φ = 0.25 to 0.50. These two methods give values of $S_{\mathrm{CC}}$ in the ranges 10,000 to 15,000 m^−1^ and 12,000 to 72,000 m^−1^, respectively; we take $S_{\mathrm{CC}}=12,000$ m^−1^.

#### Fluid

The Darcy permeability of human scleral tissue was estimated as 19 · 10^−13^ cm^4^ dyn^−1^ s^−1^,^95^ and, multiplying by the dynamic viscosity, this gives an estimate of $k_{S}$ as 1.33 · 10^−18^ m^2^; $k_{S}$ was also estimated as 5.85 · 10^−18^ m^2^.^25^ We use the latter value, because those measurements are more recent and study the effect of this parameter in the sensitivity analysis.

Little is known about the Darcy permeability of the choroidal tissue, $k_{C}$, except that it is larger than that of the sclera,^96^ and little is known about the resistance to flow of the tissue of the iris root and ciliary muscle, *R*_*icm*_. In addition, little is known about the SCS thickness $h_{P0}$; for example, in their experiments on rabbits, Chiang et al.^97^ assumed that the physiological SCS thickness was less than 25 µm, which is not specific enough for our model. To set these three unknown model parameters for the reference physiological case, we (1) make the model match the IF pressures at the inlet and the pole to the values measured by Emi et al., which (for physiological values of the IOP) are 0.8 ± 0.2 mm Hg and 3.7 ± 0.5 mm Hg lower than the IOP at the anterior and posterior of the SCS, respectively,^58^ and (2) set the proportions of the unconventional flow that pass through the choroidal tissue and the SCS at the inlet. This means that, if we change this proportion, we also need to adjust the parameters $k_{C}$ and *R*_*icm*_ to reproduce the pressures at the inlet and the pole. In Figure A1A, we show how changing this proportion (and correspondingly changing $k_{C}$ and *R*_*icm*_) affects the unconventional flow. Even over the range 5% to 100%, the unconventional flow only changes by appropriately 10%, suggesting that the model is not very sensitive to the chosen value of this proportion. In summary, we choose *k_C_*, *R_icm_* and *h_P0_* so that the IF pressures at the inlet and posterior are 14.2 and 11.3 mm Hg, respectively, and the proportion of the unconventional flow that passes through the choroidal tissue is 50% at the inlet.

For the hydraulic conductivity of the CC, $L_{p,\mathrm{CC}}$, we choose the value $L_{p,\mathrm{CC}}=8\cdot 10^{-11}$ m/s/Pa, which is of the same order of magnitude as that for other fenestrated capillaries (e.g., renal peritubular capillaries 4.4 · 10^−10^ m/s/Pa and intestinal mucosa 3.8 · 10^−11^ m/s/Pa),^77^ and investigate its effect with our sensitivity analysis.

Measured flow through the RPE in humans is (261 ± 130) µL/cm^2^/day ≈ (3.0 ± 1.5) · 10^−8^ m/s,^98^ in monkeys is 0.14 or 0.36 µL/mm^2^/h ≈ 3.9 · 10^−8^ or 1.0 · 10^−7^ m/s,^99^^,^^100^ and in human fetal retinal pigment epithelia in vitro is 10 µL/cm^2^/h =2.8 · 10^−8^ m/s.^71^ We use the value $q_{R}=3\cdot 10^{-8}$ m/s, which is similar to these values, and investigate the effect of this choice in the sensitivity analysis.

#### Pressures

In rabbit studies, for IOPs greater than approximately 10–15 mm Hg and up to more than 100 mm Hg, the choroidal venous pressure was shown to be slightly above the IOP.^80^ Later work confirmed these findings, showing the choroidal venous pressure is around 3 cmH_2_O above the IOP (IOP + 2 mm Hg) and that the CC pressures are 7 to 13 cmH_2_O above the IOP (IOP + 5 to IOP + 10 mm Hg).^47^ In our model, we set the pressure in the CC to be $p_{\mathrm{CC}}=\text{IOP}+5$ mm Hg, which is the lower boundary of the measured values. We study the effect of this parameter in the sensitivity analysis.

We take the orbital pressure to be $p_{O}=3$ mm Hg, which is within the typical range in the literature of 2 to 6 mm Hg,^74^ and investigate the effect of the uncertainty in the value in the sensitivity analysis.

As discussed in the General Description of the Model, we assume that, under normal physiological conditions, the SCS has uniform prescribed thickness $h_{P0}$. The SCS thickness responds elastically if the IOP changes. We choose the parameter characterizing the elasticity, see Equation (3), as $\lambda_{P}=100$ Pa. As mentioned in the Parameter values, this value was chosen so that there is significant expansion or collapse of the SCS over the range of the IOP considered in this article (5–25 mm Hg). The role of this parameter is investigated in Figure A1B, where the dependency of flow rate on the IOP is shown for three values of $\lambda_{P}$. The blue curve ($\lambda_{P}=100$ Pa) is the same as that in Figure 5. Figure 7B shows that for $\lambda_{P}=1000$ Pa, the SCS is stiff and the response to the IOP increase is almost linear. For $\lambda_{P}=10$ Pa, the SCS is more flexible and the flow exhibits more non-linearity for small IOP, and shows a slower increase with the IOP at high values of the IOP than in the stiff case.

We assume that the retinal pressure $p_{R}=\mathrm{IOP}$; however, there is some variation in pressure between the anterior and posterior of the eye owing to the slow flow of fluid through the vitreous humor, requiring a pressure drop to drive it. In addition, there is a flow of IF outward through the retina, causing an additional pressure drop over the thickness of the retina. We compare the flow with $p_{R}=\mathrm{IOP}$ with that for $p_{R}=\mathrm{IOP}-0.5$ mm Hg in Figure A1B, which suggests the choice of $p_{R}$ has little effect.

#### Albumin

The albumin concentration in blood vessels is approximately 44 g/L,^76^ giving $c_{\mathrm{CC}}=0.66$ mM (molecular weight of albumin is 66.5 kDa). We could not find a reference for albumin concentration in the orbit, but the concentration in human choroidal tissue was given as 35% of the plasma value (although measurements in only three eyes were reported, and these varied very widely) and that in the scleral tissue as 19% (in this case, four measurements were given, which varied widely),^19^ whereas in the monkey the choroidal tissue concentration was given as 10% of that in plasma.^18^ In this article, we fix the orbital concentration at 19% of the blood plasma value and vary it in the sensitivity analysis (Fig. 4).

The albumin conductance of a fenestrated capillary is taken as $P_{d,\mathrm{CC}}=8\cdot 10^{-10}$ m/s, which is larger than the value 4 · 10^−10^ m/s reported by Levick^21^ for fenestrated capillaries. We choose a higher value because there are a vast number of caveolae present in the CC,^20^ which will enhance albumin transport by transcytosis. With this value of $P_{d,\mathrm{CC}}$, we calculate an albumin flux from the CC of 10.6 · 10^−13^ mol/s, which is in good agreement with 9.35 · 10^−13^ mol/s found by Bill in a rabbit eye (0.0847 µL/min of blood plasma albumin).^78^ We check the dependency of the model on $P_{d,\mathrm{CC}}$ in the sensitivity analysis.

The osmotic reflection coefficient for transport across the capillary walls is taken as $\sigma_{\mathrm{CC}}=0.95$, which is in line with the results of Michel et al.,^64^ who report values of the reflection coefficient for albumin at least 0.9 for vessels with high hydraulic permeability (over about 6 · 10^−11^ m/s/Pa).

Anderson et al.^75^ reported the partition coefficient for the sclera as 0.62, which was found by leaving the scleral tissue in a solution containing albumin; the partition coefficient was the concentration of albumin in the tissue divided by that in the solution. This implies a reflection coefficient $\sigma_{S}=0.38$. The albumin conductance across human sclera was measured as $\beta_{S}=8.3\cdot 10^{-9}$ m/s.^75^

#### Sensitivity Analysis

The details of the eFAST method are not reported here for brevity, as they are clearly described by Saltelli et al.^65^ and Marino et al.^66^ In this method, each considered parameter is sampled from a uniform distribution within its corresponding range; the ranges are reported in Table A2. The sampling of each parameter follows a periodic curve with a certain frequency. For each parameter, five independent curves are considered, each with a random phase shift. Each curve samples 1000 points. We computed the sensitivity of the unconventional flow rate and the average albumin concentration to variations in selected parameters.

**Table A2.** Ranges Considered for Each Parameter in the Sensitivity Analysis

Parameter	Range	Units
$P_{d,\mathrm{CC}}$	[4, 12] · 10^−10^	m^−1^
$k_{C}$	[5.85, 17.6] · 10^−15^	m^2^
$q_{R}$	[1.5, 4.5] · 10^−8^	m/s
$p_{O}$	[3, 6]	mm Hg
$p_{\mathrm{CC}}$	[18, 22]	mm Hg
$k_{S}$	[2.93, 8.78] · 10^−18^	m^2^
$L_{p,\mathrm{CC}}$	[4, 12] · 10^−11^	m/s/Pa
*R*_*icm*_	[0.63, 1.9]	mm Hg/(µL/min)
$c_{O}$	[0.076, 0.176]	mM
$h_{P0}$	[1.2,3.6]	µm

The dummy parameter *d* is varied between 0 and 1.

We will outline here a rigorous definition of the SIs. Consider the input parameters *X* = (*X*_1_, *X*_2_, ..., *X*_*n*_) and the output *Y*, so that *Y* = *f*(*X*), where *f* is the model. The first-order SI for parameter *i* is defined as

(23) $$\begin{array}{c} S_{i}=\frac{V_{i}}{V}. \end{array}$$

In this expression, *V* = Var(*Y*) is the total variance of *Y*, and *V*_*i*_ is the first-order contribution of parameter *i* in the variance decomposition. This is defined as *V*_*i*_ = Var(*E*(*Y*|*X*_*i*_)),^65^ where, according to Bayesian notation, *E*(*Y*|*X*_*i*_) is the expected value of *Y* conditional on a fixed value of *X*_*i*_ (note that this is a random variable, not a simple number). Because *V* = Var(*E*(*Y*|*X*_*i*_)) + *E*(Var(*Y*|*X*_*i*_)), the quantity *V*_*i*_ describes the expected reduction in the variance of the model output if parameter *i* was fixed.

The total SI for parameter *i* is defined as

(24) $$S_{Ti}=\frac{E(\mathrm{Var}\left(Y|X_{∼i}\right))}{V},$$

where *X*_∼*i*_ denotes a set of all parameters except *i*. The numerator, therefore, represents the expected variance that would remain if all parameters but parameter *i* are kept fixed.

The dummy parameter is a variable that does not appear in the model. It is included as a form of negative control.

## References

[1] Nesterov AP . 13. The future for surgery in the glaucomas by increasing uveoscleral outflow. In: Cairns JE, ed. *Glaucoma*. New York: Grune & Stratton; 1986: 257–273.

[2] Bill A . The drainage of blood from the uvea and the elimination of aqueous humour in rabbits. *Exp Eye Res.* 1962; 1: 200–205. 13869187 10.1016/s0014-4835(62)80002-5

[3] Bill A . The aqueous humor drainage mechanism in the cynomolgus monkey (Macaca irus) with evidence for unconventional routes. *Invest Ophthalmol Vis Sci.* 1965; 4: 911–919. 4157891

[4] Bill A, Hellsing K. Production and drainage of aqueous humor in the cynomolgus money (Macaca irus). *Invest Ophthalmol Vis Sci.* 1965; 4: 920–926. 4157892

[5] Bill A . Conventional and uveo-scleral drainage of aqueous humour in the cynomolgus monkey (Macaca irus) at normal and high intraocular pressures. *Exp Eye Res.* 1966; 5(1): 45–54. 4160221 10.1016/s0014-4835(66)80019-2

[6] Bill A . The routes for bulk drainage of aqueous humour in rabbits with and without cyclodialysis. *Doc Ophthalmol.* 1966; 20: 157–169. 5982257 10.1007/BF00165414

[7] Bill A . The routes for bulk drainage of aqueous humour in the vervet monkey (Cercopithecus ethiops). *Exp Eye Res.* 1966; 5: 55–57. 4160407 10.1016/s0014-4835(66)80020-9

[8] Pederson JE, Toris CB. Uveoscleral outflow: diffusion or flow? *Invest Ophthalmol Vis Sci.* 1987; 28(6): 1022–1024. 2438249

[9] Alm A, Nilsson SFE. Uveoscleral outflow – A review. *Exp Eye Res.* 2009; 88(4): 760–768. 19150349 10.1016/j.exer.2008.12.012

[10] Yücel YH, Johnston MG, Ly T, et al. Identification of lymphatics in the ciliary body of the human eye: A novel “uveolymphatic” outflow pathway. *Exp Eye Res.* 2009; 89: 810–819. 19729007 10.1016/j.exer.2009.08.010

[11] Schroedl F, Kaser-Eichberger A, Schlereth SL, et al. Consensus statement on the immunohistochemical detection of ocular lymphatic vessels. *Invest Ophthlamol Vis Sci.* 2014; 55(10): 6440–6442. 10.1167/iovs.14-15638 PMC4580075 25315233

[12] Chowdhury UR, Madden BJ, Charlesworth MC, Fautsch MP. Proteome analysis of human aqueous humor. *Invest Ophthalmol Vis Sci.* 2010; 51: 4921–4931. 20463327 10.1167/iovs.10-5531 PMC3066620

[13] Tripathi RC, Millard CB, Tripathi BJ. Protein composition of human aqueous humor: SDS-PAGE analysis of surgical and post-mortem samples. *Exp Eye Res.* 1989; 48: 117–130. 2920779 10.1016/0014-4835(89)90025-0

[14] Freddo TF, Dartels SP, Darsotti MF, Kamm RD. The source of proteins in the aqueous humor of the normal rabbit. *Invest Ophthalmol Vis Sci.* 1990; 31(1): 125–137. 2298533

[15] Raviola G . The structural basis of the blood–ocular barriers. *Exp Eye Res.* 1977; 25: 27–63. 412691 10.1016/s0014-4835(77)80009-2

[16] Bill A . Capillary permeability to and extravascular dynamics of myoglobin, albumin and gammaglobulin in the uvea. *Acta Physiol Scand.* 1968; 73(1): 204–219. 4175033 10.1111/j.1748-1716.1968.tb04097.x

[17] Freddo TF . A contemporary concept of the blood-aqueous barrier. *Prog Retin Eye Res.* 2013; 32: 181–195. 23128417 10.1016/j.preteyeres.2012.10.004 PMC3544162

[18] Toris CB, Pederson JE, Tsuboi S, Gregerson DS, Rice TJ. Extravascular albumin concentration of the uvea. *Invest Ophthalmol Vis Sci.* 1990; 31(1): 43–53. 2298542

[19] Allansmith MR, Whitney CR, McClellan BH, Newman LP. Immunoglobulins in the human eye: location, type, and amount. *Arch Ophthalmol.* 1973; 89(1): 36–45. 4630887 10.1001/archopht.1973.01000040038010

[20] Nakanishi M, G. R, Bhutto IA, Edwards M, McLeod DS, Lutty GA. Albumen transport to Bruch's membrane and rpe by choriocapillaris caveolae. *Invest Ophthalmol Vis Sci.* 2016; 57(4): 2213–2224. 27116549 10.1167/iovs.15-17934 PMC4849865

[21] Herring N, Paterson DJ, *Levick's Introduction to Cardiovascular Physiology*. 6th ed. Boca Raton, FL: CRC Press; 2018.

[22] Krohn J, Bertelsen T. Corrosion casts of the suprachoroidal space and uveoscleral drainage routes in the human eye. *Acta Physiol Scand.* 1997; 75(1): 32–35. 10.1111/j.1600-0420.1997.tb00245.x 9088397

[23] Krohn J, Bertelsen T. Light microscopy of uveoscleral drainage routes after gelatine injections into the suprachoroidal space. *Acta Physiol Scand.* 1998; 76(5): 521–527. 10.1034/j.1600-0420.1998.760502.x 9826032

[24] Deng W, Liu C, Parra C, et al. Quantitative imaging of the clearance systems in the eye and the brain. *Quant Imaging Med Surg.* 2020; 10(1): 1. 31956524 10.21037/qims.2019.11.18 PMC6960420

[25] Jackson TL, Hussain A, Hodgetts A, et al. Human scleral hydraulic conductivity: age-related changes, topographical variation, and potential scleral outflow facility. *Invest Ophthalmol Vis Sci.* 2006; 47(11): 4942–4946. 17065511 10.1167/iovs.06-0362

[26] Levick JR . Capillary filtration-absorption balance reconsidered in light of dynamic extravascular factors. *Exp Physiol Transl Integr.* 1991; 76(6): 825–857. 10.1113/expphysiol.1991.sp003549 1768414

[27] Pederson JE, Gaasterland DE, MacLellan HM. Uveoscleral aqueous outflow in the rhesus monkey: importance of uveal reabsorption. *Invest Ophthalmol Vis Sci.* 1977; 16(11): 1008–1017. 410750

[28] Sherman SH, Green K, Laties AM. The fate of anterior chamber flurescein in the monkey eye. 1. The anterior chamber outflow pathways. *Exp Eye Res.* 1978; 27(2): 159–173. 98337 10.1016/0014-4835(78)90086-6

[29] Poyer JF, Millar C, Kaufman PL. Prostaglandin F2α effects on isolated rhesus monkey ciliary muscle. *Invest Ophthalmol Vis Sci.* 1995; 36(12): 2461–2465. 7591635

[30] Lütjen-Drecoll E, Tamm E. Morphological study of the anterior segment of cynomolgus monkey eyes following treatment with prostaglandin F2α. *Exp Eye Res.* 1988; 47: 761–769. 3197775 10.1016/0014-4835(88)90043-7

[31] Weinreb RN, Kashiwagi K, Kashiwagi F, Tsukahara S, Lindsey JD. Prostaglandins increase matrix metalloproteinase release from human ciliary smooth muscle cells. *Invest Ophthalmol Vis Sci.* 1997; 38(13): 2772–2780. 9418730

[32] Kim J-W, Lindsey JD, Wang N, N.Weinreb R. Increased human scleral permeability with prostaglandin exposure. *Invest Ophthlamol Vis Sci.* 2001; 42(7): 1514–1521. 11381055

[33] Gaton DD, Sagara T, Lindsey JD, Weinreb RN. Matrix metalloproteinase-1 localization in the normal human uveoscleral outflow pathway. *Invest Ophthlamol Vis Sci.* 1999; 40(2): 363–369. 9950594

[34] Stjernschantz J, Selén G, Astin M, Resul B. Microvascular effects of selective prostaglandin analogues in the eye with special reference to latanoprost and glaucoma treatment. *Prog Retin Eye Res.* 2000; 19: 459–496. 10785618 10.1016/s1350-9462(00)00003-3

[35] Payne DK, Fuseler JW, Owens MW. Modulation of endothelial cell permeability by lung carcinoma cells: A potential mechanism of malignant pleural effusion formation. *Inflammation.* 1994; 18(4): 407–417. 7982730 10.1007/BF01534438

[36] Svensjö E . Bradykinin and prostaglandin E1, E2 and F2α-induced macromolecular leakage in the hamster cheek pouch. *Prostaglandins Med.* 1978; 1(5): 397–410. 724817 10.1016/0161-4630(78)90126-x

[37] Alexander JS, Elrod JW. Extracellular matrix, junctional integrity and matrix metalloproteinase interactions in endothelial permeability regulation. *J Anat.* 2002; 200(6): 561–574. 12162724 10.1046/j.1469-7580.2002.00057.x PMC1570742

[38] Duru Z, Özsaygılı C, Ulusoy DM, Demirtaş AA, Çiçek A, Duru N. Does using topical latanoprost affect subfoveal choroidal thickness? *Cutan Ocul Toxicol.* 2019; 38: 370–374. 31213097 10.1080/15569527.2019.1632884

[39] Crawford K, Kaufman PL. Pilocarpine antagonizes prostaglandin F2α-induced ocular hypotension in monkeys: Evidence for enhancement of uveoscleral outflow by prostaglandin F2α. *Arch Ophthalmol*. 1987; 105: 1112–1116. 3477218 10.1001/archopht.1987.01060080114039

[40] Bill A . Effects of atropine and pilocarpine on aqueous humour dynamics in cynomoigus monkeys (macaca irus). *Exp Eye Res.* 1967; 6(2): 120–125. 4960736 10.1016/s0014-4835(67)80062-9

[41] Taylor NR, Zele AJ, Vingrys AJ, Stanley RG. Variation in intraocular pressure following application of tropicamide in three different dog breeds. *Vet Ophthalmol.* 2007; 10(s1): 8–11. 17973829 10.1111/j.1463-5224.2007.00485.x

[42] Bill A . Early effects of epinephrine on aqueous humor dynamics in vervet monkeys (Cercopithecus ethiops). *Exp Eye Res.* 1969; 8: 35–43. 4975259 10.1016/s0014-4835(69)80078-3

[43] Bill A, Walinder PE. The effects of pilocarpine on the dynamics of aqueous humor in a primate (Macaca irus). *Invest Ophthalmol Vis Sci.* 1966; 5: 170–175.

[44] Mori M, Araie M, Sakurai M, Oshika T. Effects of pilocarpine and tropicamide on blood-aqueous barrier permeability in man. *Invest Ophthalmol Vis Sci.* 1992; 33: 416–423. 1740374

[45] Brubaker RF . Goldmann's equation and clinical measures of aqueous dynamics. *Exp Eye Res.* 2004; 78: 633–637. 15106943 10.1016/j.exer.2003.07.002

[46] Johnson M, McLaren JW, Overby DR. Unconventional aqueous humor outflow: A review. *Exp Eye Res.* 2017; 158: 94–111. 26850315 10.1016/j.exer.2016.01.017 PMC4970980

[47] Mäepea O . Pressures in the anterior ciliary arteries, choroidal veins and choriocapillaris. *Exp Eye Res.* 1992; 54(5): 731–736. 1623958 10.1016/0014-4835(92)90028-q

[48] Sacco R, Guidoboni G, Jerome JW, et al. A theoretical approach for the electrochemical characterization of ciliary epithelium. *Life.* 2020; 10(2): 8. 31979304 10.3390/life10020008 PMC7175328

[49] Dvoriashyna M, Foss AJE, Gaffney EA, Repetto R. A mathematical model of aqueous humor production and composition. *Invest Opthalmol Vis Sci.* 2022; 63: 1. 10.1167/iovs.63.9.1 PMC9358295 35917134

[50] Heys JJ, Barocas VH, Taravella MJ. Modeling passive mechanical interaction between aqueous humor and iris. *J Biomech Eng.* 2001; 123(6): 540–547. 11783724 10.1115/1.1411972

[51] Fitt AD, Gonzalez G. Fluid mechanics of the human eye: aqueous humour flow in the anterior chamber. *Bull Math Biol.* 2006; 68(1): 53–71. 16794921 10.1007/s11538-005-9015-2

[52] Dvoriashyna M, Repetto R, Romano MR, Tweedy JH. Aqueous humour flow in the posterior chamber of the eye and its modifications due to pupillary block and iridotomy. *Math Med Biol.* 2018; 35(4): 447–467. 29095997 10.1093/imammb/dqx012

[53] Dvoriashyna M, Repetto R, Tweedy JH. Oscillatory and steady streaming flow in the anterior chamber of the moving eye. *J Fluid Mech.* 2019; 863: 904–926.

[54] Johnson MC, Kamm RD. The role of Schlemm's canal in aqueous outflow from the human eye. *Invest Ophthalmol Vis Sci.* 1983; 24: 320–325. 6832907

[55] Siggers JH, Ethier CR. Fluid mechanics of the eye. *Annu Rev Fluid Mech.* 2012; 44: 347–372.

[56] Dvoriashyna M, Foss AJE, Gaffney EA, Repetto R. Fluid and solute transport across the retinal pigment epithelium: a theoretical model. *J Roy Soc Interface.* 2020; 17: 20190735. 32019471 10.1098/rsif.2019.0735 PMC7061709

[57] Inomata H, Bill A. Exit sites of uveoscleral flow of aqueous humor in cynomolgus monkey eyes. *Exp Eye Res.* 1977; 25: 113–118. 410651 10.1016/0014-4835(77)90123-3

[58] Emi K, Pederson JE, Toris CB. Hydrostatic pressure of the suprachoroidal space. *Invest Ophthalmol Vis Sci.* 1989; 30(2): 233–238. 2914753

[59] Chiang B, Jung JH, Prausnitz MR. The suprachoroidal space as a route of administration to the posterior segment of the eye. *Adv Drug Deliver Rev.* 2018; 126: 58–66. 10.1016/j.addr.2018.03.001 PMC5995649 29545195

[60] Croft MA, Lütjen-Drecoll E, Kaufman PL. Age-related posterior ciliary muscle restriction – A link between trabecular meshwork and optic nerve head pathophysiology. *Exp Eye Res.* 2017; 158: 187–189. 27453343 10.1016/j.exer.2016.07.007 PMC5253323

[61] Li H-L, Ren R, Gong H. Segmental unconventional outflow in mouse eyes. *Invest Ophthalmol Vis Sci.* 2023; 64: 26. 10.1167/iovs.64.15.26 PMC10741088 38117243

[62] Platzl C, Kaser-Eichberger A, Benavente-Perez A, Schroedl F. The choroid-sclera interface: An ultrastructural study. *Heliyon.* 2022; 8(5): e09408. 35586330 10.1016/j.heliyon.2022.e09408 PMC9108890

[63] Truskey GA, Yuan F, Katz DF, *Transport Phenomena in Biological Systems*. London, UK: Pearson Education; 2004.

[64] Michel CC . Capillary permeability and how it may change. *J Physiol.* 1988; 404(1): 1–29. 3075669 10.1113/jphysiol.1988.sp017275 PMC1190811

[65] Saltelli A, Tarantola S, Chan KP-S. A quantitative model-independent method for global sensitivity analysis of model output. *Technometrics.* 1999; 41(1): 39–56.

[66] Marino S, Hogue IB, Ray CJ, Kirschner DE. A methodology for performing global uncertainty and sensitivity analysis in systems biology. *J Theor Biol.* 2008; 254(1): 178–196. 18572196 10.1016/j.jtbi.2008.04.011 PMC2570191

[67] Vogel S , *Life's devices: the physical world of animals and plants*. Princeton, NJ: Princeton University Press; 1988.

[68] Meechai N, Jamieson AM, Blackwell J. Translational diffusion coefficients of bovine serum albumin in aqueous solution at high ionic strength. *J Colloid Interface Sci.* 1999; 218(1): 167–175. 10489290 10.1006/jcis.1999.6401

[69] Hoseini-Yazdi M, Vincent SJ, Collins MJ, Read SA, Alonso-Caneiro D. Widefield choroidal thickness in myopes and emmetropes. *Sci Rep.* 2019; 9: 3474. 30837507 10.1038/s41598-019-39653-w PMC6401121

[70] Hughes BA, Miller SS, Machen TE. Effects of cyclic AMP on fluid absorption and ion transport across frog retinal pigment epithelium. Measurements in the open-circuit state. *J Gen Physiol.* 1984; 83(6): 875–899. 6330281 10.1085/jgp.83.6.875 PMC2215661

[71] Shi G, Maminishkis A, Banzon T, et al. Control of chemokine gradients by the retinal pigment epithelium. *Invest Ophthalmol Vis Sci.* 2008; 49(10): 4620–4630. 18450597 10.1167/iovs.08-1816 PMC2574653

[72] Vurgese S, Panda-Jonas S, Jonas JB. Scleral thickness in human eyes. *PLoS One.* 2012; 7(1): e29692. 22238635 10.1371/journal.pone.0029692 PMC3253100

[73] Bekerman I, Gottlieb P, Vaiman M. Variations in eyeball diameters of the healthy adults. *J Ophthalmol.* 2014; 2014: 503645. 25431659 10.1155/2014/503645 PMC4238270

[74] Enz TJ, Tschopp M. Assessment of orbital compartment pressure: A comprehensive review. *Diagnostics.* 2022; 12(6): 1481. 35741290 10.3390/diagnostics12061481 PMC9221953

[75] Anderson OA, Jackson TL, Singh JK, Hussain AA, Marshall J. Human transscleral albumin permeability and the effect of topographical location and donor age. *Invest Ophthalmol Vis Sci.* 2008; 49(9): 4041–4045. 18450593 10.1167/iovs.07-1660

[76] Narang R, Ridout D, Nonis C, Kooner JS. Serum calcium, phosphorus and albumin levels in relation to the angiographic severity of coronary artery disease. *Int J Cardiol.* 1997; 60(1): 73–79. 9209942 10.1016/s0167-5273(97)02971-9

[77] Levick JR, Smaje LH. An analysis of the permeability of a fenestra. *Microvasc Res.* 1987; 33(2): 233–256. 3587078 10.1016/0026-2862(87)90020-3

[78] Bill A . The albumin exchange in the rabbit eye. *Acta Physiol Scand.* 1964; 60(1–2): 18–29. 14131826 10.1111/j.1748-1716.1964.tb02865.x

[79] Nilsson SFE, Bill A. Part i – normal: Anterior segment. Physiology and neurophysology of aqueous humor inflow and outflow. In Kaufman PL, Mittag TW. eds. *Glaucoma,* vol. 7 of *Textbook of Ophthalmology*. Mosby-Year Book Europe Ltd., 1994.

[80] Bill A . The uveal venous pressure. *Arch Ophthalmol.* 1963; 69(6): 780–782. 13968253 10.1001/archopht.1963.00960040786021

[81] Kleinstein RN, Fatt I. Pressure dependency of transcleral flow. *Exp Eye Res.* 1977; 24: 335–340. 858317 10.1016/0014-4835(77)90146-4

[82] Tam ALC, Gupta N, Zhang Z, Yücel YH. Quantum dots trace lymphatic drainage from the mouse eye. *Nanotechnology.* 2011; 22: 425101. 21934199 10.1088/0957-4484/22/42/425101

[83] Kim YK, Na KI, Jeoung JW, Park KH. Intraocular pressure-lowering effect of latanoprost is hampered by defective cervical lymphatic drainage. *PLoS One.* 2017; 12: e0169683. 28081184 10.1371/journal.pone.0169683 PMC5231387

[84] Kaser-Eichberger A, Schrödl F, Trost A, et al. Topography of lymphatic markers in human iris and ciliary body. *Invest Ophthalmol Vis Sci.* 2015; 56: 4943–4953. 26225635 10.1167/iovs.15-16573

[85] Rowe-Rendleman CL, Durazo SA, Kompella UB, et al. Drug and gene delivery to the back of the eye: from bench to bedside. *Invest Ophthalmol Vis Sci.* 2014; 55(4): 2714–2730. 24777644 10.1167/iovs.13-13707 PMC4004426

[86] Alm A . Uveoscleral outflow. *Eye.* 2000; 14: 488491. 10.1038/eye.2000.135 11026978

[87] Zouache MA, Eames I, Luthert PJ. Blood flow in the choriocapillaris. *J Fluid Mech.* 2015; 774: 37–66.

[88] Wajer SD, Taomoto M, McLeod DS, et al. Velocity measurements of normal and sickle red blood cells in the rat retinal and choroidal vasculatures. *Microvas Res.* 2000; 60(3): 281–293. 10.1006/mvre.2000.2270 11078644

[89] Cancelli C, Pedley TJ. A separated-flow model for collapsible-tube oscillations. *J Fluid Mech.* 1985; 157: 375–404.

[90] Brook BS, Falle SAEG, Pedley TJ. Numerical solutions for unsteady gravitydriven flows in collapsible tubes: evolution and roll-wave instability of a steady state. *J Fluid Mech.* 1999; 396: 223–256.

[91] Toro EF, Siviglia A. Simplified blood flow model with discontinuous vessel properties: analysis and exact solutions. In:Ambrosi G, Quarteroni A, Rozza G. eds. *Modeling of Physiological Flows: Modeling, Simulation and Applications*. vol. 5. Milano: Springer; 2012: 19–39.

[92] Toro EF . Brain venous haemodynamics, neurological diseases and mathematical modelling: A review. *Appl Math Comput.* 2016; 272: 542–579.

[93] Hamann S . Molecular mechanisms of water transport in the eye. *Int Rev Cytol.* 2002; 215: 395–431. 11952236 10.1016/s0074-7696(02)15016-9

[94] Kedem O, Katchalsky A. Thermodynamic analysis of the permeability of biological membranes to nonelectrolytes. *Biochim Biophys Acta.* 1958; 27: 229–246. 13522722 10.1016/0006-3002(58)90330-5

[95] Fatt I, Hedbys BO. Flow of water in the sclera. *Exp Eye Res.* 1970; 10(2): 243–249. 5484767 10.1016/s0014-4835(70)80035-5

[96] Ranta V-P, Urtti A. Transscleral drug delivery to the posterior eye: prospects of pharmacokinetic modeling. *Adv Drug Deliv Rev.* 2006; 58(11): 1164–1181. 17069929 10.1016/j.addr.2006.07.025

[97] Chiang B, Venugopal N, Grossniklaus HE, Jung JH, Edelhauser HF, Prausnitz MR. Thickness and closure kinetics of the suprachoroidal space following microneedle injection of liquid formulations. *Invest Ophthalmol Vis Sci.* 2017; 58: 555–564. 28125842 10.1167/iovs.16-20377 PMC5283084

[98] Chihara E, Nao-i N. Resorption of subretinal fluid by transepithelial flow of the retinal pigment epithelium. *Graef Arch Clin Exp.* 1985; 223: 202–204. 10.1007/BF02174060 3902580

[99] Pederson JE, Cantrill HL. Experimental retinal detachment: V. Fluid movement through the retinal hole. *Arch Ophthalmol.* 1984; 102(1): 136–139. 6703957 10.1001/archopht.1984.01040030114048

[100] Cantrill HL, Pederson JE. Experimental retinal detachment: VI. The permeability of the blood-retinal barrier. *Arch Ophthalmol.* 1984; 102(5): 747–751. 6721768 10.1001/archopht.1984.01040030595029

