# Supplementary material for: A Model of the Mechanisms Underpinning Unconventional Aqueous Humor Outflow
Source: Invest Ophthalmol Vis Sci. 2025 Apr 28;66(4):75. doi: 10.1167/iovs.66.4.75 (PMC12060054; doi:10.1167/iovs.66.4.75)
Supplement: Supplement 1 [file iovs-66-4-75_s001.pdf]

A model of the mechanisms underpinning unconventional aqueous humor outflow: Supplementary Material

Jennifer H. Tweedy, Mariia Dvoriashyna, Jessica R. Crawshaw, Darryl R. Overby, Rodolfo Repetto, Paul A. Roberts, Tamsin A. Spelman, Peter S. Stewart, Alexander J. E. Foss

March 11, 2024,
with revisions on October 1, 2024, January 24 and March 24, 2025

Contents

1 Introduction **2**

2 Problem formulation **2**
2.1 IF flow 2
2.2 Albumin transport 6

3 Simplification of IF flow equations for small thickness of the domain **7**
3.1 Flow in the choroidal tissue 7
3.2 Flow in Case (i) 9
3.3 Analysis of flow in Case (ii) 9
3.3.1 Flow in SCS in Case (ii) 9
3.3.2 Matching conditions for flow in Case (ii) 10
3.4 Analysis of flow in Case (iii) 10
3.4.1 Flow in SCS in Case (iii) 10
3.4.2 Matching conditions for flow in Case (iii) 11

4 Simplification of albumin transport equations for small thickness of the domain **12**
4.1 Analysis of transport in the choroidal tissue 12
4.2 Analysis of transport in Case (i) 12
4.3 Analysis of transport in Case (ii) 13
4.3.1 Transport in the SCS in Case (ii) 13
4.3.2 Matching conditions for transport in Case (ii) 14
4.4 Analysis of transport in Case (iii) 14
4.4.1 Transport in the SCS in Case (iii) 14
4.4.2 Matching conditions for transport in Case (iii) 15

5 Summary **16**

6 Use of a Beavers–Joseph boundary condition **19**

7 Fitting the choroidal thickness profile **19**

1 Introduction

This is the Supplementary Material for our paper of the same name appearing in the journal *Investigative Ophthalmology and Visual Science*.

In Sections 2–5 of this document, we derive the model equations used in the main text; this represents an expansion of the presentation of the model equations appearing in Appendix A of the paper to a full mathematical derivation from first principles. In Section 2, we present the mathematical model equations in full. We then simplify the equations for the flow (Section 3) and albumin transport (Section 4) accounting for the separation of lengthscales between the thickness of the choroid (and that of the suprachoroidal space, SCS, if present) and the arc length of the model from anterior to posterior. We summarize the resulting governing equations in Section 5.

In Section 6, we derive the analogous equations using a more general condition at the interface of the choroidal tissue and the SCS. Finally, in Section 7, we derive a suitable expression for the thickness of the choroid, as a function of distance from the anterior.

2 Problem formulation

We develop a mathematical model of interstitial fluid (IF) flow and albumin transport in the tissue of the choroid and the SCS in steady state (see Fig. 1 in the main text and Fig. S1). We model the sclera as a rigid spherical shell of uniform thickness, $h_{\mathcal{S}}$, that is permeable to IF and albumin, while we model the choroidal tissue as a perfused layer of tissue of variable thickness, $h_{\mathcal{C}}$. Between the choroid and sclera we allow for a suprachoroidal space (SCS) whose thickness, $h_{\mathcal{P}}$, can change in response to changes in the IF pressure. The inner surface of the choroid is bounded by the retinal pigment epithelium (RPE), across which IF is pumped at a constant rate. We assume that capillaries in the choriocapillaris (CC) are homogeneously distributed throughout the choroidal tissue, and fluid and albumin can exchange locally between the CC and interstitium of the choroidal tissue.

In this model we account for the outer retina (denoted with subscript $\mathcal{R}$), choroid (subscript $\mathcal{C}$), SCS (subscript $\mathcal{P}$), sclera (subscript $\mathcal{S}$) and orbit (subscript $\mathcal{O}$). Throughout the choroid, we assume that the capillaries of the CC are homogeneously distributed (subscript $\mathcal{CC}$).

The inner surface of the sclera is assumed perfectly spherical with radius R_0, and the centre of this sphere is used as the origin of a spherical coordinate system (r, θ, ϕ); r denotes the radial distance from the centre, θ is the polar angle, with $\theta = 0$ pointing to the posterior pole, and ϕ is the azimuthal angle. We restrict attention to axisymmetric flow (ignoring variations with respect to the azimuthal angle, ϕ, as well as flows in this direction). The polar angle spans from the posterior pole ($\theta = 0$) to the anterior limit of the choroid at the iris root ($\theta = \theta_0$). In this coordinate system, the choroid occupies $R_0 - h_{\mathcal{P}} - h_{\mathcal{C}} < r < R_0 - h_{\mathcal{P}}$, the SCS occupies $R_0 - h_{\mathcal{P}} < r < R_0$ and the sclera occupies $R_0 < r < R_0 + h_{\mathcal{S}}$, where $h_{\mathcal{C}}(\theta)$, $h_{\mathcal{P}}(\theta)$, $h_{\mathcal{S}}$ denote the thickness of the choroid, SCS and sclera respectively.

We now outline the governing equations for our model, starting with those governing IF flow (Section 2.1), followed by those for albumin concentration (Section 2.2).

2.1 IF flow

Within the choroidal tissue and the SCS, we model IF flow as Newtonian and incompressible. We also incorporate the transport of albumin (as will be discussed in Section 2.2).

Overall, two mechanisms drive this flow of IF. Firstly, a pressure difference between the inlet of the model (close to the intraocular pressure, IOP) and the orbit (denoted $p_{\mathcal{O}}$) drives flow posteriorly in the choroid–SCS, as well as outflow from the choroidal tissue across the

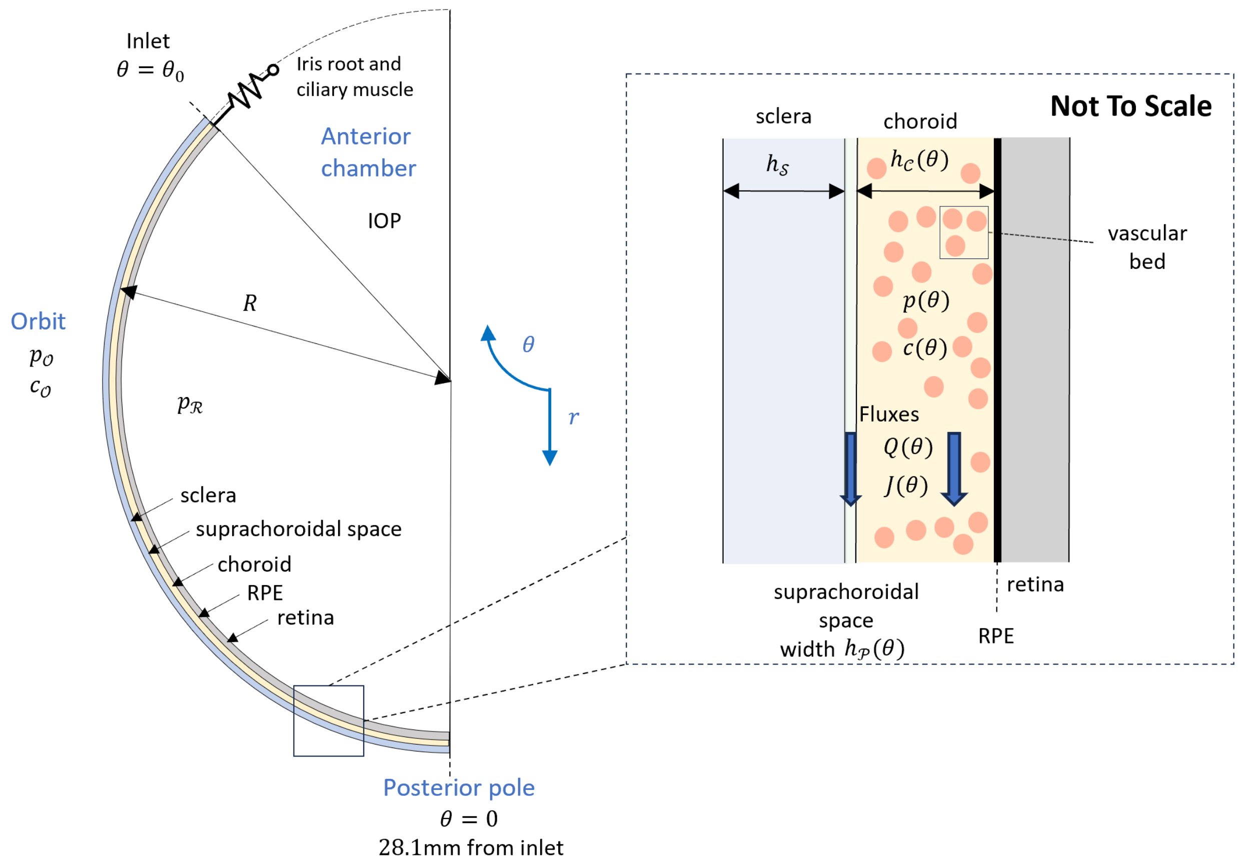

Figure S1: Schematic diagram of the eye highlighting the set up and parameters used in the mathematical model. The anterior of the eye is at the top of the diagram and the model has rotational symmetry about the vertical axis $\theta = 0$. The inset zooms into the region we focus on in the model, which is the outer part of the posterior eye.

sclera. Secondly, mass transport between the choriocapillaris and the interstitium results in local sources and sinks of IF, which drive IF flow along the choroidal tissue. To model fluid and solute exchange with the vessels of the choroid, we focus particularly on the fenestrated CC. We assume that the CC is uniformly distributed throughout the choroidal tissue, and that it has uniform properties within the layer. We now discuss the mathematical model within the choroidal tissue and the SCS in turn.

To model IF flow within the choroid, we assume Darcy flow modeling flow in a porous medium with Darcy velocity vector $\mathbf{u}_\mathcal{C} = (u_{\mathcal{C}r}, u_{\mathcal{C}\theta}, u_{\mathcal{C}\phi})$ (volume flow rate per unit area) in spherical coordinates

$$\boldsymbol{\nabla} \cdot \mathbf{u}_\mathcal{C} = q_\mathcal{C}, \quad \mathbf{u}_\mathcal{C} = -\frac{k_\mathcal{C}}{\mu} \boldsymbol{\nabla} p_\mathcal{C}, \tag{1}$$

where $k_\mathcal{C}$ is the Darcy permeability of the choroidal tissue, μ is the dynamic viscosity of the IF and $p_\mathcal{C}(\theta)$ is the IF pressure in the choroid. Within the choroidal tissue, the volume of fluid entering from the CC per unit time per unit volume of tissue, $q_\mathcal{C}$, depends on the difference in albumin concentration between the blood and choroidal tissue, $c_{\mathcal{CC}} - c_\mathcal{C}$. According to Starling's law, these flows have both pressure-driven and osmotically-driven components due to albumin

concentrations:

$$q_{\mathcal{C}} = L_{\mathcal{CC}}\left(p_{\mathcal{CC}} - p_{\mathcal{C}} - \sigma_{\mathcal{CC}} R_g T (c_{\mathcal{CC}} - c_{\mathcal{C}})\right), \tag{2}$$

where $L_{\mathcal{CC}}$ is the hydraulic conductance of the vessels, $\sigma_{\mathcal{CC}}$ is the corresponding reflection coefficient, R_g is the ideal gas constant and T is the absolute temperature.

The purpose of this study is in part to assess the role of the SCS in the dynamics of the unconventional flow. Hence, in what follows we either assume the SCS is absent (which we term Case (i)), or that it is present and can be modelled as either a porous medium (which we term Case (ii)) or an open space filled with IF (which we term Case (iii)); note that we mainly assume Case (iii) in this paper.

In Case (i), where the SCS is absent (and $h_{\mathcal{P}} = 0$), the sclera and choroid share a common interface, while in Cases (ii) and (iii), the thickness of the SCS lying between the choroid and sclera depends on the local difference between the SCS pressure and that in the surroundings.

Since the sclera is assumed rigid, expansion or collapse of the SCS requires deformation of the internal compartments of the eye. As a first step, we assume that the choroid is entirely rigid and that the retina and vitreous can be lumped into one deformable component with pressure $p_{\mathcal{R}}$. The thickness of the SCS is then assumed to be determined by the pressure difference between the pressure in the SCS, $p_{\mathcal{P}}$, and the pressure in the retina, $p_{\mathcal{R}}$.

In this study we assume a constitutive law in the form

$$p_{\mathcal{P}} - p_{\mathcal{R}} = \lambda_{\mathcal{P}} \left(\frac{h_{\mathcal{P}}}{h_{\mathcal{P},\text{nat}}} - \left(\frac{h_{\mathcal{P}}}{h_{\mathcal{P},\text{nat}}} \right)^{-n} \right), \tag{3}$$

where $n \geq 0$ is an exponent, $h_{\mathcal{P},\text{nat}}(\theta)$ is the (nonuniform) thickness that the SCS would adopt if $p_{\mathcal{P}}$ and $p_{\mathcal{R}}$ are equal and $\lambda_{\mathcal{P}}$ is an elastic stiffness (which could be made a function of θ to reflect increased tethering towards the inlet). For $n = 0$ this law mimics a linearly elastic potential space. However, this assumption alone would mean that the height of the SCS could reach zero for some finite negative value of the transmural pressure difference. It is mathematically very challenging to have a collapsible channel that can transiently become completely occluded – the height of the space approaching zero is a singularity in the governing equations – and there is no clear way to allow the space to reopen again some time later. To prevent complete closure of the space we modify the linear constitutive law to include a penalty term with exponent $-n$, where $n > 0$; see plot in Figure S2. As the value of n decreases, the influence of this penalty term becomes increasingly localised to small channel widths; we use $n = 1$. With this model, the SCS can still become very close to zero height as the transmural pressure decreases, and by choosing a (small) threshold value one could still delineate between open and closed regions. Note that similar penalty terms are used in preventing complete collapse of veins in cardiovascular models.[1,2,3,4] As a further simplification, we assume that the tissue pressure in the retinal compartment $p_{\mathcal{R}}$ is fixed as the SCS thickness $h_{\mathcal{P}}$ changes.

In Case (ii), IF flow within the choroid is modelled as an incompressible Darcy flow with velocity vector $\mathbf{u}_{\mathcal{P}} = (u_{\mathcal{C}r}, u_{\mathcal{C}\theta}, u_{\mathcal{C}\phi})$ (flow rate per unit area) in spherical coordinates:

$$\boldsymbol{\nabla} \cdot \mathbf{u}_{\mathcal{P}} = 0, \quad \mathbf{u}_{\mathcal{P}} = -\frac{k_{\mathcal{P}}}{\mu} \boldsymbol{\nabla} p_{\mathcal{P}}. \tag{4}$$

In Case (iii), IF flow within the choroid is modelled as an incompressible Stokes flow with velocity vector $\mathbf{u}_{\mathcal{P}} = (u_{\mathcal{C}r}, u_{\mathcal{C}\theta}, u_{\mathcal{C}\phi})$ in spherical coordinates

$$\boldsymbol{\nabla} \cdot \mathbf{u}_{\mathcal{P}} = 0, \quad \boldsymbol{\nabla} p_{\mathcal{P}} = \mu \nabla^2 \mathbf{u}_{\mathcal{P}}. \tag{5}$$

We now specify boundary conditions between each layer. We will use $\hat{\mathbf{n}}_{ij}$ to indicate the unit normal vector pointing from region j to region i.

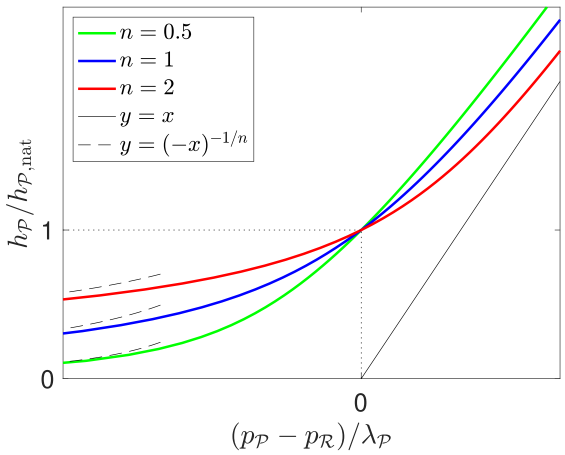

Figure S2: Sketch of the relationship between SCS pressure and thickness given by Equation (3) for the cases $n = 0.5$ (green), $n = 1$ (blue), $n = 2$ (red). The asymptotes at the left (dashed) and right (solid) of the graphs are shown in black.

Firstly, at the RPE (boundary between choroid and the retina, $r = R_0 - h_{\mathcal{P}} - h_{\mathcal{C}}$), we assume that IF flows at a prescribed rate

$$\hat{\mathbf{n}}_{\mathcal{R},\mathcal{C}} \cdot \mathbf{u}_{\mathcal{C}}\big|_{r=R_0-h_{\mathcal{P}}-h_{\mathcal{C}}} = -q_{\mathcal{R}}, \tag{6}$$

where $q_{\mathcal{R}}$ is the volume flow rate per unit surface area. The inward pointing unit normal $\hat{\mathbf{n}}_{\mathcal{R},\mathcal{C}}$ is given by

$$\hat{\mathbf{n}}_{\mathcal{R},\mathcal{C}} = -\frac{(R_0 - h_{\mathcal{P}} - h_{\mathcal{C}})\hat{\mathbf{e}}_r + (h'_{\mathcal{P}} + h'_{\mathcal{C}})\hat{\mathbf{e}}_\theta}{\sqrt{(R_0 - h_{\mathcal{P}} - h_{\mathcal{C}})^2 + (h'_{\mathcal{P}} + h'_{\mathcal{C}})^2}}. \tag{7}$$

Secondly, at the interface between the choroid and SCS, $r = R_0 - h_{\mathcal{P}}$, we impose continuity of normal stress. We assume the normal stresses of both the IF in the SCS and the fluid–porous medium complex are dominated by the IF pressures (which can be verifed *a posteriori*), giving the balance

$$p_{\mathcal{C}}\big|_{r=R_0-h_{\mathcal{P}}} = p_{\mathcal{P}}\big|_{r=R_0-h_{\mathcal{P}}}. \tag{8}$$

Additionally, we require continuity of fluid flux normal to the surface

$$\hat{\mathbf{n}}_{\mathcal{C},\mathcal{P}} \cdot \mathbf{u}_{\mathcal{C}}\big|_{r=R_0-h_{\mathcal{P}}} = \hat{\mathbf{n}}_{\mathcal{C},\mathcal{P}} \cdot \mathbf{u}_{\mathcal{P}}\big|_{r=R_0-h_{\mathcal{P}}}, \tag{9}$$

where the inward pointing normal $\hat{\mathbf{n}}_{\mathcal{C},\mathcal{P}}$ is given by

$$\hat{\mathbf{n}}_{\mathcal{C},\mathcal{P}} = -\frac{(R_0 - h_{\mathcal{P}})\hat{\mathbf{e}}_r + h'_{\mathcal{P}}\hat{\mathbf{e}}_\theta}{\sqrt{(R_0 - h_{\mathcal{P}})^2 + h'^2_{\mathcal{P}}}}. \tag{10}$$

At the scleral interface, in Case (i) we apply continuity of flux with the choroidal tissue so that

$$\hat{\mathbf{n}}_{\mathcal{C},\mathcal{S}} \cdot \mathbf{u}_{\mathcal{C}}\big|_{r=R_0} = -q_{\mathcal{S}}; \tag{11}$$

in Cases (ii) and (iii) we apply continuity of flux with the SCS so that

$$\hat{\mathbf{n}}_{\mathcal{P},\mathcal{S}} \cdot \mathbf{u}_{\mathcal{P}}\big|_{r=R_0} = -q_{\mathcal{S}}. \tag{12}$$

The IF flow across the sclera is modelled by the Starling equation:

$$q_{\mathcal{S}} = \frac{k_{\mathcal{S}}}{\mu h_{\mathcal{S}}}\left(p_{\mathcal{P}}\big|_{r=R_0} - p_{\mathcal{O}} - \sigma_{\mathcal{S}} R_g T (c_{\mathcal{P}}\big|_{r=R_0} - c_{\mathcal{S}})\right), \tag{13}$$

where $k_{\mathcal{S}}$ is the hydraulic conductivity of the sclera and $\sigma_{\mathcal{S}}$ is the reflection coefficient of albumin (equivalently, this is modeled as unidirectional Darcy flow in the scleral tissue).

When we model the SCS as open (Case (iii)), we require additional boundary conditions. We denote the tangential vector $\hat{\mathbf{t}}_{ij}$ between region i and j directed from the pole towards the iris root. The equations for no slip at the two boundaries of the SCS are given by

$$\hat{\mathbf{t}}_{\mathcal{P},\mathcal{S}} \cdot \mathbf{u}_{\mathcal{P}}\big|_{r=R_0} = 0, \quad \hat{\mathbf{t}}_{\mathcal{C},\mathcal{P}} \cdot \mathbf{u}_{\mathcal{P}}\big|_{r=R_0-h_{\mathcal{P}}} = 0, \tag{14}$$

where the tangent vectors are given by

$$\hat{\mathbf{t}}_{\mathcal{P},\mathcal{S}} = \hat{\mathbf{e}}_\theta, \quad \hat{\mathbf{t}}_{\mathcal{C},\mathcal{P}} = \frac{-h'_{\mathcal{P}}\hat{\mathbf{e}}_r + (R_0 - h_{\mathcal{P}})\hat{\mathbf{e}}_\theta}{\sqrt{(R_0 - h_{\mathcal{P}})^2 + h'^2_{\mathcal{P}}}}. \tag{15}$$

We also consider the more general Beavers–Joseph condition at these two interfaces in Section 6

Finally, we set boundary conditions on all layers at the pole and iris root. We assume the tissue between the anterior chamber and iris root has resistance R_{icm}, leading to the boundary condition

$$\mathrm{IOP} - p|_{\theta=\theta_0} = R_{icm}\, Q|_{\theta=\theta_0}\,, \tag{16}$$

where Q is the volume flow rate through the tissue. Symmetry at the posterior pole $\theta = 0$ requires two conditions

$$\mathbf{u}_{\mathcal{C}} \cdot \hat{\mathbf{e}}_\theta|_{\theta=0} = \mathbf{u}_{\mathcal{P}} \cdot \hat{\mathbf{e}}_\theta|_{\theta=0} = 0. \tag{17}$$

2.2 Albumin transport

We now determine the governing equations for the albumin that is dissolved in the IF. These equations complement those found in the previous section for IF flow. Assuming a dilute solution, we model transport in the choroidal tissue and SCS using steady diffusion-convection equations in the form

$$\boldsymbol{\nabla} \cdot \mathbf{j}_{\mathcal{C}} = s_{\mathcal{C}}, \qquad \mathbf{j}_{\mathcal{C}} = \mathbf{u}_{\mathcal{C}} c_{\mathcal{C}} - D_{\mathcal{C}} \boldsymbol{\nabla} c_{\mathcal{C}}\,, \tag{18}$$

$$\boldsymbol{\nabla} \cdot \mathbf{j}_{\mathcal{P}} = 0, \qquad \mathbf{j}_{\mathcal{P}} = \mathbf{u}_{\mathcal{P}} c_{\mathcal{P}} - D_{\mathcal{P}} \boldsymbol{\nabla} c_{\mathcal{P}}\,, \tag{19}$$

where $c_{\mathcal{I}}$ is albumin concentration, $\mathbf{j}_{\mathcal{I}}$ is flux in region $\mathcal{I}$, and $D_{\mathcal{I}}$ is the diffusion coefficient. For simplicity, we assume the diffusion coefficients are equal in the choroidal tissue and SCS, so that $D_{\mathcal{C}} = D_{\mathcal{P}} = D$ (although the analysis can be made to work if these are not equal). The choroidal system is supplied by a continuous source of IF from the choroidal vasculature, given by $s_{\mathcal{C}}$ moles of albumin entering the choroidal tissues per unit volume.

On the outer boundary of the SCS ($r = R_0$, or in Case (i) this is the outer boundary of the choroid), we assume a flux $s_{\mathcal{S}}$ of albumin passes through the sclera in the form

$$\hat{\mathbf{n}}_{\mathcal{P},\mathcal{S}} \cdot \mathbf{j}_{\mathcal{P}}|_{r=R_0} = s_{\mathcal{S}}. \tag{20}$$

We also assume that the RPE is impermeable to albumin, so that

$$\hat{\mathbf{n}}_{\mathcal{R},\mathcal{C}} \cdot \mathbf{j}_{\mathcal{C}}|_{r=R_0-h_{\mathcal{P}}-h_{\mathcal{C}}} = 0. \tag{21}$$

At the interface between the choroid and SCS ($r = R_0 - h_{\mathcal{P}}$), we assume continuity of concentration and normal flux, so that

$$c_{\mathcal{C}}|_{r=R_0-h_{\mathcal{P}}} = c_{\mathcal{P}}|_{r=R_0-h_{\mathcal{P}}}\,, \quad \hat{\mathbf{n}}_{\mathcal{C},\mathcal{P}} \cdot \mathbf{j}_{\mathcal{C}}|_{r=R_0-h_{\mathcal{P}}} = \hat{\mathbf{n}}_{\mathcal{C},\mathcal{P}} \cdot \mathbf{j}_{\mathcal{P}}|_{r=R_0-h_{\mathcal{P}}}\,. \tag{22}$$

Albumin flux across the blood vessel wall is modeled using the Kedem–Katchalsky equation (scaled per unit volume) for solute transport:[5,6,7]

$$s_{\mathcal{C}} = \beta_{\mathcal{CC}}(c_{\mathcal{CC}} - c_{\mathcal{C}}) + \frac{c_{\mathcal{C}} + c_{\mathcal{CC}}}{2}(1 - \sigma_{\mathcal{CC}})q_{\mathcal{CC}}, \tag{23}$$

where $\beta_{\mathcal{CC}}$ is the permeation coefficient of the CC per unit volume multiplied by R_gT (also referred to as albumin conductance), and $\sigma_{\mathcal{CC}}$ is the reflection coefficient of the CC walls. The IF flux $q_{\mathcal{CC}}$ is given by (2). In this equation, the first term accounts for transcytosis plus any other diffusive processes present, while the second term accounts for the advective flow of albumin (expected to be directed into the CC).

Albumin flux across the sclera is modelled using the Patlak equation:[8]

$$s_{\mathcal{S}} = q_{\mathcal{S}}(1 - \sigma_{\mathcal{S}})\frac{c_{\mathcal{O}} - c_{\mathcal{P}}|_{r=R_0}e^{Pe_{\mathcal{S}}}}{1 - e^{Pe_{\mathcal{S}}}}, \tag{24}$$

with $Pe_{\mathcal{S}} = q_{\mathcal{S}}(1 - \sigma_{\mathcal{S}})/\beta_{\mathcal{S}}$. Here $\beta_{\mathcal{S}}$ is the permeation coefficient per unit area of the sclera multiplied by R_gT, which we term the albumin conductance, $\sigma_{\mathcal{S}}$ is the reflection coefficient and $c_{\mathcal{O}}$ is the orbital concentration. Moreover, $q_{\mathcal{S}}$ is the IF velocity across the sclera, given by Equation (13). We note that for small $Pe_{\mathcal{S}}$, the Patlak equation reduces to the Kedem–Katchalsky equation, and the expression for the flux would be similar to that for the CC.

Finally, we set boundary conditions at the edges of all the layers at the pole and iris root. Under the assumption that albumin in the ciliary body region is well mixed, we prescribe boundary conditions on the concentration at the iris root in the form

$$\left.\frac{\partial c_{\mathcal{C}}}{\partial \theta}\right|_{\theta=\theta_0} = \left.\frac{\partial c_{\mathcal{P}}}{\partial \theta}\right|_{\theta=\theta_0} = 0. \tag{25}$$

Finally, we impose symmetry conditions at the posterior pole for continuity of the diffusive flux of albumin in the form:

$$\left.\frac{\partial c_{\mathcal{C}}}{\partial \theta}\right|_{\theta=0} = \left.\frac{\partial c_{\mathcal{P}}}{\partial \theta}\right|_{\theta=0} = 0. \tag{26}$$

3 Simplification of IF flow equations for small thickness of the domain

In order to reduce the complexity of the governing equations systematically, we exploit the observation that the choroidal and SCS domains are both long compared to their typical width, assuming that $\epsilon_{\mathcal{C}} = h_{\mathcal{C}0}/R_0 \ll 1$, $\epsilon_{\mathcal{P}} = h_{\mathcal{P}0}/R_0 \ll 1$, where $h_{\mathcal{C}0}$ and $h_{\mathcal{P}0}$ are representative values of $h_{\mathcal{C}}$ and $h_{\mathcal{P}}$, respectively.

In Section 3.1 we apply an appropriate scaling to the geometry of the choroidal tissue, and find the leading-order solution; this analysis spans all three cases for modelling the SCS. In Sections 3.2–3.4, we close the problem; in Section 3.2, we consider Case (i) and apply the boundary condition at the inner scleral surface; in Sections 4.3 and 4.4, we consider Cases (ii) and (iii), respectively, and solve for the flow in the SCS as well as applying the boundary conditions at the inner scleral surface.

3.1 Flow in the choroidal tissue

In accordance with the assumpation $\epsilon_{\mathcal{C}} \ll 1$, mass conservation in the choroidal tissue dictates that flow velocities in the radial direction must be much less than velocities in the angular

direction. Therefore, the continuity equation in the choroidal tissue (1a) suggests that $u_{\mathcal{C}r}$ scales as $\epsilon_{\mathcal{C}} U_{\mathcal{C}}$, where $U_{\mathcal{C}}$ is the azimuthal velocity scale for $u_{\mathcal{C}\theta}$ and $\epsilon_{\mathcal{C}}$ is small.

We therefore non-dimensionalise the flow variables in the choroidal tissue according to

$$r = R_0 - h_{\mathcal{C}0} r^*, \quad h_{\mathcal{C}} = h_{\mathcal{C}0} h_{\mathcal{C}}^*, \quad p_{\mathcal{C}} = P_0\, p_{\mathcal{C}}^*, \tag{27}$$

$$u_{\mathcal{C}r} = \epsilon_{\mathcal{C}} U_{\mathcal{C}} u_{\mathcal{C}r}^*, \quad u_{\mathcal{C}\theta} = U_{\mathcal{C}} u_{\mathcal{C}\theta}^*, \quad q_{\mathcal{R}} = \epsilon_{\mathcal{C}} U_{\mathcal{C}} q_{\mathcal{R}}^*, \quad q_{\mathcal{C}} = q_0 q_{\mathcal{C}}^*, \tag{28}$$

where $U_{\mathcal{C}} = Q_0/(2\pi R_0 h_{\mathcal{C}0})$ and Q_0 is 10% of the total aqueous flow, $Q_0 = 0.25\ \mu$l/min, with 10% being taken as a nominal scale for the physiological unconventional flow. Balancing the θ-component of the Darcy equation (1b) suggests the scale $P_0 = \mu R_0 U_{\mathcal{C}}/k_{\mathcal{C}} = \mu Q_0/(2\pi h_{\mathcal{C}0} k_{\mathcal{C}})$, while balancing the continuity equation (1a) suggests $q_0 = U_{\mathcal{C}}/R_0 = Q_0/(2\pi R_0^2 h_{\mathcal{C}0})$.

In Section 2.1, to obtain Eq. (8), we assumed the normal stresses were dominated by IF pressure and we will now check the scaling at the choroid–SCS interface. Within the choroidal tissue the IF stress is given by

$$\sigma_{\mathcal{C}} = -p_{\mathcal{C}} \mathbf{I} + \mu(\boldsymbol{\nabla}\mathbf{v} + \boldsymbol{\nabla}\mathbf{v}^T), \tag{29}$$

where $\mathbf{v}$ is the IF velocity vector and $\mathbf{I}$ is the identity matrix. Since $\mathbf{v}$ has magnitude of order $U_{\mathcal{C}}/\phi$, where ϕ is the porosity of the choroidal tissue, the deviatoric stress has a magnitude of order $\mu U_{\mathcal{C}}/(\phi h_{\mathcal{C}0}) = k_{\mathcal{C}}\, P_0/(\phi R_0 h_{\mathcal{C}0})$. For the parameter values given in Table 1 in the main paper, this is approximately $4 \cdot 10^{-9} P_0/\phi$, which is much smaller than P_0 as long as $\phi \gg 4 \cdot 10^{-9}$ (which we assume to be the case). This means the normal stress is dominated by the IF pressure over the whole surface, as already assumed.

Using the non-dimensionalisation, the governing equations (1) and boundary conditions (6) become, respectively,

$$-\frac{1}{(1-\epsilon_{\mathcal{C}} r^*)^2}\frac{\partial}{\partial r^*}(1-\epsilon_{\mathcal{C}} r^*)^2 u_{\mathcal{C}r}^* + \frac{1}{(1-\epsilon_{\mathcal{C}} r^*)\sin\theta}\frac{\partial}{\partial\theta} u_{\mathcal{C}\theta}^* \sin\theta = q_{\mathcal{C}}^*, \tag{30}$$

$$u_{\mathcal{C}r}^* = \frac{1}{\epsilon_{\mathcal{C}}^2}\frac{\partial p_{\mathcal{C}}^*}{\partial r^*}, \quad u_{\mathcal{C}\theta}^* = -\frac{1}{1-\epsilon_{\mathcal{C}} r^*}\frac{\partial p_{\mathcal{C}}^*}{\partial\theta}, \tag{31}$$

$$\left.\frac{(1-\epsilon_{\mathcal{C}}(h_{\mathcal{P}}^* + h_{\mathcal{C}}^*))u_{\mathcal{C}r}^* + (h_{\mathcal{P}}'^* + h_{\mathcal{C}}'^*)u_{\mathcal{C}\theta}^*}{\sqrt{(1-\epsilon_{\mathcal{C}}(h_{\mathcal{P}}^* + h_{\mathcal{C}}^*))^2 + \epsilon_{\mathcal{C}}^2(h_{\mathcal{P}}'^* + h_{\mathcal{C}}'^*)^2}}\right|_{r^* = h_{\mathcal{P}}^* + h_{\mathcal{C}}^*} = q_{\mathcal{R}}^*. \tag{32}$$

Eq. (31) implies that r^*-dependent variations of $p_{\mathcal{C}}^*$ occur at $O(\epsilon_{\mathcal{C}}^2)$. Neglecting corrections of $O(\epsilon_{\mathcal{C}})$, we solve (30), using (31) and (32) to obtain

$$u_{\mathcal{C}r}^* = \frac{1}{\sin\theta}\frac{\partial}{\partial\theta}\left((h_{\mathcal{P}}^* + h_{\mathcal{C}}^* - r^*)\frac{\partial p_{\mathcal{C}}^*}{\partial\theta}\sin\theta\right) + q_{\mathcal{C}}^*(h_{\mathcal{P}}^* + h_{\mathcal{C}}^* - r^*) + q_{\mathcal{R}}^*, \tag{33}$$

where we have assumed that $c_{\mathcal{C}}$ is independent of r to leading order (to be shown *a posteriori*). Redimensionalising, we derive

$$u_{\mathcal{C}r} = \frac{k_{\mathcal{C}}}{\mu R_0^2 \sin\theta}\frac{\partial}{\partial\theta}\left((r + h_{\mathcal{P}} + h_{\mathcal{C}} - R_0)\frac{\partial p_{\mathcal{C}}}{\partial\theta}\sin\theta\right) + q_{\mathcal{C}}(r + h_{\mathcal{P}} + h_{\mathcal{C}} - R_0) + q_{\mathcal{R}}, \tag{34}$$

and

$$u_{\mathcal{C}\theta} = -\frac{k_{\mathcal{C}}}{\mu R_0}\frac{\partial p_{\mathcal{C}}}{\partial\theta}. \tag{35}$$

The remainder of the analysis of the flow depends on the particular case considered; Case (i) is considered in Section 3.2, Case (ii) in Section 3.3 and Case (iii) in Section 3.4.

3.2 Flow in Case (i)

We apply the boundary condition at the interface with the sclera (13) to derive the mass conservation equation

$$\frac{k_{\mathcal{C}}}{\mu R_0^2 \sin\theta} \frac{\mathrm{d}}{\mathrm{d}\theta}\left(h_{\mathcal{C}} \frac{\mathrm{d}p}{\mathrm{d}\theta} \sin\theta\right) + h_{\mathcal{C}} q_{\mathcal{C}} + q_{\mathcal{R}} = q_{\mathcal{S}}, \tag{36}$$

where we have set $p_{\mathcal{C}} = p$. The total flow rate is a function of position around the eye, given by

$$Q = \int_{R_0 - h_{\mathcal{C}}}^{R_0} 2\pi r \sin\theta \left(-u_{\mathcal{C}\theta}\right) \mathrm{d}r = \frac{2\pi k_{\mathcal{C}} h_{\mathcal{C}}}{\mu} \sin\theta \frac{\mathrm{d}p}{\mathrm{d}\theta}, \tag{37}$$

with the unconventional flow given by $Q(\theta_0)$; rearranging Eq. (36) and Eq. (37) gives

$$\frac{\mathrm{d}p}{\mathrm{d}\theta} = \frac{\mu Q}{2\pi k_{\mathcal{C}} h_{\mathcal{C}} \sin\theta}, \tag{38}$$

$$\frac{\mathrm{d}Q}{\mathrm{d}\theta} = -\, 2\pi R_0^2 \sin\theta \left(h_{\mathcal{C}} q_{\mathcal{C}} + q_{\mathcal{R}} - q_{\mathcal{S}}\right), \tag{39}$$

with conditions Eq. (16) and Eq. (17) giving $Q = 0$ at $\theta = 0$ and $\mathrm{IOP} - p = R_{icm} Q$ at $\theta = \theta_0$.

3.3 Analysis of flow in Case (ii)

In Cases (ii) and (iii), we additionally need to analyse the IF flow in the SCS. In Case (ii), this is also treated as a porous medium, and we analyse this in Section 3.3.1 and apply the boundary condition in Section 3.3.2.

3.3.1 Flow in SCS in Case (ii)

Scaling the flow variables in the choroidal tissue as in Section 3.1, we impose additional scalings on the flow in the SCS such that

$$h_{\mathcal{P}} = h_{\mathcal{C}0} h_{\mathcal{P}}^*, \quad p_{\mathcal{P}} = P_0\, p_{\mathcal{P}}^*,$$
$$u_{\mathcal{P}r} = \epsilon_{\mathcal{C}} U_{\mathcal{C}} u_{\mathcal{P}r}^*, \quad u_{\mathcal{P}\theta} = U_{\mathcal{C}} u_{\mathcal{P}\theta}^*, \quad q_{\mathcal{S}} = \epsilon_{\mathcal{C}} U_{\mathcal{C}} q_{\mathcal{S}}^*. \tag{40}$$

In this case the governing equations (4) and boundary conditions (13) become,

$$-\frac{1}{(1-\epsilon_{\mathcal{C}} r^*)^2} \frac{\partial}{\partial r^*} (1-\epsilon_{\mathcal{C}} r^*)^2 u_{\mathcal{P}r}^* + \frac{1}{(1-\epsilon_{\mathcal{C}} r^*)\sin\theta} \frac{\partial}{\partial\theta} u_{\mathcal{P}\theta}^* \sin\theta = 0, \tag{41}$$

$$u_{\mathcal{P}r}^* = \frac{k_{\mathcal{P}}^*}{\epsilon_{\mathcal{C}}^2} \frac{\partial p_{\mathcal{P}}^*}{\partial r^*}, \quad u_{\mathcal{P}\theta}^* = -\frac{k_{\mathcal{P}}^*}{1-\epsilon_{\mathcal{C}} r^*} \frac{\partial p_{\mathcal{P}}^*}{\partial\theta}, \tag{42}$$

$$u_{\mathcal{P}r}^*|_{r^*=0} = q_{\mathcal{S}}^*, \tag{43}$$

where $k_{\mathcal{P}}^* = k_{\mathcal{P}}/k_{\mathcal{C}}$. To leading order in $\epsilon_{\mathcal{C}}$, we derive

$$u_{\mathcal{P}r}^* = -\frac{k_{\mathcal{P}}^* r^*}{\sin\theta} \frac{\partial}{\partial\theta}\left(\frac{\partial p_{\mathcal{P}}^*}{\partial\theta} \sin\theta\right) + q_{\mathcal{S}}^*, \tag{44}$$

and, redimensionalising, we obtain

$$u_{\mathcal{P}r} = \frac{(r-R_0) k_{\mathcal{P}}}{\mu R_0^2 \sin\theta} \frac{\partial}{\partial\theta}\left(\frac{\partial p_{\mathcal{P}}}{\partial\theta} \sin\theta\right) + q_{\mathcal{S}}, \quad u_{\mathcal{P}\theta} = -\frac{k_{\mathcal{P}}}{\mu R_0} \frac{\partial p_{\mathcal{P}}}{\partial\theta}. \tag{45}$$

3.3.2 Matching conditions for flow in Case (ii)

The interstitial flud flows in the SCS (governed by (45)) and in the choroidal tissue (governed by (34)) must be matched across the interface. The interface conditions are given by Eqs. (8) and (9). Eq. (8) implies $p_{\mathcal{C}} = p_{\mathcal{P}} = p$ and so at leading order (9) becomes

$$u_{\mathcal{C}r} + \frac{1}{R_0} h'_{\mathcal{P}} u_{\mathcal{C}\theta}\bigg|_{r=R_0-h_{\mathcal{P}}} = u_{\mathcal{P}r} + \frac{1}{R_0} h'_{\mathcal{P}} u_{\mathcal{P}\theta}\bigg|_{r=R_0-h_{\mathcal{P}}}, \tag{46}$$

which gives

$$\frac{1}{\mu R_0^2 \sin\theta}\frac{\mathrm{d}}{\mathrm{d}\theta}\left((k_{\mathcal{P}}h_{\mathcal{P}} + k_{\mathcal{C}}h_{\mathcal{C}})\frac{\mathrm{d}p}{\mathrm{d}\theta}\sin\theta\right) + h_{\mathcal{C}}q_{\mathcal{C}} + q_{\mathcal{R}} - q_{\mathcal{S}} = 0, \tag{47}$$

which is to be solved together with conditions (16), (17).

The total flow rate is a function of the distance around the eye:

$$Q = \int_{R_0-h_{\mathcal{P}}-h_{\mathcal{C}}}^{R_0-h_{\mathcal{P}}} 2\pi r\sin\theta\,(-u_{\mathcal{C}\theta})\;\mathrm{d}r + \int_{R_0-h_{\mathcal{P}}}^{R_0} 2\pi r\sin\theta\,(-u_{\mathcal{P}\theta})\;\mathrm{d}r = \frac{2\pi}{\mu}(k_{\mathcal{C}}h_{\mathcal{C}} + k_{\mathcal{P}}h_{\mathcal{P}})\frac{\mathrm{d}p}{\mathrm{d}\theta}\sin\theta, \tag{48}$$

with the unconventional flow given by $Q(\theta_0)$, and hence

$$\frac{\mathrm{d}p}{\mathrm{d}\theta} = \frac{\mu Q}{2\pi\sin\theta(k_{\mathcal{C}}h_{\mathcal{C}} + k_{\mathcal{P}}h_{\mathcal{P}})}, \tag{49}$$

$$\frac{\mathrm{d}Q}{\mathrm{d}\theta} = -\,2\pi R_0^2\sin\theta\,(h_{\mathcal{C}}q_{\mathcal{C}} + q_{\mathcal{R}} - q_{\mathcal{S}})\,, \tag{50}$$

with boundary conditions (16), (17) giving $Q = 0$ at $\theta = 0$ and $\mathrm{IOP} - p = R_{icm}Q$ at $\theta = \theta_0$.

3.4 Analysis of flow in Case (iii)

In Case (ii), the SCS is open, and we analyse the flow in Section 3.4.1 and apply the boundary condition in Section 3.4.2.

3.4.1 Flow in SCS in Case (iii)

We impose alternative scalings on the variables in the SCS (different from those in the choroid) in the form

$$r = R_0 - h_{\mathcal{P}0}r^\dagger, \quad h_{\mathcal{P}} = h_{\mathcal{P}0}h_{\mathcal{P}}^\dagger, \quad p_{\mathcal{P}} = P_0 p_{\mathcal{P}}^\dagger, \tag{51}$$

$$u_{\mathcal{P}r} = \epsilon_{\mathcal{P}}U_{\mathcal{P}}u_{\mathcal{P}r}^\dagger, \quad u_{\mathcal{P}\theta} = U_{\mathcal{P}}u_{\mathcal{P}\theta}^\dagger, \quad q_{\mathcal{S}} = \epsilon_{\mathcal{P}}U_{\mathcal{P}}q_{\mathcal{S}}^\dagger; \tag{52}$$

note that the scale for the pressure in the SCS, P_0, is the same as that in the choroidal tissue, as these two regions share an interface where the pressures are equal (and the primary pressure drop in these regions is expected to be parallel to this interface). Balancing the viscous drag in the θ-component of Eq. (5b) in Case (ii) yields $U_{\mathcal{P}} = \epsilon_{\mathcal{P}}^2 R_0 P_0/\mu = h_{\mathcal{P}0}^2 U_{\mathcal{C}}/k_{\mathcal{C}}$.

In a similar way to Section 3.1, we verify our earlier assumption that the normal stresses are dominated by IF pressure at the choroid–SCS interface (see Section 2.1, Eq. (8)). In this case, the IF stress is given by $\sigma_{\mathcal{P}} = -p_{\mathcal{P}}I + \mu(\boldsymbol{\nabla}\mathbf{u}_{\mathcal{P}} + \boldsymbol{\nabla}\mathbf{u}_{\mathcal{P}}^T)$, so that the deviatoric component of the normal stress at the interface is of order $\mu\epsilon_{\mathcal{P}}U_{\mathcal{P}}/h_{\mathcal{P}0} = \epsilon_{\mathcal{P}}^2 P_0$, meaning that the normal stress is also dominated by the IF pressure, consistent with our earlier assumption.

Using the non-dimensionalisation, we can now simplify our model equations. In Case (iii), the governing equations (5) and boundary conditions (13) and (14) become, respectively,

$$-\frac{1}{(1-\epsilon_{\mathcal{P}}r^\dagger)^2}\frac{\partial}{\partial r^\dagger}\left((1-\epsilon_{\mathcal{P}}r^\dagger)^2 u_{\mathcal{P}r}^\dagger\right) + \frac{1}{(1-\epsilon_{\mathcal{P}}r^\dagger)\sin\theta}\frac{\partial}{\partial\theta}\left(u_{\mathcal{P}\theta}^\dagger\sin\theta\right) = 0, \tag{53}$$

$$\frac{\partial p^\dagger_{\mathcal{P}}}{\partial r^\dagger} = -\frac{\epsilon^2_{\mathcal{P}}}{(1-\epsilon_{\mathcal{P}} r^\dagger)^2}\frac{\partial}{\partial r^\dagger}\left((1-\epsilon_{\mathcal{P}} r^\dagger)^2\frac{\partial u^\dagger_{\mathcal{P}r}}{\partial r^\dagger}\right) - \frac{\epsilon^4_{\mathcal{P}}}{(1-\epsilon_{\mathcal{P}} r^\dagger)^2}\frac{\partial}{\partial \theta}\left(\sin\theta\frac{\partial u^\dagger_{\mathcal{P}r}}{\partial \theta}\right)$$

$$+\frac{2\epsilon^4_{\mathcal{P}}}{(1-\epsilon_{\mathcal{P}} r^\dagger)^2}u^\dagger_{\mathcal{P}r} + \frac{2\epsilon^3_{\mathcal{P}}}{(1-\epsilon_{\mathcal{P}} r^\dagger)^2\sin\theta}\frac{\partial}{\partial\theta}\left(u^\dagger_{\mathcal{P}\theta}\sin\theta\right), \tag{54}$$

$$\frac{\partial p^\dagger_{\mathcal{P}}}{\partial \theta} = \frac{1}{1-\epsilon_{\mathcal{P}} r^\dagger}\left(\frac{\partial}{\partial r^\dagger}\left((1-\epsilon_{\mathcal{P}} r^\dagger)^2\frac{\partial u^\dagger_{\mathcal{P}\theta}}{\partial r^\dagger}\right) + \frac{\epsilon^2_{\mathcal{P}}}{\sin\theta}\frac{\partial}{\partial\theta}\left(\sin\theta\frac{\partial u^\dagger_{\mathcal{P}\theta}}{\partial\theta}\right) - \frac{\epsilon^2_{\mathcal{P}} u^\dagger_{\mathcal{P}\theta}}{\sin^2\theta} + 2\epsilon^3_{\mathcal{P}}\frac{\partial u^\dagger_{\mathcal{P}r}}{\partial\theta}\right), \tag{55}$$

$$u^\dagger_{\mathcal{P}r}\Big|_{r^\dagger=0} = q^\dagger_{\mathcal{S}}, \quad u^\dagger_{\mathcal{P}\theta}\Big|_{r^\dagger=0} = 0, \quad \left(-\epsilon^2_{\mathcal{P}} h'^\dagger_{\mathcal{P}} u^\dagger_{\mathcal{P}r} + (1-\epsilon_{\mathcal{P}} h^\dagger_{\mathcal{P}})u^\dagger_{\mathcal{P}\theta}\right)\Big|_{r^\dagger=h^\dagger_{\mathcal{P}}} = 0. \tag{56}$$

Neglecting terms of $O(\epsilon_{\mathcal{P}})$, $p^\dagger_{\mathcal{P}}$ is independent of $r^\dagger$ and

$$u^\dagger_{\mathcal{P}\theta} = -\frac{1}{2}r^\dagger(h^\dagger_{\mathcal{P}} - r^\dagger)\frac{\partial p^\dagger_{\mathcal{P}}}{\partial\theta}, \tag{57}$$

$$u^\dagger_{\mathcal{P}r} = -\frac{r^{\dagger 2}}{12\sin\theta}\frac{\partial}{\partial\theta}\left((3h^\dagger_{\mathcal{P}} - 2r^\dagger)\frac{\partial p^\dagger_{\mathcal{P}}}{\partial\theta}\sin\theta\right) + q^\dagger_{\mathcal{S}}. \tag{58}$$

Re-dimensionalising,

$$u_{\mathcal{P}\theta} = -\frac{1}{2\mu R_0}(R_0 - r)(r - (R_0 - h_{\mathcal{P}}))\frac{\partial p_{\mathcal{P}}}{\partial\theta}, \tag{59}$$

$$u_{\mathcal{P}r} = -\frac{(R_0 - r)^2}{6\mu R_0^2\sin\theta}\frac{\partial}{\partial\theta}\left(\left(r - \left(R_0 - \frac{3}{2}h_{\mathcal{P}}\right)\right)\frac{\partial p_{\mathcal{P}}}{\partial\theta}\sin\theta\right) + q_{\mathcal{S}}. \tag{60}$$

3.4.2 Matching conditions for flow in Case (iii)

Matching flow across the interface between the SCS and the choroid, the interface conditions (8) and (9) give $p_{\mathcal{C}} = p_{\mathcal{P}} = p$ and Eq. (46). Eq. (46) implies

$$\frac{1}{\mu R_0^2\sin\theta}\frac{\mathrm{d}}{\mathrm{d}\theta}\left(\left(k_{\mathcal{C}}h_{\mathcal{C}}\frac{\mathrm{d}p}{\mathrm{d}\theta} + \frac{1}{12}h^3_{\mathcal{P}}\frac{\mathrm{d}p}{\mathrm{d}\theta}\right)\sin\theta\right) + h_{\mathcal{C}}q_{\mathcal{C}} + q_{\mathcal{R}} - q_{\mathcal{S}} = 0, \tag{61}$$

where we have used Eq. (8) to set $p_{\mathcal{C}} = p_{\mathcal{P}} = p$. In non-dimensional terms, we have that

$$\frac{1}{\sin\theta}\frac{\mathrm{d}}{\mathrm{d}\theta}\left(\left(Ch^*_{\mathcal{C}}\frac{\mathrm{d}p^*}{\mathrm{d}\theta} + \frac{1}{12}h^{\dagger 3}_{\mathcal{P}}\frac{\mathrm{d}p^\dagger}{\mathrm{d}\theta}\right)\sin\theta\right) + Ch^*_{\mathcal{C}}q^*_{\mathcal{C}} + Cq^*_{\mathcal{R}} - q^\dagger_{\mathcal{S}} = 0, \tag{62}$$

where $C = h_{\mathcal{C}0}k_{\mathcal{C}}/h^3_{\mathcal{P}0}$ (note that two different sets of nondimensional scales appear in Eq. (62)). We require C to be $O(1)$; in fact, for C to lie between 0.1 and 10, we need $h_{\mathcal{P}0}$ to be between around 0.7 and 3 μm. Equation (62) is to be solved together with conditions (16), (17).

The total flow rate as a function of theta is given by

$$Q = \int_{R_0-h_{\mathcal{P}}-h_{\mathcal{C}}}^{R_0-h_{\mathcal{P}}} 2\pi r\sin\theta\,(-u_{\mathcal{C}\theta})\,\mathrm{d}r + \int_{R_0-h_{\mathcal{P}}}^{R_0} 2\pi r\sin\theta\,(-u_{\mathcal{P}\theta})\,\mathrm{d}r,$$

$$= \frac{2\pi\sin\theta}{\mu}\left(k_{\mathcal{C}}h_{\mathcal{C}}\frac{\mathrm{d}p}{\mathrm{d}\theta} + \frac{1}{12}h^3_{\mathcal{P}}\frac{\mathrm{d}p}{\mathrm{d}\theta}\right), \tag{63}$$

with the unconventional flow given by $Q(\theta_0)$, and hence

$$\frac{\mathrm{d}p}{\mathrm{d}\theta} = \frac{\mu Q}{2\pi\sin\theta(k_{\mathcal{C}}h_{\mathcal{C}} + h^3_{\mathcal{P}}/12)}, \tag{64}$$

$$\frac{\mathrm{d}Q}{\mathrm{d}\theta} = -2\pi R_0^2\sin\theta\left(h_{\mathcal{C}}q_{\mathcal{C}} + q_{\mathcal{R}} - q_{\mathcal{S}}\right), \tag{65}$$

with conditions (16), (17) giving $Q = 0$ at $\theta = 0$ and $\mathrm{IOP} - p = R_{icm}Q$ at $\theta = \theta_0$.

4 Simplification of albumin transport equations for small thickness of the domain

In a similar manner to Section 3, we derive reduced equations for the albumin transport in the choroidal tissue and SCS. As for IF flow, in Section 4.1, we solve for the leading-order transport in the choroidal tissue, and, in Sections 4.2–4.4, we close the problem in Cases (i)–(iii) by solving in the SCS, as appropriate, and applying the boundary condition at the sclera.

4.1 Analysis of transport in the choroidal tissue

We scale albumin concentration with $c_0 = c_{\mathcal{CC}}$, the concentration in CC plasma. We therefore set

$$c_{\mathcal{C}} = c_0 c_{\mathcal{C}}^*, \quad j_{\mathcal{C}r} = \epsilon_{\mathcal{C}} U_{\mathcal{C}} c_0 j_{\mathcal{C}r}^*, \quad j_{\mathcal{C}\theta} = U_{\mathcal{C}} c_0 j_{\mathcal{C}\theta}^*, \quad s_{\mathcal{C}} = s_0 s_{\mathcal{C}}^*, \tag{66}$$

and balancing Eq. (18a) gives $s_0 = U_{\mathcal{C}} c_0 / R_0$.

Eqs. (18) and boundary conditions (21b) become, respectively,

$$-\frac{1}{(1-\epsilon_{\mathcal{C}} r^*)^2}\frac{\partial}{\partial r^*}(1-\epsilon_{\mathcal{C}} r^*)^2 j_{\mathcal{C}r}^* + \frac{1}{(1-\epsilon_{\mathcal{C}} r^*)\sin\theta}\frac{\partial}{\partial\theta} j_{\mathcal{C}\theta}^* \sin\theta = s_{\mathcal{C}}^*, \tag{67}$$

$$j_{\mathcal{C}r}^* = \frac{1}{\epsilon_{\mathcal{C}}^2}\left(c_{\mathcal{C}}^*\frac{\partial p_{\mathcal{C}}^*}{\partial r^*} + \frac{1}{\mathrm{Pe}_{\mathcal{C}}}\frac{\partial c_{\mathcal{C}}^*}{\partial r^*}\right), \quad j_{\mathcal{C}\theta}^* = -\frac{1}{1-\epsilon_{\mathcal{C}} r^*}\left(c_{\mathcal{C}}^*\frac{\partial p_{\mathcal{C}}^*}{\partial\theta} + \frac{1}{\mathrm{Pe}_{\mathcal{C}}}\frac{\partial c_{\mathcal{C}}^*}{\partial\theta}\right), \tag{68}$$

$$\left.\left((1-\epsilon_{\mathcal{C}}(h_{\mathcal{P}}^* + h_{\mathcal{C}}^*))j_{\mathcal{C}r}^* + (h_{\mathcal{P}}'^* + h_{\mathcal{C}}'^*)j_{\mathcal{C}\theta}^*\right)\right|_{r^*=h_{\mathcal{P}}^*+h_{\mathcal{C}}^*} = 0, \tag{69}$$

where $\mathrm{Pe}_{\mathcal{C}} = R_0 U_{\mathcal{C}}/D$ is the Péclet number of the choroidal tissue. Eq. (68a) implies that r^*-dependent variations of $c_{\mathcal{C}}^*$ occurs at $O(\epsilon_{\mathcal{C}}^2 \mathrm{Pe}_{\mathcal{C}})$. We check the reduced Péclet number:

$$\epsilon_{\mathcal{C}}^2 \mathrm{Pe}_{\mathcal{C}} = \frac{Q_0 h_{\mathcal{C}0}}{2\pi R_0^2 D} \approx \frac{(0.25\times 10^{-9}/60)\times(2.66\cdot 10^{-4})}{2\pi\times 0.0115^2\times(61\cdot 10^{-12})} = 0.02 \quad (1\,\mathrm{s.f.}), \tag{70}$$

which is small, confirming that $c_{\mathcal{C}}$ is independent of r to leading order. Neglecting corrections of $O(\epsilon_{\mathcal{C}})$, we solve (67), using (68b) and (69) to get

$$j_{\mathcal{C}r}^* = \frac{1}{\sin\theta}\frac{\partial}{\partial\theta}\left((h_{\mathcal{P}}^* + h_{\mathcal{C}}^* - r^*)\left(c_{\mathcal{C}}^*\frac{\partial p_{\mathcal{C}}^*}{\partial\theta} + \frac{1}{\mathrm{Pe}_{\mathcal{C}}}\frac{\partial c_{\mathcal{C}}^*}{\partial\theta}\right)\sin\theta\right) + s_{\mathcal{C}}^*(h_{\mathcal{P}}^* + h_{\mathcal{C}}^* - r^*). \tag{71}$$

Redimensionalising,

$$j_{\mathcal{C}r} = \frac{1}{R_0^2\sin\theta}\frac{\partial}{\partial\theta}\left((r + h_{\mathcal{P}} + h_{\mathcal{C}} - R_0)\left(\frac{k_{\mathcal{C}}}{\mu}c_{\mathcal{C}}\frac{\partial p_{\mathcal{C}}}{\partial\theta} + D\frac{\partial c_{\mathcal{C}}}{\partial\theta}\right)\sin\theta\right) + s_{\mathcal{C}}(r + h_{\mathcal{P}} + h_{\mathcal{C}} - R_0), \tag{72}$$

and

$$j_{\mathcal{C}\theta} = -\frac{1}{R_0}\left(\frac{k_{\mathcal{C}}}{\mu}c_{\mathcal{C}}\frac{\partial p_{\mathcal{C}}}{\partial\theta} + D\frac{\partial c_{\mathcal{C}}}{\partial\theta}\right). \tag{73}$$

As with the flow, the remainder of the analysis of the concentrations depends on the case; we now consider Cases (i)–(iii) in turn.

4.2 Analysis of transport in Case (i)

In Case (i), we assume no SCS so need only apply suitable boundary conditions at the sclera $r = R_0$. We apply the boundary condition (21a) to give

$$\frac{1}{R_0^2\sin\theta}\frac{\mathrm{d}}{\mathrm{d}\theta}\left(h_{\mathcal{C}}\left(\frac{k_{\mathcal{C}}}{\mu}c\frac{\mathrm{d}p}{\mathrm{d}\theta} + D\frac{\mathrm{d}c}{\mathrm{d}\theta}\right)\sin\theta\right) + h_{\mathcal{C}} s_{\mathcal{C}} = s_{\mathcal{S}}, \tag{74}$$

where we have set $c_{\mathcal{C}} = c$.

The total albumin flow rate is a function of θ, given by

$$\begin{aligned} J =& \int_{R_0-h_{\mathcal{C}}}^{R_0} 2\pi r \sin\theta \left(-j_{\mathcal{C}\theta}\right) \, \mathrm{d}r, \\ =& 2\pi \sin\theta \left(\frac{k_{\mathcal{C}} h_{\mathcal{C}}}{\mu} c \frac{\mathrm{d}p}{\mathrm{d}\theta} + D h_{\mathcal{C}} \frac{\mathrm{d}c}{\mathrm{d}\theta} \right), \\ =& cQ + 2\pi D h_{\mathcal{C}} \sin\theta \frac{\mathrm{d}c}{\mathrm{d}\theta}, \end{aligned} \tag{75}$$

and hence

$$\frac{\mathrm{d}c}{\mathrm{d}\theta} = \frac{J - cQ}{2\pi D h_{\mathcal{C}} \sin\theta}, \tag{76}$$

$$\frac{\mathrm{d}J}{\mathrm{d}\theta} = - 2\pi R_0^2 \sin\theta \left(h_{\mathcal{C}} s_{\mathcal{C}} - s_{\mathcal{S}} \right), \tag{77}$$

with the boundary conditions (26) giving $J = cQ$ at both the inlet $\theta = \theta_0$ and the pole $\theta = 0$.

4.3 Analysis of transport in Case (ii)

In Case (ii), we model the SCS as a porous medium.

4.3.1 Transport in the SCS in Case (ii)

Scaling the variables as in Section 4.1, the governing equations (19) and boundary conditions (21a) become, respectively,

$$-\frac{1}{(1-\epsilon_{\mathcal{C}} r^*)^2} \frac{\partial}{\partial r^*} (1-\epsilon_{\mathcal{C}} r^*)^2 j^*_{\mathcal{P}r} + \frac{1}{(1-\epsilon_{\mathcal{C}} r^*)\sin\theta} \frac{\partial}{\partial\theta} j^*_{\mathcal{P}\theta} \sin\theta = 0, \tag{78}$$

$$j^*_{\mathcal{P}r} = \frac{1}{\epsilon_{\mathcal{C}}^2} \left(k^*_{\mathcal{P}} c^*_{\mathcal{P}} \frac{\partial p^*_{\mathcal{P}}}{\partial r^*} + \frac{1}{\mathrm{Pe}_{\mathcal{P}}} \frac{\partial c^*_{\mathcal{P}}}{\partial r^*} \right), \tag{79}$$

$$j^*_{\mathcal{P}\theta} = -\frac{1}{1-\epsilon_{\mathcal{C}} r^*} \left(k^*_{\mathcal{P}} c^*_{\mathcal{P}} \frac{\partial p^*_{\mathcal{P}}}{\partial \theta} + \frac{1}{\mathrm{Pe}_{\mathcal{P}}} \frac{\partial c^*_{\mathcal{P}}}{\partial \theta} \right), \tag{80}$$

$$j^*_{\mathcal{P}r}|_{r^*=0} = s^*_{\mathcal{S}}, \tag{81}$$

where $s_{\mathcal{S}} = \epsilon_{\mathcal{C}} U_{\mathcal{C}} c_0 s^*_{\mathcal{S}}$ and $\mathrm{Pe}_{\mathcal{P}} = R_0 U_{\mathcal{C}}/D$ is the Péclet number of the flow in the SCS. Eq. (80a) implies that r^* dependent variations of $c^*_{\mathcal{P}}$ occur at $O(\epsilon_{\mathcal{C}}^2 \mathrm{Pe}_{\mathcal{P}})$. The reduced Péclet number in the SCS is around 0.2, which is small, meaning that $c_{\mathcal{C}}$ is independent of r to leading order. Neglecting corrections of $O(\epsilon_{\mathcal{C}})$, we solve (78), using (80b) and (81) to get

$$j^*_{\mathcal{P}r} = -\frac{r^*}{\sin\theta} \frac{\partial}{\partial\theta} \left(\left(k^*_{\mathcal{P}} c^*_{\mathcal{P}} \frac{\partial p^*_{\mathcal{P}}}{\partial\theta} + \frac{1}{\mathrm{Pe}_{\mathcal{P}}} \frac{\partial c^*_{\mathcal{P}}}{\partial\theta} \right) \sin\theta \right) + s^*_{\mathcal{S}}. \tag{82}$$

Redimensionalising,

$$j_{\mathcal{P}r} = \frac{(r-R_0)}{R_0^2 \sin\theta} \frac{\partial}{\partial\theta} \left(\left(\frac{k_{\mathcal{P}}}{\mu} c_{\mathcal{P}} \frac{\partial p_{\mathcal{P}}}{\partial\theta} + D \frac{\partial c_{\mathcal{P}}}{\partial\theta} \right) \sin\theta \right) + s_{\mathcal{S}}, \tag{83}$$

and

$$j_{\mathcal{P}\theta} = -\frac{1}{R_0} \left(\frac{k_{\mathcal{P}}}{\mu} c_{\mathcal{P}} \frac{\partial p_{\mathcal{P}}}{\partial\theta} + D \frac{\partial c_{\mathcal{P}}}{\partial\theta} \right). \tag{84}$$

4.3.2 Matching conditions for transport in Case (ii)

We now match between the choroidal tissue and SCS, applying the interface conditions (22). Eq. (22a) implies $c_{\mathcal{C}} = c_{\mathcal{P}} = c$, and, at leading order, (22b) becomes

$$j_{\mathcal{C}r} + \frac{1}{R_0} h'_{\mathcal{P}} j_{\mathcal{C}\theta}\Big|_{r=R_0-h_{\mathcal{P}}-} = j_{\mathcal{P}r} + \frac{1}{R_0} h'_{\mathcal{P}} j_{\mathcal{P}\theta}\Big|_{r=R_0-h_{\mathcal{P}}+}, \tag{85}$$

giving

$$\frac{1}{R_0^2 \sin\theta} \frac{\mathrm{d}}{\mathrm{d}\theta} \left(\left(\frac{1}{\mu} \left(h_{\mathcal{C}} k_{\mathcal{C}} + h_{\mathcal{P}} k_{\mathcal{P}} \right) c \frac{\mathrm{d}p}{\mathrm{d}\theta} + \left(h_{\mathcal{C}} D + h_{\mathcal{P}} D \right) \frac{\mathrm{d}c}{\mathrm{d}\theta} \right) \sin\theta \right) + h_{\mathcal{C}} s_{\mathcal{C}} - s_{\mathcal{S}} = 0. \tag{86}$$

The total albumin flow rate is a function of theta, given by

$$\begin{aligned} J = & \int_{R_0-h_{\mathcal{P}}-h_{\mathcal{C}}}^{R_0-h_{\mathcal{P}}} 2\pi r \sin\theta \left(-j_{\mathcal{C}\theta}\right) \mathrm{d}r + \int_{R_0-h_{\mathcal{P}}}^{R_0} 2\pi r \sin\theta \left(-j_{\mathcal{P}\theta}\right) \mathrm{d}r \\ =& 2\pi \sin\theta \left(\frac{k_{\mathcal{C}} h_{\mathcal{C}} + k_{\mathcal{P}} h_{\mathcal{P}}}{\mu} c \frac{\mathrm{d}p}{\mathrm{d}\theta} + D h_{\mathcal{C}} \frac{\mathrm{d}c}{\mathrm{d}\theta} + D h_{\mathcal{P}} \frac{\mathrm{d}c}{\mathrm{d}\theta} \right) \\ =& cQ + 2\pi \sin\theta \left(D h_{\mathcal{C}} + D h_{\mathcal{P}} \right) \frac{\mathrm{d}c}{\mathrm{d}\theta}, \end{aligned} \tag{87}$$

and hence

$$\frac{\mathrm{d}c}{\mathrm{d}\theta} = \frac{J - cQ}{2\pi \sin\theta (D h_{\mathcal{C}} + D h_{\mathcal{P}})}, \tag{88}$$

$$\frac{\mathrm{d}J}{\mathrm{d}\theta} = -\, 2\pi R_0^2 \sin\theta \left(h_{\mathcal{C}} s_{\mathcal{C}} - s_{\mathcal{S}} \right), \tag{89}$$

with $J = cQ$ at $\theta = 0, \theta_0$.

4.4 Analysis of transport in Case (iii)

In Case (iii), we model the SCS as open.

4.4.1 Transport in the SCS in Case (iii)

In the SCS, we scale the fluxes according to

$$c_{\mathcal{P}} = c_0 c_{\mathcal{P}}^\dagger, \quad j_{\mathcal{P}r} = \epsilon_{\mathcal{P}} U_{\mathcal{P}} c_0 j_{\mathcal{P}r}^\dagger, \quad j_{\mathcal{P}\theta} = U_{\mathcal{P}} c_0 j_{\mathcal{P}\theta}^\dagger, \quad s_{\mathcal{S}} = \epsilon_{\mathcal{P}} U_{\mathcal{P}} c_0 s_{\mathcal{S}}^\dagger. \tag{90}$$

The governing equation (19) and boundary conditions (21a) become, respectively,

$$-\frac{1}{(1-\epsilon_{\mathcal{P}} r^\dagger)^2} \frac{\partial}{\partial r^\dagger} \left((1-\epsilon_{\mathcal{P}} r^\dagger)^2 j_{\mathcal{P}r}^\dagger \right) + \frac{1}{(1-\epsilon_{\mathcal{P}} r^\dagger)\sin\theta} \frac{\partial}{\partial\theta} \left(j_{\mathcal{P}\theta}^\dagger \sin\theta \right) = 0, \tag{91}$$

$$j_{\mathcal{P}r}^\dagger = u_{\mathcal{P}r}^\dagger c_{\mathcal{P}}^\dagger + \frac{1}{\epsilon_{\mathcal{P}}^2 \mathrm{Pe}_{\mathcal{P}}} \frac{\partial c_{\mathcal{P}}^\dagger}{\partial r^\dagger}, \quad j_{\mathcal{P}\theta}^\dagger = -\frac{1}{2} r^\dagger (h_{\mathcal{P}}^\dagger - r^\dagger) c_{\mathcal{P}}^\dagger \frac{\partial p_{\mathcal{P}}^\dagger}{\partial\theta} - \frac{1}{(1-\epsilon_{\mathcal{P}} r^\dagger)\mathrm{Pe}_{\mathcal{P}}} \frac{\partial c_{\mathcal{P}}^\dagger}{\partial\theta}, \tag{92}$$

$$j_{\mathcal{P}r}^\dagger\Big|_{r^\dagger=0} = s_{\mathcal{S}}^\dagger. \tag{93}$$

As long as $\epsilon_{\mathcal{P}}^2 \mathrm{Pe}_{\mathcal{P}} \ll 1$, we may neglect the terms $j_{\mathcal{P}r}^\dagger$ and $u_{\mathcal{P}r}^\dagger c_{\mathcal{P}}^\dagger$ appearing in Eq. (92a), and we find that $c_{\mathcal{P}}^\dagger$ is independent of $r^\dagger$ to leading order, $O(\epsilon_{\mathcal{P}}^2 \mathrm{Pe}_{\mathcal{P}})$. We estimate as follows:

$$\epsilon_{\mathcal{P}}^2 \mathrm{Pe}_{\mathcal{P}} = \frac{h_{\mathcal{P}0}^2}{R_0^2} \times \frac{R_0 U_{\mathcal{P}}}{D} = \frac{Q_0 h_{\mathcal{P}0}^5}{2\pi R_0^3 h_{\mathcal{C}0} D k_{\mathcal{C}}} \approx 0.1 \left(\frac{h_{\mathcal{P}0}\,(\mu\mathrm{m})}{21} \right)^5, \tag{94}$$

which suggests $h_{\mathcal{P}0}$ must be less than about 21 μm for this argument to be correct to within 10%. For the value we actually use, $h_{\mathcal{P}0} = 2.4$ μm, $\epsilon_{\mathcal{P}}^2 \mathrm{Pe}_{\mathcal{P}} \approx 2 \times 10^{-6}$, suggesting the approximation is a very good one. We solve (91) at leading order, using (92b) and (93) to obtain

$$j^{\dagger}_{\mathcal{P}r} = -\frac{1}{\sin\theta}\frac{\partial}{\partial\theta}\left(\left(\frac{1}{12}r^{\dagger 2}(3h^{\dagger}_{\mathcal{P}} - 2r^{\dagger})c^{\dagger}_{\mathcal{P}}\frac{\partial p^{\dagger}_{\mathcal{P}}}{\partial\theta} + \frac{r^{\dagger}}{\mathrm{Pe}_{\mathcal{P}}}\frac{\partial c^{\dagger}_{\mathcal{P}}}{\partial\theta}\right)\sin\theta\right) + s^{\dagger}_{\mathcal{S}}. \tag{95}$$

Re-dimensionalising, we obtain

$$\begin{aligned} j_{\mathcal{P}r} = -\frac{1}{R_0^2\sin\theta}\frac{\partial}{\partial\theta}\Bigg(\Bigg(\frac{1}{6\mu}(R_0 - r)^2\left(r - \left(R_0 - \frac{3}{2}h_{\mathcal{P}}\right)\right)c_{\mathcal{P}}\frac{\partial p_{\mathcal{P}}}{\partial\theta} \\ +D(R_0 - r)\frac{\partial c_{\mathcal{P}}}{\partial\theta}\Bigg)\sin\theta\Bigg) + s_{\mathcal{S}}, \end{aligned} \tag{96}$$

$$j_{\mathcal{P}\theta} = -\frac{1}{R_0}\left(\frac{1}{2\mu}(R_0 - r)\left(r - (R_0 - h_{\mathcal{P}})\right)c_{\mathcal{P}}\frac{\partial p_{\mathcal{P}}}{\partial\theta} + D\frac{\partial c_{\mathcal{P}}}{\partial\theta}\right). \tag{97}$$

4.4.2 Matching conditions for transport in Case (iii)

As in Case (ii) we match at the interface between the SCS and choroid. The interface condition (22b) implies that

$$\frac{1}{R_0^2\sin\theta}\frac{\mathrm{d}}{\mathrm{d}\theta}\left(\left(\frac{k_{\mathcal{C}}h_{\mathcal{C}}}{\mu}c_{\mathcal{C}}\frac{\mathrm{d}p_{\mathcal{C}}}{\mathrm{d}\theta} + \frac{h_{\mathcal{P}}^3}{12\mu}c_{\mathcal{P}}\frac{\mathrm{d}p_{\mathcal{P}}}{\mathrm{d}\theta} + Dh_{\mathcal{C}}\frac{\mathrm{d}c_{\mathcal{C}}}{\mathrm{d}\theta} + Dh_{\mathcal{P}}\frac{\mathrm{d}c_{\mathcal{P}}}{\mathrm{d}\theta}\right)\sin\theta\right) + h_{\mathcal{C}}s_{\mathcal{C}} - s_{\mathcal{S}} = 0, \tag{98}$$

or, in non-dimensional terms,

$$\frac{1}{\sin\theta}\frac{\mathrm{d}}{\mathrm{d}\theta}\left(\left(Ch^*_{\mathcal{C}}c^*_{\mathcal{C}}\frac{\mathrm{d}p^*_{\mathcal{C}}}{\mathrm{d}\theta} + \frac{1}{12}h^{\dagger 3}_{\mathcal{P}}c^{\dagger}_{\mathcal{P}}\frac{\mathrm{d}p^{\dagger}_{\mathcal{P}}}{\mathrm{d}\theta} + \frac{C}{\mathrm{Pe}_{\mathcal{C}}}h^*_{\mathcal{C}}\frac{\mathrm{d}c^*_{\mathcal{C}}}{\mathrm{d}\theta} + \frac{1}{\mathrm{Pe}_{\mathcal{P}}}h^{\dagger}_{\mathcal{P}}\frac{\mathrm{d}c^{\dagger}_{\mathcal{P}}}{\mathrm{d}\theta}\right)\sin\theta\right) + Cs^*_{\mathcal{C}} - s^{\dagger}_{\mathcal{S}} = 0, \tag{99}$$

while condition (22a) implies that $c_{\mathcal{C}} = c_{\mathcal{P}} = c$; these constraints are to be solved together with conditions (26) (note that, as with the fluid flow derivation, two different sets of nondimensional scales appear in Eq. (99)).

The total flow rate of albumin is a function of θ, given by

$$\begin{aligned} J =& \int_{R_0-h_{\mathcal{P}}-h_{\mathcal{C}}}^{R_0-h_{\mathcal{P}}} 2\pi r\sin\theta\,(-j_{\mathcal{C}\theta})\ \mathrm{d}r + \int_{R_0-h_{\mathcal{P}}}^{R_0} 2\pi r\sin\theta\,(-j_{\mathcal{P}\theta})\ \mathrm{d}r \\ =& 2\pi\sin\theta\left(\frac{k_{\mathcal{C}}h_{\mathcal{C}}}{\mu}c\frac{\mathrm{d}p}{\mathrm{d}\theta} + \frac{h_{\mathcal{P}}^3}{12\mu}c\frac{\mathrm{d}p}{\mathrm{d}\theta} + Dh_{\mathcal{C}}\frac{\mathrm{d}c}{\mathrm{d}\theta} + Dh_{\mathcal{P}}\frac{\mathrm{d}c}{\mathrm{d}\theta}\right) \\ =& cQ + 2\pi\sin\theta\,(Dh_{\mathcal{C}} + Dh_{\mathcal{P}})\frac{\mathrm{d}c}{\mathrm{d}\theta}, \end{aligned} \tag{100}$$

and hence

$$\frac{\mathrm{d}c}{\mathrm{d}\theta} = \frac{J - cQ}{2\pi\sin\theta(Dh_{\mathcal{C}} + Dh_{\mathcal{P}})}, \tag{101}$$

$$\frac{\mathrm{d}J}{\mathrm{d}\theta} = -2\pi R_0^2\sin\theta\,(h_{\mathcal{C}}s_{\mathcal{C}} - s_{\mathcal{S}}), \tag{102}$$

with $J = cQ$ at $\theta = 0, \theta_0$.

5 Summary

In this Section, we review and summarize the governing equations and boundary conditions that were derived in Sections 3 and 4. Since we consider the choroidal tissue and SCS together, it is convenient to define the term 'choroid–SCS' to denote both of these regions.

In each of the Cases (i)–(iii), the four governing equations may be written as:

$$\frac{\mathrm{d}p}{\mathrm{d}\theta} = \frac{\mu Q}{2\pi \sin\theta (k_{\mathcal{C}} h_{\mathcal{C}} + k_{\mathcal{P}} h_{\mathcal{P}}^{m})}, \tag{103}$$

$$\frac{\mathrm{d}Q}{\mathrm{d}\theta} = -\, 2\pi R_0^2 \sin\theta \left(h_{\mathcal{C}} q_{\mathcal{C}} + q_{\mathcal{R}} - q_{\mathcal{S}} \right), \tag{104}$$

$$\frac{\mathrm{d}c}{\mathrm{d}\theta} = \frac{J - cQ}{2\pi \sin\theta (D h_{\mathcal{C}} + D h_{\mathcal{P}})}, \tag{105}$$

$$\frac{\mathrm{d}J}{\mathrm{d}\theta} = -\, 2\pi R_0^2 \sin\theta \left(h_{\mathcal{C}} s_{\mathcal{C}} - s_{\mathcal{S}} \right), \tag{106}$$

with boundary conditions

$$Q = 0,\ J = cQ \text{ at } \theta = 0, \tag{107}$$

$$\mathrm{IOP} - p = R_{icm} Q,\ J = cQ \text{ at } \theta = \theta_0. \tag{108}$$

We account for all three Cases (i)–(iii) in this formulation, and definitions of all the symbols appearing in the equations and boundary conditions along with details of how to adapt the model to each case are given in Table 1. In this Table, we classify the expressions according to their mathematical type:

- independent variable: θ ranges from 0 to θ_0 (θ_0 must be specified);
- primary dependent variables: all functions of θ that are outputs of the model;
- parameters: fixed constants that are inputs to the model and must be specified by the user;
- parametric expression: $h_{\mathcal{C}}(\theta)$ is a fixed function of θ that must be specified by the user;
- subsidiary dependent variables: functions of the primary dependent variables and outputs of the model;
- the special case of $h_{\mathcal{P}}(\theta)$, which is zero in Case (i), and in Cases (ii) and (iii) in the physiological case it is equal to a fixed parameter, $h_{\mathcal{P}0}$. If a non-physiological solution is required, $h_{\mathcal{P}}$ is given by Eq. (3), which depends on $h_{\mathcal{P},\mathrm{nat}}(\theta)$, and this function must be found before solving.

Note that the equations have a removable singularity at $\theta = 0$ owing to division by $\sin\theta$.

Table 1: List of expressions appearing in the model equations and boundary conditions with descriptions. These are classified according to their type (see text for description). SI units are provided in some cases for clarity.

Symbol	Description
**Independent variable**	
θ	angle subtended at the center of coordinates between the point and posterior pole of the eye
**Primary dependent variables**	
$p(\theta)$	IF pressure in the choroid–SCS
$Q(\theta)$	IF flow rate along the choroid–SCS
$c(\theta)$	albumin concentration in the choroid–SCS
$J(\theta)$	albumin flow rate along the choroid–SCS
**Parameters**	
μ	dynamic viscosity of IF
$k_{\mathcal{C}}$	Darcy permeability of choroidal interstitial tissue
$k_{\mathcal{P}}$	Case (i): not applicable; Case (ii): Darcy permeability of SCS; Case (iii): equal to 1/12
m	Case (i): not applicable; Case (ii): equal to 1; Case (iii): equal to 3
R_0	inner scleral radius
$q_{\mathcal{R}}$	flux of fluid from RPE (m/s)
D	diffusion coefficient of albumin
μ	dynamic viscosity of IF
θ_0	angular extent of choroid and SCS from posterior pole
R_{icm}	resistance of the iris root and ciliary muscle (Pa s/m^3)
IOP	intraocular pressure
**Parametric expression**	
$h_{\mathcal{C}}(\theta)$	thickness of choroid
**Subsidiary dependent variables**	
$q_{\mathcal{C}}(\theta)$	flux of fluid out of CC (s^{-1}), see Eq. (2)
$q_{\mathcal{S}}(\theta)$	flux of fluid across sclera (m/s), see Eq. (13)
$s_{\mathcal{C}}(\theta)$	flux of albumin out of CC (mol/(m^{3}s)), see Eq. (23)
$s_{\mathcal{S}}(\theta)$	flux of albumin across sclera (mol/(m^{2}s)), see Eq. (24)
**Special**	
$h_{\mathcal{P}}(\theta)$	thickness of SCS: zero in Case (i), equal to a specified constant parameter in the physiological case, otherwise it is a subsidiary dependent variable, see Eq. (3)

To solve these equations numerically, we first non-dimensionalise with respect to the scalings

of Sections 3.1 and 4.1:

$$\frac{\mathrm{d}p^*}{\mathrm{d}\theta} =\frac{Q^*}{\sin\theta(h_{\mathcal{C}}^* + k_{\mathcal{P}}^* h_{\mathcal{P}}^{*m})}, \tag{109}$$

$$\frac{\mathrm{d}Q^*}{\mathrm{d}\theta} =-\sin\theta\left(h_{\mathcal{C}}^* q_{\mathcal{CC}}^* + q_{\mathcal{R}}^* - q_{\mathcal{S}}^*\right), \tag{110}$$

$$\frac{\mathrm{d}c^*}{\mathrm{d}\theta} =\frac{\mathrm{Pe}(J^* - c^* Q^*)}{\sin\theta(h_{\mathcal{C}}^* + h_{\mathcal{P}}^*)}, \tag{111}$$

$$\frac{\mathrm{d}J^*}{\mathrm{d}\theta} =-\sin\theta\left(h_{\mathcal{C}}^* s_{\mathcal{CC}}^* - s_{\mathcal{S}}^*\right), \tag{112}$$

subject to the boundary conditions

$$Q^* = J^* = 0 \text{ at } \theta = 0, \tag{113}$$

$$\mathrm{IOP}^* - p^* = R_{icm}^* Q^*,\ J^* = c^* Q^* \text{ at } \theta = \theta_0 \tag{114}$$

note that we do not use the scalings applied in the derivations of flow and transport in the SCS in our numerical model. In these equations, the non-dimensional fluxes are given by

$$q_{\mathcal{CC}}^* =L_{\mathcal{CC}}^*(p_{\mathcal{CC}}^* - p^* - \sigma_{\mathcal{CC}}^*(c_{\mathcal{CC}}^* - c^*)), \tag{115}$$

$$q_{\mathcal{S}}^* =k_{\mathcal{S}}^*(p^* - p_{\mathcal{O}}^* - \sigma_{\mathcal{S}}^*(c^* - c_{\mathcal{O}}^*)), \tag{116}$$

$$s_{\mathcal{CC}}^* =\beta_{\mathcal{CC}}^*(c_{\mathcal{CC}}^* - c^*) + (1-\sigma_{\mathcal{CC}})\frac{c^* + c_{\mathcal{CC}}^*}{2} q_{\mathcal{CC}}^*, \tag{117}$$

$$s_{\mathcal{S}}^* =q_{\mathcal{S}}^*(1-\sigma_{\mathcal{S}})\frac{c_{\mathcal{O}}^* - c^* e^{Pe_{\mathcal{S}}}}{1 - e^{Pe_{\mathcal{S}}}}, \tag{118}$$

In these equations, the non-dimensional variables are related to the dimensional variables of the model by

$$h_{\mathcal{C}} = h_{\mathcal{C}0} h_{\mathcal{C}}^*, \quad h_{\mathcal{P}} = h_{\mathcal{C}0} h_{\mathcal{P}}^*, \quad Q = Q_0 Q^*, \tag{119}$$

$$p = P_0 p^*, \quad p_{\mathcal{CC}} = P_0 p_{\mathcal{CC}}^*, \quad p_{\mathcal{O}} = P_0 p_{\mathcal{O}}^*, \quad \mathrm{IOP} = P_0 \mathrm{IOP}^*, \tag{120}$$

$$c = c_0 c^*, \quad c_{\mathcal{CC}} = c_0 c_{\mathcal{CC}}^*, \quad c_o = c_0 c_o^*, \tag{121}$$

$$J = Q_0 c_0 J^*, \quad R_{icm} = P_0 R_{icm}^* / Q_0, \tag{122}$$

and the constants appearing in the non-dimensional equations are given by

$$q_{\mathcal{R}}^* = \frac{2\pi R_0^2}{Q_0} q_{\mathcal{R}}, \quad k_{\mathcal{P}}^* = \begin{cases} k_{\mathcal{P}}/k_{\mathcal{C}} \text{ if } m = 1 \\ h_{\mathcal{C}0}^2/(12 k_{\mathcal{C}}) \text{ if } m = 3 \end{cases}, \quad \mathrm{Pe} = \frac{Q_0}{2\pi h_{\mathcal{C}0} D}, \tag{123}$$

$$L_{\mathcal{CC}}^* = \frac{\mu R_0^2}{k_{\mathcal{C}}} L_{\mathcal{CC}}, \quad \sigma_{\mathcal{CC}}^* = \frac{\sigma_{\mathcal{CC}} R_g T c_0}{P_0}, \quad k_{\mathcal{S}}^* = \frac{R_0^2 k_{\mathcal{S}}}{h_{\mathcal{C}0} h_{\mathcal{S}} k_{\mathcal{C}}}, \tag{124}$$

$$\beta_{\mathcal{CC}}^* = \frac{2\pi R_0^2 h_{\mathcal{C}0}}{Q_0} \beta_{\mathcal{CC}}, \quad \sigma_{\mathcal{S}}^* = \frac{\sigma_{\mathcal{S}} R_g T c_0}{P_0}, \tag{125}$$

where $c_0 = c_{\mathcal{CC}}$.

We solve the non-dimensional equations numerically using the MATLAB R2024a (Mathworks) solver `bvp4c`. Note that we need to use the nondimensional equations, as the orders of magnitude of the different terms in the dimensional equations are so different that the solver does not converge to a satisfactory solution when using the dimensional variables. In non-physiological cases, we need to run the solver twice, first for the physiological case (with $h_{\mathcal{P}}$ equal to a fixed constant $h_{\mathcal{P}0}$) to find the function $h_{\mathcal{P},\mathrm{nat}}(\theta)$, and then (with the appropriate parameter values changed) to find the solution. We also avoided numerical singularities at $\theta = 0$ by solving on the domain $[\delta\theta, \theta_0]$, where $\delta\theta = \theta_0/n_{\mathrm{pts}}$ is the grid spacing and n_{pts} is the number of grid points in θ. Throughout the main paper we redimensionalise all variables before presenting our results.

6 Use of a Beavers–Joseph boundary condition

In the paper, we assumed a no-slip boundary condition (14) on the IF in the SCS at both the interfaces with the sclera and the choroid; however, it is common to use a Beavers–Joseph condition at the interface between a freely flowing fluid and the fluid within a porous medium.[9] This states that the shear rate of the shear rate at the boundary equals the discontinuity in the fluid velocity between the free fluid and that in the porous medium divided by the square root of the permeability of the porous medium, multiplied by a dimensionless slip constant. In our analysis, to leading order in the small parameters $\epsilon_{\mathcal{C}}$ and $\epsilon_{\mathcal{P}}$ and, neglecting the fluid velocity in the porous medium as it is small, the boundary conditions at the SCS–sclera and SCS–choroid interfaces may be stated

$$u_{\mathcal{P}\theta} = \frac{\sqrt{k_{\mathcal{S}}}}{\alpha_{\mathcal{S}}}\frac{\partial u_{\mathcal{P}\theta}}{\partial r}, \quad u_{\mathcal{P}\theta} = -\frac{\sqrt{k_{\mathcal{C}}}}{\alpha_{\mathcal{C}}}\frac{\partial u_{\mathcal{P}\theta}}{\partial r}, \tag{126}$$

at $r = R$, and at $r = R - h_{\mathcal{P}}$, respectively, where $\alpha_{\mathcal{S}}$ and $\alpha_{\mathcal{C}}$ are dimensionless positive slip constants that depend only on the properties of the porous materials comprising the sclera and choroid (the no-slip case considered earlier may be recovered in the limit $\alpha_{\mathcal{S}}, \alpha_{\mathcal{C}} \to \infty$). The analysis proceeds in a similar way, and we obtain governing equations similar to Equations (103)–(106), but with Equation (103) replaced by

$$\frac{\mathrm{d}p}{\mathrm{d}\theta} = \frac{\mu Q}{2\pi \sin\theta (k_{\mathcal{C}} h_{\mathcal{C}} + h_{\mathcal{P}}^2 \tilde{h}_{\mathcal{P}}/12)}, \tag{127}$$

where

$$\tilde{h}_{\mathcal{P}} = \frac{(h_{\mathcal{P}}^2 + 4C_{\mathcal{C}} h_{\mathcal{P}0} h_{\mathcal{P}} + 4C_{\mathcal{S}} h_{\mathcal{P}0} h_{\mathcal{P}} + 12 C_{\mathcal{C}} C_{\mathcal{S}} h_{\mathcal{P}0}^2)}{(h_{\mathcal{P}} + C_{\mathcal{S}} h_{\mathcal{P}0} + C_{\mathcal{C}} h_{\mathcal{P}0})}, \tag{128}$$

with

$$C_{\mathcal{S}} = \frac{\sqrt{k_{\mathcal{S}}}}{h_{\mathcal{P}0}\alpha_{\mathcal{S}}} \approx \frac{1.0 \cdot 10^{-3}}{\alpha_{\mathcal{S}}}, \quad C_{\mathcal{C}} = \frac{\sqrt{k_{\mathcal{C}}}}{h_{\mathcal{P}0}\alpha_{\mathcal{C}}} \approx \frac{0.045}{\alpha_{\mathcal{C}}}. \tag{129}$$

Slip constants at the interface of a freely flowing fluid and a porous medium are typically of order one,[9,10] meaning that the change in the solution with this boundary condition is likely to be fairly small.

7 Fitting the choroidal thickness profile

Our expression for the choroidal thickness profile was derived by fitting a cubic polynomial (using MATLAB's curve fitting toolbox, with default settings) to measurements in human emmetropes;[11] that is, individuals with no refractive error or visual defects. The choroidal thickness was found to be 394 ± 70 μm at the fovea (0 to 1 mm eccentricity), 386 ± 68 μm in the parafovea (1 to 3 mm eccentricity), 368 ± 61 μm in the perifovea (3 to 5 mm eccentricity), 329 ± 51 μm in the near-periphery (5 to 8 mm eccentricity), and 277 ± 37 μm in the periphery (8 to 14 mm eccentricity). From inspection of a physiological image of an eye, the eccentricity of the iris root, θ_0, is about 140 degrees, and it is commonly agreed that the choroidal thickness there is around 100 μm. We represented these six regions as having respective eccentricities of 0, 3, 5, 8, 14 and 28.1 mm, equivalent to 0, 0.25, 0.42, 0.67, 1.17, 2.44 rad. We used the minimum eccentricity in the fovea, since the choroidal thickness is essentially uniform in this region, but the maximum eccentricity for the other retinal regions since, from inspection of Figure 2 of Hoseini–Yazdi *et al.* (2019), the average choroidal width is achieved (or comes close to being achieved) on these outer boundaries. Results are shown in Figure S3. We take the typical choroidal thickness as approximately the volume of the choroid divided by its surface area, setting $h_{\mathcal{C}0} = 266$ μm.

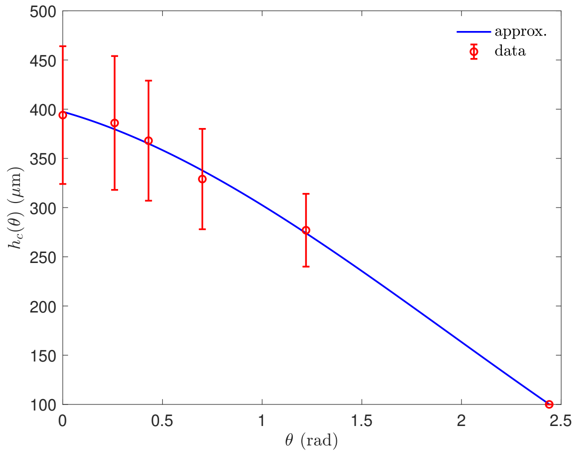

Figure S3: Choroidal thickness in human emmetropes ($n = 14$) at different locations, compared with a fitted cubic polynomial approximation.[11]

References

[1] C. Cancelli and T. J. Pedley, "A separated-flow model for collapsible-tube oscillations," *J. Fluid Mech.*, vol. 157, pp. 375–404, 1985.

[2] B. S. Brook, S. A. E. G. Falle, and T. J. Pedley, "Numerical solutions for unsteady gravity-driven flows in collapsible tubes: evolution and roll-wave instability of a steady state," *J. Fluid Mech.*, vol. 396, pp. 223–256, 1999.

[3] E. F. Toro and A. Siviglia, "Simplified blood flow model with discontinuous vessel properties: analysis and exact solutions," in *Modeling of Physiological Flows: Modeling, Simulation and Applications* (G. Ambrosi, A. Quarteroni, and G. Rozza, eds.), vol. 5, pp. 19–39, Milano: Springer, 2012.

[4] E. F. Toro, "Brain venous haemodynamics, neurological diseases and mathematical modelling: A review," *Appl. Math. Comput.*, vol. 272, pp. 542–579, 2016.

[5] O. Kedem and A. Katchalsky, "Thermodynamic analysis of the permeability of biological membranes to nonelectrolytes," *Biochim. Biophys. Acta*, vol. 27, pp. 229—246, 1958.

[6] A. Katchalsky and P. F. Curran, *Non-equilibrium Thermodynamics in Biophysics*. Cambridge, MA: Harvard University Press, 1965.

[7] O. Kedem and A. Katchalsky, "Permeability of composite membranes. Part 1. – Electric current, volume flow and flow of solute through membranes," *Trans. Faraday Soc.*, vol. 59, pp. 1918–1930, 1963.

[8] G. A. Truskey, F. Yuan, and D. F. Katz, *Transport Phenomena in Biological Systems*. Pearson Education, 2004.

[9] G. S. Beavers and D. D. Joseph, "Boundary conditions at a naturally permeable wall," *J. Fluid Mech.*, vol. 30, no. 1, pp. 197—-207, 1967.

[10] M. Mierzwiczak, A. Fraska, and J. K. Grabski, "Determination of the slip constant in the beavers-joseph experiment for laminar fluid flow through porous media using a meshless method," *Math. Probl. Eng.*, vol. 2019, p. 1494215, 2019.

[11] M. Hoseini-Yazdi, S. J. Vincent, M. J. Collins, S. A. Read, and D. Alonso-Caneiro, "Wide-field choroidal thickness in myopes and emmetropes," *Sci. Rep.*, vol. 9, p. 3474, 2019.
